# Photolabile coumarins with improved efficiency through azetidinyl substitution†
†Electronic supplementary information (ESI) available: Experimental details, ESI figures and NMR spectra. See DOI: 10.1039/c7sc03627b


**DOI:** 10.1039/c7sc03627b

**Published:** 2017-10-31

**Authors:** Giovanni Bassolino, Christoph Nançoz, Zacharias Thiel, Estelle Bois, Eric Vauthey, Pablo Rivera-Fuentes

**Affiliations:** a Laboratorium für Organische Chemie , ETH Zürich , HCI G329, Vladimir-Prelog-Weg 3 , 8093 Zürich , Switzerland . Email: pablo.rivera-fuentes@org.chem.ethz.ch; b Department of Physical Chemistry , University of Geneva , 30 Quai Ernest-Ansermet , 1211 Geneva 4 , Switzerland . Email: Eric.Vauthey@unige.ch

## Abstract

Azetidinyl substituents have been recently used to improve the fluorescence quantum yield of several classes of fluorophores. Herein, we demonstrate that other useful photochemical processes can be modulated using this strategy. In particular, we prepared and measured the quantum yield of photorelease of a series of 7-azetidinyl-4-methyl coumarin esters and compared it to their 7-diethylamino and julolidine-fused analogues. The efficiency of the photorelease reactions of the azetidinyl-substituted compounds was 2- to 5-fold higher than the corresponding diethylamino coumarins. We investigated the origin of this effect in model fluorophores and in the photoactivatable esters, and found that H-bonding with the solvent seems to be the prominent deactivation channel inhibited upon substitution with an azetidinyl ring. We anticipate that this substitution strategy could be used to modulate other photochemical processes with applications in chemical biology, catalysis and materials science.

## Abstract 2

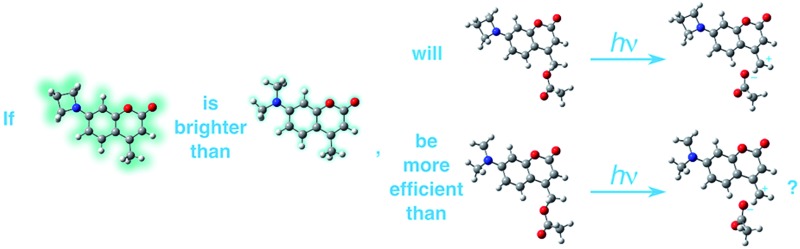
The efficiency of photoactivatable coumarins in water has been enhanced by substitution with azetidine.

## Introduction

Photochemical processes have been used extensively in a wide range of fields, including chemical biology,^1^ organic synthesis,^2^,^3^ and materials science,^3^ to gain selectivity and spatiotemporal control over the studied phenomena. The successful application of photochemical strategies relies on the ability of researchers to control the outcome of photoinduced reactions, by tuning chromophore parameters like absorption maxima, reaction selectivity or quantum efficiencies. A commonly adopted approach to modulate the photochemical evolutions of molecules consists in exploring structural variations of an already functioning scaffold^4^–^6^ to look for new motifs or substituents that enhance the desired properties.

**Chart 1:**
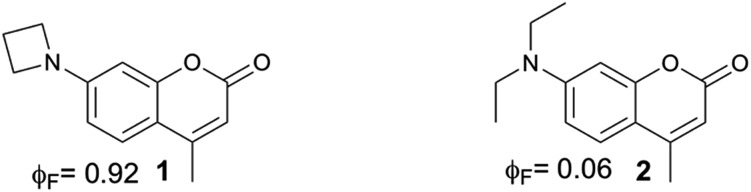
Structures of azetidinyl- and diethylaminocoumarins. The fluorescence quantum yields (*φ*_F_) shown were measured in H_2_O. In ref. 7 the reported *φ*_F_ in aqueous HEPES buffer (pH 7.3) for **1** and for 7-dimethylamino-4-methylcoumarin are 0.96 and 0.19, respectively. HEPES = 4-(2-hydroxyethyl)-1-piperazineethanesulfonic acid.

Recently, Lavis and co-workers demonstrated that azetidinyl-substituted fluorophores such as **1** (*λ*_abs_ = 355 nm; *λ*_em_ = 471 nm; Chart 1) display larger quantum yields of fluorescence (*φ*_F_) compared to those substituted with open-chain analogues, such as dimethylamino or diethylamino (compound **2**, *λ*_abs_ = 381 nm; *λ*_em_ = 468 nm), or larger cyclic amines, such as pyrrolidinyl or piperidinyl.^7^ Subsequently, Xu and co-workers found that aziridinyl-substituted fluorophores behave similarly.^8^ This improvement in *φ*_F_ values has been attributed to a decrease in the rate of population of twisted intramolecular charge transfer (TICT) states^9^ upon excitation.^7^,^8^ We hypothesized that if these small heterocyclic rings suppress competitive decay channels that affect emission, the same effect could be exploited to improve the efficiency of other important, non-emissive photochemical processes.

**Scheme 1:**
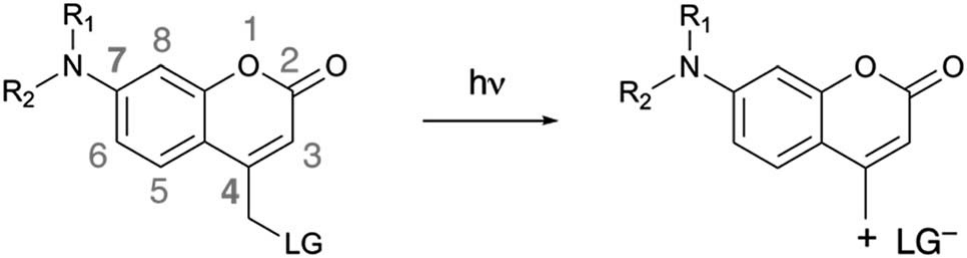
Photoinduced heterolytic cleavage of donor-substituted coumarins. R_1_, R_2_ = alkyl chains, cyclic or acyclic. LG = leaving group.

4-Methylcoumarin derivatives are widely used as fluorophores and photocleavable (also known as “caging”) groups.^10^,^11^ Electron-rich coumarins may undergo photoinduced heterolytic cleavage when C4 is substituted with a methylene bearing a good leaving group (Scheme 1). The quantum yields of photoactivation (*φ*_PA_) of such coumarins are usually moderate to low^11^–^17^ and depend on the nucleofugality of the leaving group.^11^ It would be advantageous to be able to improve the efficiency of these photocleavable groups, in particular in a manner that is independent from the nucleofugality of the leaving group. Herein we report that azetidinyl substitution improves the efficiency of *φ*_PA_ of a series of photocleavable coumarins with leaving groups of diverse nucleofugality.

## Results and discussion

**Chart 2:**
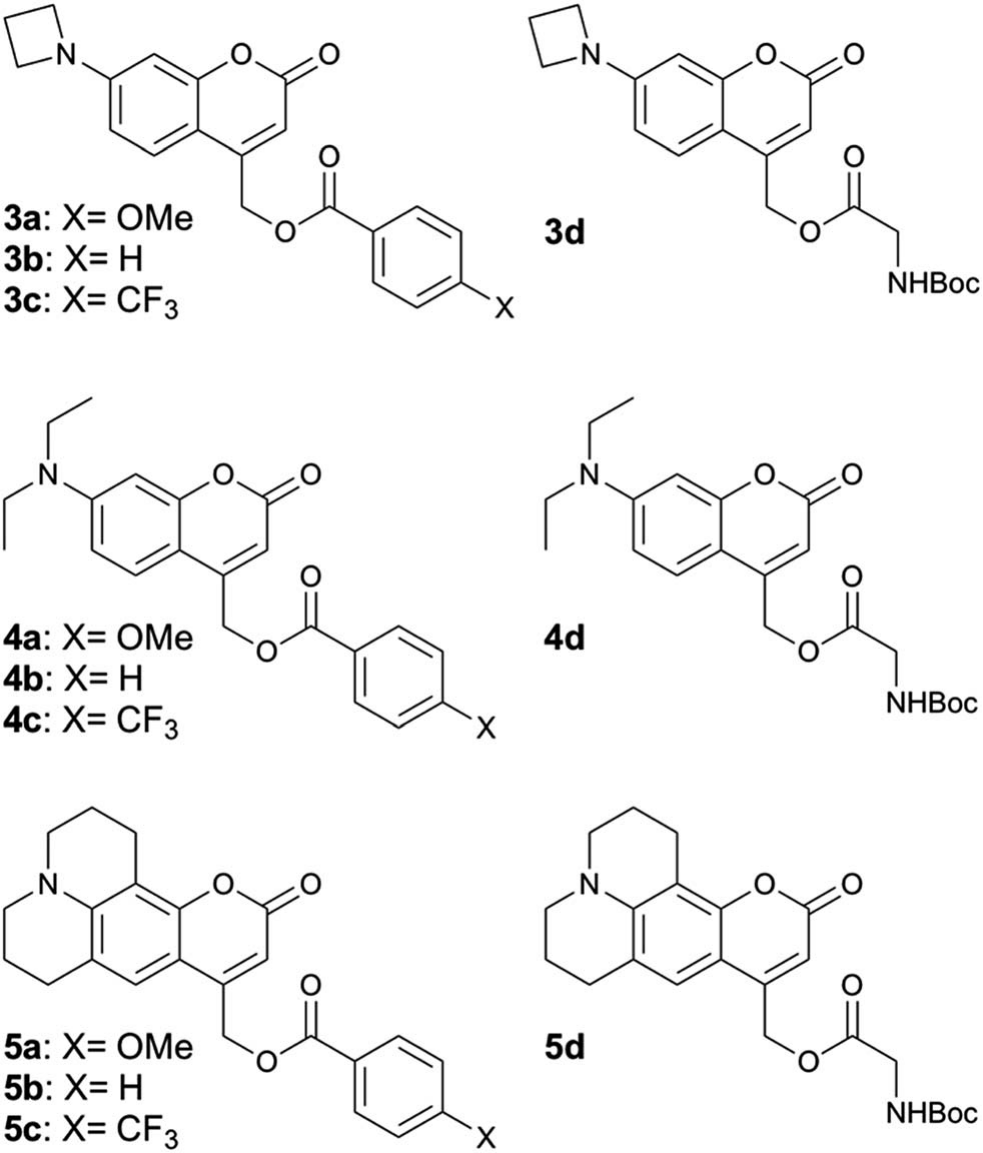
Structures of compounds **3a–d**, **4a–d** and **5a–d**. For details of the syntheses see the ESI.†

We synthesized 7-azetidinylcoumarin derivatives **3a–d**, 7-diethylaminocoumarin derivatives **4a–d** and the julolidine-fused derivatives **5a–d** (Chart 2). We prepared the julolidine series because for these compounds the involvement of the TICT state is prevented by incorporation of the nitrogen into a system of fused rings. The *φ*_PA_ values of compounds **3a–d**, **4a–d** and **5a–d** were measured in mixtures of phosphate-buffered saline (PBS, pH 7.4) and MeCN (3/7, v/v). The rates of photolysis of 0.3 mM solutions were determined *via* HPLC by following the disappearance of the starting material upon irradiation at 405 nm (7.5 mW, see ESI†).

**Fig. 1:**
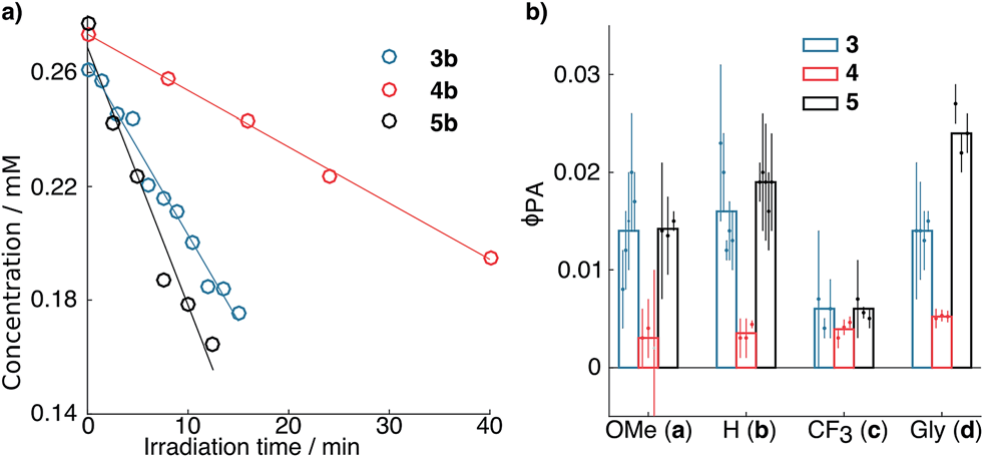
(a) Kinetics of disappearance of starting material upon irradiation as retrieved by HPLC for compounds **3b**, **4b** and **5b**. Open circles and solid lines represent the experimental points and the relative fits, respectively. (b) Quantum yields of photoactivation (*φ*_PA_) of the derivatives studied. The points represent individual measurements, and the associated error bars shown are obtained by propagating the 95% confidence interval on the disappearance rate retrieved from the fits (see ESI†). The bars on the background show the average value of the individual measurements plotted.

Fig. 1 depicts the results of photorelease experiments, including kinetic traces for representative runs of compounds **3b**, **4b** and **5b** (Fig. 1a) and a comparison of the measured *φ*_PA_ values of all the derivatives studied (Fig. 1b). In general, the *φ*_PA_ values of derivatives **3a–d** and **5a–d** are comparable and significantly larger than the ones measured for derivatives **4a–d**.

The most noticeable exceptions are compounds **3c**, **4c** and **5c**, which have overall lower *φ*_PA_, but even within this family, **3c** and **5c** seem to be photoreleased slightly more efficiently than **4c**. For all other compounds, the **4**- or **5**-fold increase in *φ*_PA_ observed is consistent with the increase observed between the *φ*_F_ of compounds **1** and **2**. These results confirm our hypothesis that azetidinyl substituents can be used to improve the efficiency of photochemical processes beyond fluorescence through inhibition of competitive deactivation pathways.

We then turned to investigate the nature of the decay channel inhibited by the azetidinyl substituent. Whereas it has been suggested that small-ring substituents inhibit the population of TICT states,^7^,^8^ the existence of TICT states for 7-substituted coumarins has never been firmly established.^9^ As a starting point, we performed femtosecond (fs) transient absorption (TA, 400 nm excitation and 100 fs time resolution, see ESI†) and fluorescence up-conversion spectroscopy measurements (FLUPS, 400 nm excitation, 1340 nm mixing pulse, 0.1 mm BBO, see ESI†) on the model fluorophores **1** and **2**, to look for spectral signatures of a TICT state. We chose to work on the fluorophores because the measured *φ*_F_ values (0.92 for **1***vs.* 0.06 for **2**) suggest that switching to the azetidinyl substituent renders radiative decay the predominant excited state process, whereas for the esters **3–5** heterolytic bond cleavage contributes to the kinetics of excited state decay, which may obscure the analysis.

**Fig. 2:**
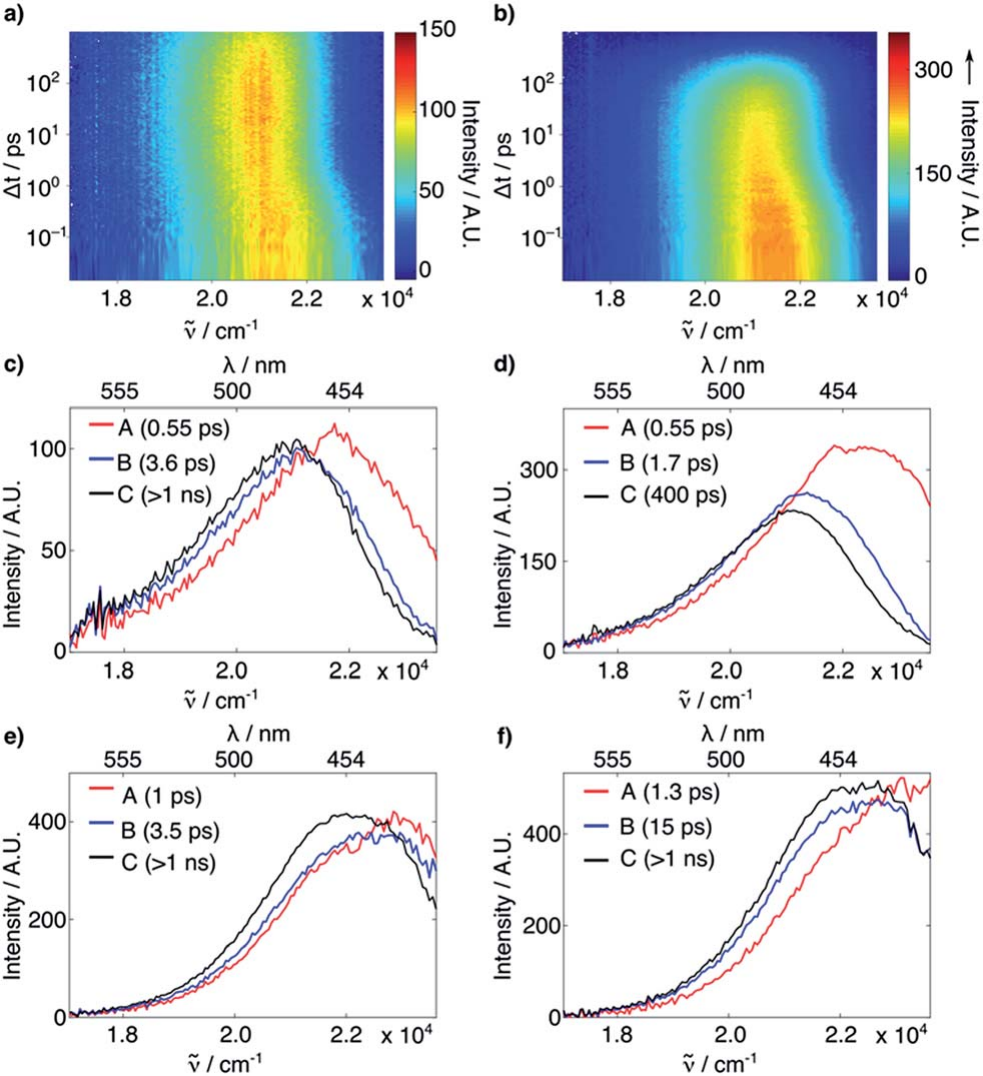
(a and b) Time-resolved fluorescence of **1** (a) and **2** (b) in H_2_O. (c–f) EAES obtained from a global target analysis of the time-resolved fluorescence of **1** in H_2_O (c) and DMSO (e) and of **2** in H_2_O (d) and DMSO (f), assuming a series of three successive exponential steps with the time constants indicated in the legends.

The FLUPS and TA data were analyzed globally assuming a series of exponential steps to obtain evolution associated emission or differential absorption spectra (EAES or EADS), respectively.^18^ FLUPS data in H_2_O and DMSO (Fig. 2) could be described for both dyes assuming three successive exponential steps. In H_2_O (Fig. 2a–d), a two-step red shift was observed, with a magnitude twice as large for **2** than for **1** (∼1200 and ∼200 cm^–1^*vs.* ∼680 and ∼100 cm^–1^, respectively). Furthermore, the longer time constant (reflecting the fluorescence lifetime, *τ*_F_, also determined from time-correlated single photon counting experiments, see ESI†), is more than one order of magnitude smaller for **2** than for **1** (0.40 ns *vs.* 5.0 ns). On the contrary, in DMSO (Fig. 2e–f) the red shifts (700 cm^–1^ for both compounds) and the *τ*_F_ (2.7 ns for **2***vs.* 3.3 ns for **1**) are comparable for the two dyes. The initial red shifts reflect the equilibration of the surrounding polar solvent molecules.^19^ The larger shifts observed with **2** in H_2_O point to substantial solvent reorganization and stabilization of the excited state, such as those associated with hydrogen-bond (H-bond) interactions.^20^ The shorter *τ*_F_ of **2** in H_2_O and MeOH (see ESI†), but not in other highly polar solvents such as MeCN and DMSO (see ESI†), does not support involvement of a TICT state. The TA data did not reveal either any spectral signature attributable to any state other than the optically-populated excited state (see ESI†). Therefore, other deactivation pathways must be operative.

**Fig. 3:**
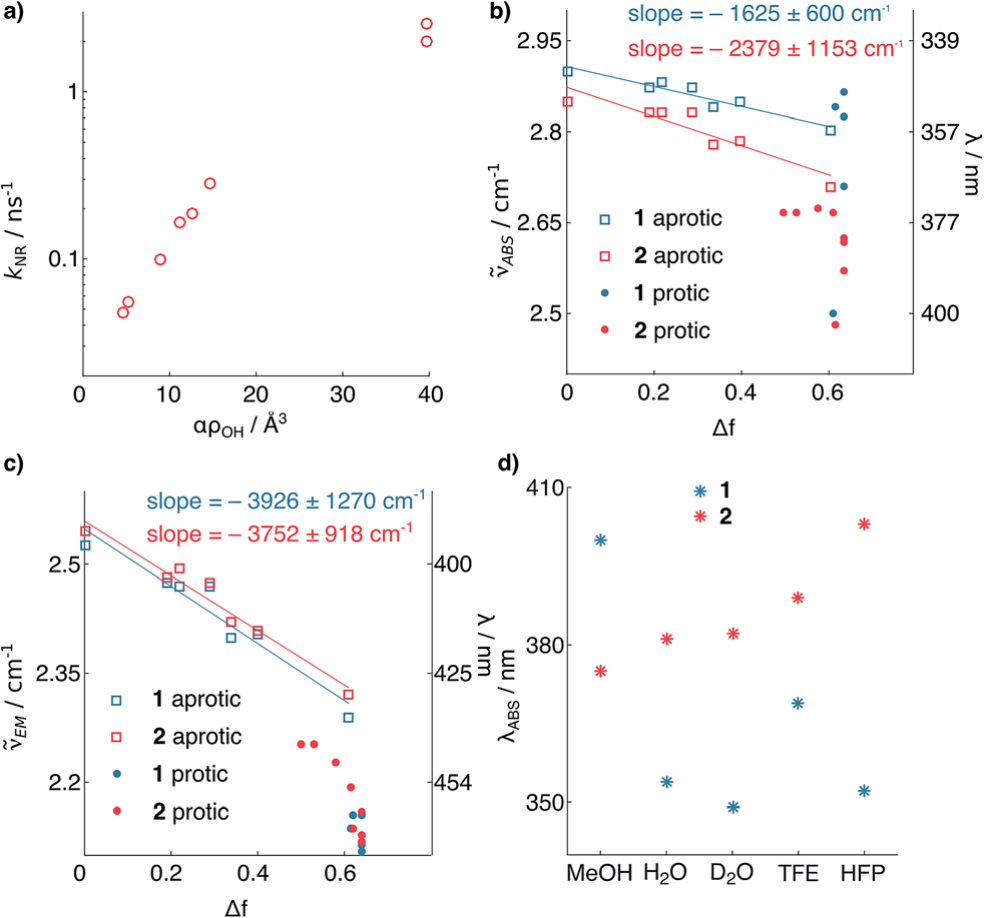
(a) Non-radiative decay rate constant of **2***vs.* solvent H-bonding ability. (b and c) Solvatochromism of **1** and **2**. Absorption (b) and emission (c) maximum wavelengths *vs.* the solvent polarity function.^25^ (d) Solvatochromism of absorption of **1** and **2** in protic solvents. The points represent individual measurements, and the associated errors of the slopes represent the 95% confidence interval of the fits (see ESI†). TFE = trifluoroethanol; HFP = hexafluoroisopropanol.

The shortening of *τ*_F_ for **2** in polar protic solvents strongly suggests the possibility of H-bond induced non-radiative decay (HBIND) as deactivation mechanism of the excited state, a phenomenon already reported for other dyes.^21^–^23^ The efficiency of this process was shown to depend on both the H-bond donating strength of the solvent, described by the Kamlet–Taft parameter *α*,^24^ and the ability of the solvent to make an H-bond network, quantified by the density of OH groups (*ρ*_OH_), itself approximated as 1/*V*_s_, where *V*_s_ is the volume of a solvent molecule. To test this hypothesis, we determined the non-radiative decay rates (*k*_nr_) of **1** and **2** from the *τ*_F_ and *φ*_F_ values in a series of solvents of different H-bonding abilities (Fig. 3a, see ESI†). The correlation observed between *k*_nr_ and *αρ*_OH_ supports the involvement of HBIND as deactivation channel for **2** in protic solvents, but does not explain why the same pathway is not active for **1**, which is structurally very similar.

To shed light on the origin of these differences, we analyzed the solvatochromism of the absorption and emission bands of **1** and **2** in solvents of different polarities, both protic and aprotic (Fig. 3b and c, see ESI†). From these measurements it is possible to estimate the ground (*μ*_g_) and excited (*μ*_e_) state electric dipoles of the two dyes (see ESI†).^25^,^26^ We observed that the dipole moments of **1** (*μ*_g_ = 3.9, *μ*_e_ = 9.5 D) are significantly smaller than for **2** (*μ*_g_ = 7.5, *μ*_e_ = 11.8 D). Analysis of the solvatochromism of the absorption bands reveals further details. For **2** in protic solvents, a consistent bathochromic shift is observed (Fig. 3d), whereas for **1** both batho- and hypso-chromic shifts are found. These shifts can be rationalized considering the internal charge transfer (ICT) character of the first singlet excited state of coumarins, in which the substituent at C7 donates electron density to the carbonyl group at C2.^25^,^27^ H-bonding to the nitrogen atom stabilizes more the ground than the excited state resulting in a blue shifted absorption, whereas H-bonding to the carbonyl group has the opposite effect. The observed shifts of the absorption maximum (Fig. 3d) suggest that for dye **1**, H-bonding is happening at both ends of the molecule; by contrast, in the case of compound **2**, H-bonding seems to involve mainly the carbonyl moiety. This observation suggests that in both states the carbonyl of **2** has higher electron density than in **1**, and is thus more prone to H-bonding. The smaller dipole moments of **1** relative to **2** point to weaker electron-donating strength of the azetidinyl substituent. This observation is in agreement with the higher ionization potential of phenylazetidine (7.61 eV)^28^ compared to that of *N*,*N*-diethylaniline (6.95 eV).^29^

To confirm that this mechanism also applies to the photolabile esters, we measured the *φ*_PA_ of compounds **3a–5a** using *n*-pentanol (PeOH) and EtOH as co-solvents (see ESI†) instead of PBS because both display lower H-bonding ability than H_2_O. In EtOH mixture (for PeOH results, see ESI†), the *φ*_PA_ of **3a**, **4a** and **5a** became very similar (*φ*_PA_ = 0.007, 0.0037, and 0.01, respectively). These efficiencies are in general lower than in aqueous mixture, which can be attributed to the reduced stabilization (and hence formation) of the ion pair in less polar solvents. These observations further support our working hypothesis that azetidinyl substituents shut down HBIND-associated decay of coumarin excited states, leading to increased fluorescence or photoactivation efficiency in H_2_O.

**Fig. 4:**
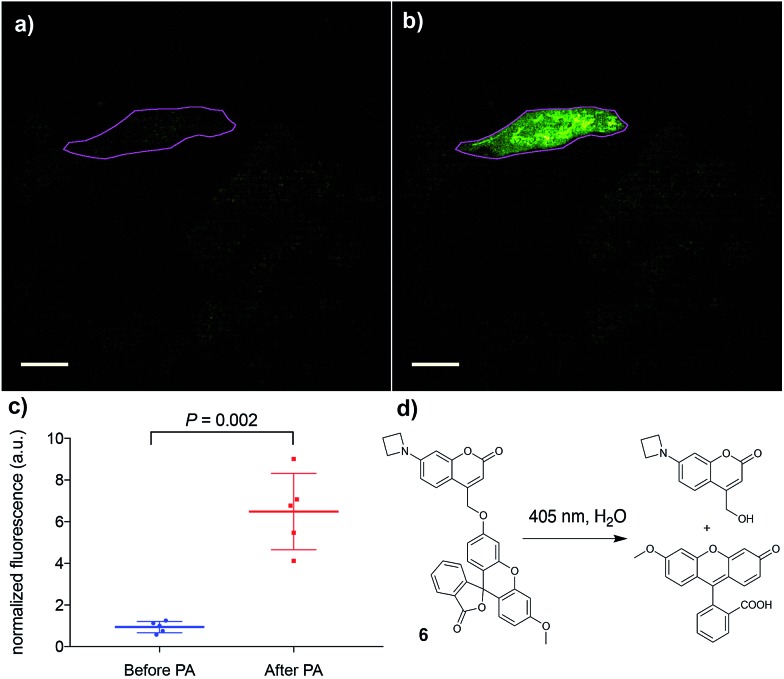
Photoactivation (PA) of compound **6**. (a) Image of live HeLa cells before photoactivation. The magenta outline denotes the region of interest to be irradiated. (b) Image of the same cells as in (a) after irradiation of the region of interest. (c) Quantification of the increase in intracellular fluorescence upon photoactivation of **6**; mean and standard deviation are shown along with the values of five independent measurements. (d) Photoactivation reaction of compound **6** to release the fluorophore methylfluorescein. Scale bar = 20 μM.

Finally, the applicability of this improved photoremovable group was confirmed in live cells. We chose to prepare compound **6** (Fig. 4, for synthesis see ESI†), which is a photoactivatable fluorescein probe. Photoactivatable dyes are useful for a number of applications including cell tracking,^30^ intracellular diffusion experiments^31^ and super-resolution microscopy.^32^ Live human cervical cancer (HeLa) cells were incubated with compound **6** in growth medium for 30 min. After this time, imaging in the green channel (*λ*_ex_ = 488 nm; *λ*_em_ = 525 ± 25 nm) gave nearly no signal, confirming that the fluorescein fragment of compound **6** remains in the dark, spirolactone form (Fig. 4) and the coumarin is not excited at that wavelength. A region of interest comprising a single cell (Fig. 4) was irradiated with a 405 nm laser (120 mW, 50% power), which induced a 7-fold increase in fluorescence intensity. This experiment confirms that the azetidinylcoumarin protecting group can be removed with light in live cells.

## Conclusions

To the best of our knowledge, azetidinyl substituents have been used, in the context of photochemistry, only to increase the *φ*_F_ of fluorophores.^7^,^8^ We have demonstrated, however, that this simple substitution leads to the enhancement of other useful photoprocesses in the presence of H_2_O. We also propose a mechanism of quenching that does not invoke TICT states, but rather HBIND as the unproductive decay channel that is shut down by azetidinyl donors in coumarins. Albeit beyond the scope of this work, we propose that azetidinyl substitution could enhance other important photochemical processes in H_2_O; for instance, would an azetidinylated version of methylene blue be more efficient in photocatalysis^33^ or photodynamic therapy?^34^ Further studies are required to investigate the origin of the increase in *φ*_F_ observed for other fluorophores and determine whether azetidinyl substitution affects the population of TICT states, as currently thought, or it deactivates other excited state decay pathways as it seems to be happening for the coumarins reported here. Testing these hypotheses might reserve interesting challenges and surprises.

## Conflicts of interest

The authors declare no competing financial interests.

## Supplementary Material

Supplementary information
