# Supplementary material for: Photolabile coumarins with improved efficiency through azetidinyl substitution
Source: Chem Sci. 2017 Oct 31;9(2):387–91. doi: 10.1039/c7sc03627b (PMC5868312; doi:10.1039/c7sc03627b)
Supplement: Supplementary file 1 [file SC-009-C7SC03627B-s001.pdf]

Electronic Supplementary Material (ESI) for Chemical Science.
This journal is © The Royal Society of Chemistry 2017

Photolabile Coumarins with Improved Efficiency through Azetidinyl Substitution

Giovanni Bassolino,[a] Christoph Nançoz,[b] Zacharias Thiel,[a] Estelle Bois,[b] Eric Vauthey,[b*] and Pablo Rivera-Fuentes[a*]

Supporting Information

Contents

General methods 2

Synthesis and characterization of compounds 4

Photochemical Characterization of the Esters **3-5a-d** 14

Photochemical Characterization of the Fluorophores **1** and **2** 18

Dipole Moment Determination 23

References 25

NMR Spectra 27

a Laboratorium für Organische Chemie, ETH Zürich,
HCI G329, Vladimir-Prelog-Weg 3, 8093 Zürich, Switzerland.
E-mail: pablo.rivera-fuentes@org.chem.ethz.ch

b Laboratory of Physical Chemistry, University of Geneva,
30 Quai Ernest-Ansermet, 1211 Geneva 4, Switzerland.
E-mail: Eric.Vauthey@unige.ch

General methods

All reagents were purchased from commercial sources and used as received. 7-(Dimethylamino)-4-methyl-2*H*-chromen-2-one (**2**) was purchased from Tokyo Chemical Industry (cat. N. D3355-5G) and used as received. Dry solvents were procured from Acros Organics and used as received. For preparative column chromatography, petroleum ether (Pet. Et.) 40–60 °C was used in place of hexane. NMR spectra were acquired on Bruker AV300 or Bruker 400 instruments. ^{1}H NMR chemical shifts are reported in ppm relative to $SiMe_4$ (δ = 0) and were referenced internally with respect to residual protons in the solvent (δ = 2.50 for $(CD_3)_2SO$, δ = 7.26 for $CDCl_3$). Coupling constants are reported in Hz and multiplicities of signals are described as singlet (s), doublet (d), triplet (t), quartet (q), quintuplet (quint), or multiplet (m). ^{13}C NMR chemical shifts are reported in ppm relative to $SiMe_4$ (δ = 0) and were referenced internally with respect to solvent signal (δ = 39.5 for $(CD_3)_2SO$, δ = 77.2 for $CDCl_3$). Peak assignments are based on calculated chemical shift, multiplicity, and 2D experiments. UV-visible spectra were obtained employing a Cary 500 Scan spectrometer using quartz cuvettes from ThorLabs (10 mm path length).

Fluorescence spectra were recorded on a Cary Eclipse fluorometer. All emission spectra were corrected for the wavelength dependence sensitivity of the detector. The fluorescence quantum yields were determined relatively to perylene (ϕ_F = 0.92 in ethanol).[1] High-resolution mass spectrometry was conducted by staff at the Molecular and Biomolecular Analysis (MoBiAs) center (ETH Zurich) employing a Bruker maXIS ESI/NanoSpray-Qq-TOF-MS or a Bruker solariX ESI/MALDI-FTICR-MS instrument. LC/MS were recorded on a Waters Acquity UPLC system, equipped with a Waters Acquity BEH C18 column (5 cm length, 2.1 mm internal diameter), a diode array detector PDA and an SQ Detector 2 ZSPRAY (ESI). Analytical HPLC was carried out using a Hitachi instrument equipped with an autosampler and a diode array detector (DAD) and employing an ACE Excel 5 C18-Amide column (25 cm length, 4.6 mm internal diameter). Gradient 1: Solvent A: H_2O, Solvent B: MeCN (flow 1 mL min^{-1}, 0–1 min: 50% A; 1–3 min: 50–20 % A; 3–6.5 min: 20% A; 6.5–7.5 min: 20–10 % A; 7.5–9.5 min: 10% A; 9.5–11 min: 10–50% A; 11–13 min: 50% A). Gradient 2: MeOH kept constant at 45% for the entire run; Solvent A: H_2O, Solvent B: MeCN (flow 1 mL min^{-1}, 0-1 min: 50% A; 1-3 min: 50-20% A; 3-6.5 min: 20% A; 6.5-9 min: 20-10% A; 9-9.5 min: 10% A; 9.5-11 min: 10-50% A; 11-13 min: 50% A).

IUPAC names of all compounds are provided and were determined using CS ChemDraw Professional 15.0.

Fluorescence up-conversion spectroscopy (FLUPS) measurements were carried out with a setup similar to that developed by Ernsting and coworkers.[2] In brief, excitation was performed at 400 nm, whereas up-conversion was carried out by mixing the fluorescence with a 1340 nm pulse in a 0.1 mm BBO crystal. The full width at half maximum of the instrumental response function (IRF) was 175 fs. The transient spectra were recorded from 420 to 600 nm and up to a time delay of 1 ns. FLUPS measurements were performed for both **1** and **2** in H_2O, MeOH, MeCN and DMSO. Transient absorption (TA) measurements were performed with two setups, one with 400 nm excitation, 100 fs time resolution and 0-1.5 ns time window,[3] and the other with 355 nm excitation, 300 ps time resolution and 0-1 ms time window.[4] FLUPS and TA data were analyzed globally using a sum of exponential functions or assuming a series of consecutive exponential steps with increasing time constants. The latter approach allows the relevant time scale to be obtained and yields so-called evolution-associated emission spectra (EAES) for FLUPS or evolution-associated differential absorption spectra (EADS) for TA.[5] The global target analysis was performed using a MATLAB (MathWorks) procedure with a combination of an initial value ordinary differential equation solver and the matrix reconstruction algorithm.[6] The optimization was performed iteratively with the Nedler-Mead algorithm. The first 100-200 fs TA and FLUPS data were not included in the analysis because of the contribution from the cross-phase modulation (TA) and Raman scattering (FLUPS) to the spectra around time zero.

Fluorescence lifetime measurements were carried using a time-correlated single photon counting setup with 395 nm excitation and 200 ps IRF.[7] The fluorescence lifetimes were obtained by iterative reconvolution of the IRF with an exponential function.

Cell culture and fluorescence imaging

HeLa cells (ATCC® CCL-2™) were grown in Dulbecco's Modified Eagle Medium (DMEM) supplemented with fetal bovine serum (FBS, 15%) and penicillin-streptomycin (1%) at 37 ºC in a 95% humidity atmosphere under 5% CO_2

environment. The cells were grown to 90% confluence before seeding at a density of 50 000 cells mL^{-1} onto a Nunc™ Lab-Tek™ II chambered coverglass 48 h before the experiment. Prior to imaging, the growth medium was removed and the cells were rinsed with PBS (0.5 mL). A 10 μM solution of **6** in PBS was added to the well and the cells were incubated for 30 min at 37 °C. The medium was removed, the cells were rinsed twice with PBS, FluoroBrite™ DMEM (Thermo Fisher, 0.5 mL) was added, and the cells imaged. Fluorescence images of cells were collected using a Nikon Eclipse T1 microscope equipped with a Yokogawa spinning-disk confocal scanner unit CSU-W1-T2, two sCMOS cameras (Orca Flash 4.0 V2) and a LUDL BioPrecision2 stage with piezo focus. The light sources were diode-pumped, solid-state lasers (DPSS): 405 nm (blue channel, 120 mW, 50% power), which was used for all photoactivation experiments (exposure time: 2 ms $pixel^{-1}$), 488 nm (green channel, 200 mW, 20%), which was used for imaging the photoreleased fluorescein. Emission in the green channel was filtered with a 525/50 bandpass filter. Fluorescence images were obtained using an oil-immersion objective with a magnification of 100× 1.49 CFI Apo TIRF. The microscope was operated using VisiVIEW (Metamorph) software. The exposure time for acquisitions (400 ms) was kept constant for all images. Quantification of fluorescence intensity was performed using FIJI (ImageJ 1.51d, NIH). Fluorescence intensity was quantified by defining regions of interest (ROIs) comprising the whole cell body and recording the integrated intensity within this region. Differences in fluorescence intensity were calculated directly from the intensities in the ROI before and after photoactivation. Data sets were analyzed by unpaired, two-tailed, Student's t-test. Results are presented as medians, error bars represent standard deviation, and numerical *p* values are provided. Statistical analyses were carried out using Prism 6 (GraphPad).

Synthesis and characterization of compounds

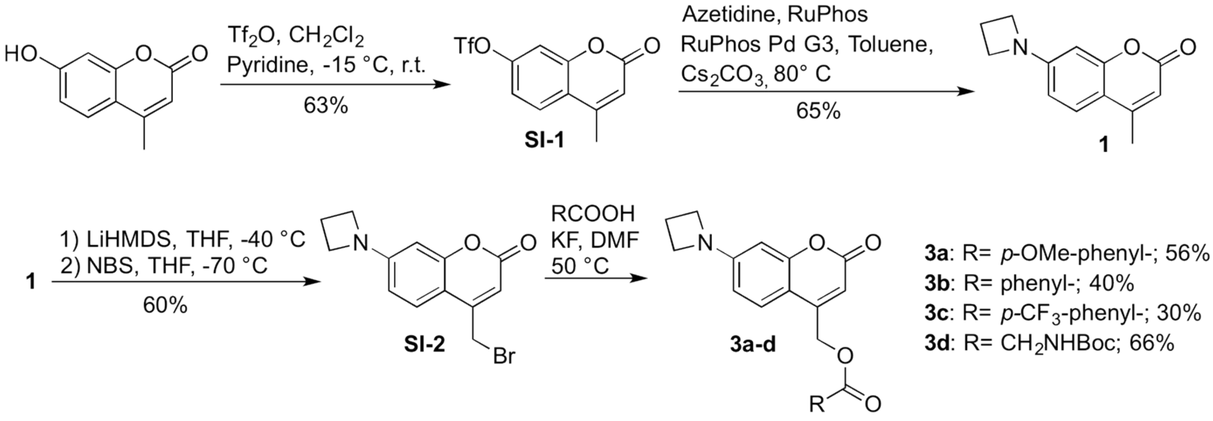

Scheme S1. Synthesis of compounds **3a-d**.

4-Methyl-2-oxo-2*H*-chromen-7-yl trifluoromethanesulfonate (SI-1)

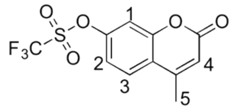

7-Hydroxy-4-methylcoumarin (2 g, 11.3 mmol) was added to a flame-dried flask, which was evacuated and backfilled with nitrogen three times. The solid was suspended in dry CH_2Cl_2 (28 mL), and dry pyridine (1.83 mL, 22.6 mmol) was added. The suspension was cooled to –15 °C in an ice/NaCl bath. A solution of trifluoromethanesulfonic anhydride (3.83 g, 13.5 mmol, 2.3 mL) in dry CH_2Cl_2 (6 mL) was added dropwise. The mixture was stirred for 30 min at –15 °C, then the bath was removed and the reaction was stirred for further 30 min at room temperature. The solution was washed twice with 0.1 M HCl, followed by H_2O and brine. The organic phase was dried over $MgSO_4$ and the solvent was removed under reduced pressure. The product was purified by column chromatography (SiO_2, Pet. Et./EtOAc, 85/15 → 7/3 (v/v)) to yield 2.2 g (63% yield) of **SI-1** as an off-white solid. ^{1}H NMR (400 MHz, $CDCl_3$): δ = 2.46 (d, *J* = 1.3 Hz, 3H, H5), 6.36 (d, *J* = 1.3 Hz, 1H, H4), 7.24 (dd, *J* = 8.7, 2.5 Hz, 1H, H2), 7.28 (d, *J* = 2.4 Hz, 1H, H1), 7.70 (d, *J* = 8.7 Hz, 1H, H3). ^{13}C NMR (100 MHz, $CDCl_3$): δ = 18.8, 110.5, 116.0, 117.4, 118.7 (q, *J* = 321 Hz), 120.1, 126.3, 150.8, 151.2, 154.1, 159.4. HRMS (ESI) calcd for $[C_{11}H_{18}F_3O_5S_2]^+$: 309.0039, found 309.0041.

7-(Azetidin-1-yl)-4-methyl-2*H*-chromen-2-one (1)[8]

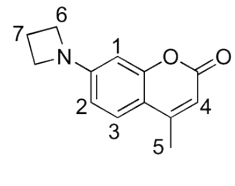

Compound **SI-1** (800 mg, 2.6 mmol), RuPhos (182 mg, 0.4 mmol), RuPhos Pd G3 (334 mg, 0.4 mmol) and Cs_2CO_3 (1.27 g, 3.9 mmol) were transferred to a flame-dried Schlenk flask, which was evacuated and backfilled with nitrogen three times. Dry

toluene (21 mL) was added, followed by azetidine (162 mg, 2.8 mmol, 0.2 mL). The reaction was stirred for 15 h at 80 °C, then filtered over Celite, fixed on SiO_2 and purified by column chromatography (SiO_2, CH_2Cl_2/EtOAc, 98/2 (v/v)) to yield compound **1** as a bright yellow solid (360 mg, 65% yield). ^{1}H NMR (400 MHz, $CDCl_3$): δ = 2.33 (d, *J* = 1.2 Hz, 3H, H5), 2.42 (quint, *J* = 7.3 Hz, 2H, H7), 3.98 (t, *J* = 7.3 Hz, 4H, H6), 5.95 (q, *J* = 1.2 Hz, 1H, H4), 6.20 (d, *J* = 2.3 Hz, 1H, H1), 6.28 (dd, *J* = 8.6, 2.3 Hz, 1H, H2), 7.36 (d, *J* = 8.6 Hz, 1H, H3). ^{13}C NMR (100 MHz, $CDCl_3$): δ = 16.6, 18.7, 51.9 97.2, 107.8, 109.5, 110.4, 125.5, 153.1, 154.0, 155.7, 162.0. HRMS (ESI) calcd for $[C_{13}H_{14}N_1O_2]^+$: 216.1019, found 216.1016.

7-(Azetidin-1-yl)-4-(bromomethyl)-2*H*-chromen-2-one (SI-2)

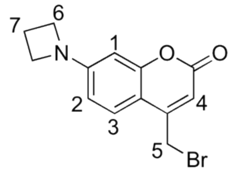

Compound **1** (385 mg, 1.79 mmol) was added to a flame-dried flask and suspended in dry THF (39 mL). The suspension was cooled to –40 °C in a MeCN/dry ice bath and LiHMDS (4.47 mmol, 1 M in THF, 4.47 mL) was added to the mixture. The solution was warmed to –30 °C, followed by cooling to –78 °C in a dry ice/acetone bath. A solution of *N*-bromosuccinimide (350 mg, 1.97 mmol) in dry THF (7 mL) was added and the solution was stirred at –78 °C. The reaction was neutralized after 1 h, when LC/MS and TLC analyses revealed the presence of a di-brominated byproduct (*m*/*z* = 374). The mixture was treated with HCl (0.1 M) and repeatedly extracted with CH_2Cl_2. The combined organic phases were dried over $MgSO_4$ and the solvent was removed under reduced pressure. The product was purified by column chromatography (SiO_2, CH_2Cl_2/EtOAc, 99/1 → 97/3 (v/v)) to yield an orange solid (310 mg, 60% yield). ^{1}H NMR (400 MHz, $CDCl_3$): δ = 2.44 (quint, *J* = 7.4 Hz, 2H, H7), 4.01 (t, *J* = 7.3 Hz, 4H, H6), 4.40 (s, 2H, H5), 6.17 (s, 1H, H4), 6.22 (d, *J* = 2.3 Hz, 1H, H1), 6.33 (dd, *J* = 8.7, 2.3 Hz, 1H, H2), 7.48 (d, *J* = 8.7 Hz, 1H, H3). ^{13}C NMR (100 MHz, $CDCl_3$): δ = 16.6, 27.3, 51.8, 97.3, 107.4, 108.0, 110.1, 125.4, 150.6, 154.1, 156.4, 161.6. HRMS (ESI) calcd for $[C_{13}H_{13}Br_1N_1O_2]^+$: 294.0124, found 294.0128.

General procedure for the formation of 7-azetidin-1-yl-coumarin 4-methyl ester derivatives (3a-d) and compound 6.[9]

Potassium fluoride (47 mg, 0.81 mmol) was added to a flame-dried flask and further flame-dried under high vacuum three times. Compound **SI-2** (80 mg, 0.27 mmol) and the acid to be coupled (0.3 mmol) were added, and the system was evacuated and backfilled with nitrogen three times. For the synthesis of compound **6**, **SI-2** (40 mg, 0.135 mmol) and methylfluorescein[10] (0.15 mmol) were used. The flask was wrapped in aluminum foil before adding dry DMF (5 mL). The reaction was stirred at 45 °C for 2-3 h. The solution was diluted with CH_2Cl_2 and the organic phase was washed twice with H_2O. The organic phase was dried over $MgSO_4$ and the solvent was removed under reduced pressure. Details of the purification procedure followed are given for each compound.

(7-(Azetidin-1-yl)-2-oxo-2*H*-chromen-4-yl)methyl 4-methoxybenzoate (3a)

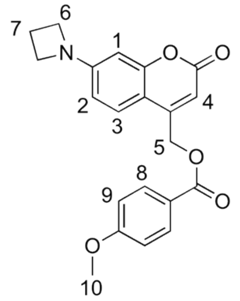

The reaction mixture was separated via flash chromatography (SiO_2, CH_2Cl_2/EtOAc, 100/0 → 95/5 (v/v)). Isolated amount: 55 mg, 56% yield. ^1H NMR (400 MHz, $CDCl_3$): δ = 2.45 (quint, *J* = 7.4 Hz, 2H, H7), 3.88 (s, 3H, H10), 4.01 (t, *J* = 7.4 Hz, 4H, H6), 5.44 (d, *J* = 1.4 Hz, 2H, H5), 6.24 (d, *J* = 2.3 Hz, 1H, H1), 6.28 (t, *J* = 1.4 Hz, 1H, H4), 6.31 (dd, *J* = 8.7, 2.3 Hz, 1H, H2), 6.92–6.97 (m, 2H, H9), 7.36 (d, *J* = 8.7 Hz, 1H, H3), 8.03–8.09 (m, 2H, H8). ^{13}C NMR (100 MHz, $CDCl_3$): δ = 16.6, 51.8, 55.7, 61.7, 97.3, 107.2, 107.4, 108.1, 114.0, 121.7, 124.4, 132.1, 150.1, 154.0, 156.0, 161.8, 164.0, 165.6. HRMS (ESI) calcd for $[C_{21}H_{20}N_1O_5]^+$: 366.1336, found 336.1342.

(7-(Azetidin-1-yl)-2-oxo-2*H*-chromen-4-yl)methyl benzoate (3b)

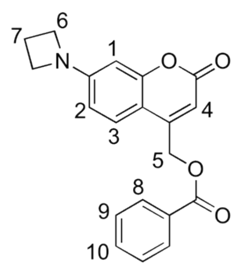

The reaction mixture was separated via flash chromatography (SiO_2, CH_2Cl_2/EtOAc, 100/0 → 95/5 (v/v)). Isolated amount: 40 mg, 40% yield. ^1H NMR (400 MHz, $CDCl_3$): δ = 2.45 (quint, *J* = 7.4 Hz, 2H, H7), 4.01 (t, *J* = 7.4 Hz, 4H, H6), 5.47 (d, *J* = 1.4 Hz, 2H, H5), 6.25 (d, *J* = 2.3 Hz, 1H, H1), 6.29 (t, *J* = 1.4 Hz, 1H, H4), 6.32 (dd, *J* = 8.7, 2.3 Hz, 1H, H2), 7.37 (d, *J* = 8.7 Hz, 1H, H3), 7.45–7.51 (m, 2H, H9), 7.59–7.64 (m, 1H, H10), 8.08–8.13 (m, 2H, H8). ^{13}C NMR (100 MHz, $CDCl_3$): δ = 16.6, 51.8, 62.0, 97.3, 107.3, 107.3, 108.1, 124.4, 128.8, 129.3, 130.0,

133.8, 149.8, 154.1, 156.0, 161.8, 165.9. HRMS (ESI) calcd for $[C_{20}H_{18}N_1O_4]^+$: 336.1230, found 336.1236.

(7-(Azetidin-1-yl)-2-oxo-2*H*-chromen-4-yl)methyl 4-(trifluoromethyl)benzoate (3c)

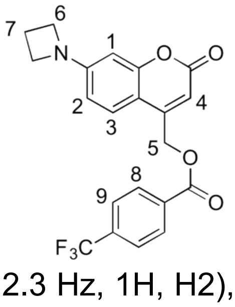

Purified by preparative thin layer chromatography (SiO_2, CH_2Cl_2/EtOAc, 96/4 (v/v)), followed by recrystallization from MeOH. Isolated amount: 25 mg, 30%. ^{1}H NMR (400 MHz, $CDCl_3$): δ = 2.46 (p, *J* = 7.4 Hz, 2H, H7), 4.02 (t, *J* = 7.4 Hz, 4H, H6), 5.49 (d, *J* = 1.3 Hz, 2H, H5), 6.25 (m, 2H, H1 and H4), 6.32 (dd, *J* = 8.7, 2.3 Hz, 1H, H2), 7.36, (d, *J* = 8.7 Hz, 1H, H3), 7.61–7.88 (m, 2H, H9), 8.20–8.25 (m, 2H, H8). ^{13}C NMR (100 MHz, $CDCl_3$): δ = 16.6, 51.8, 62.5, 97.4, 107.2, 107.4, 108.1, 123.6 (q, *J* = 272.8 Hz), 124.4, 125.8 (q, *J* = 3.7 Hz), 130.4, 132.5, 135.2 (q, *J* = 32.8 Hz), 149.2, 154.1, 156.1, 161.7, 164.8. HRMS (ESI) calcd for $[C_{21}H_{17}F_3N_1O_4]^+$: 404.1104, found 404.1109.

(7-(Azetidin-1-yl)-2-oxo-2*H*-chromen-4-yl)methyl pivaloylglycinate (3d)

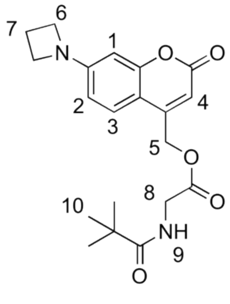

Purified by preparative thin layer chromatography (SiO_2, CH_2Cl_2/EtOAc, 8/2 (v/v)). Isolated amount: 65 mg, 66%. ^{1}H NMR (400 MHz, $CDCl_3$): δ = 1.46 (s, 9H, H10), 2.45 (q, *J* = 7.3 Hz, 2H, H7), 4.00 (t, *J* = 7.3 Hz, 4H, H6), 4.03 (d, *J* = 6 Hz, 2H, H8), 5.02 (tbr, *J* = 6 Hz, 1H, H9), 5.27 (d, *J* = 1.2 Hz, 2H, H5), 6.14 (t, *J* = 1.2 Hz, 1H, H4), 6.22 (d, *J* = 2.2 Hz, 1H, H1), 6.29 (dd, *J* = 8.7, 2.2 Hz, 1H, H2), 7.26 (d, *J* = 8.7 Hz, 1H, H3). ^{13}C NMR (100 MHz, $CDCl_3$): δ = 16.4, 28.3, 42.4, 51.7, 62.1, 80.4, 97.2, 107.0, 107.4, 107.9, 124.2, 148.8, 153.9, 155.7, 155.9, 161.4, 169.8. HRMS (ESI) calcd for $[C_{20}H_{24}N_2Na_1O_6]^+$: 411.1527, found 411.1531.

3'-((7-(Azetidin-1-yl)-2-oxo-2*H*-chromen-4-yl)methoxy)-6'-methoxy-3*H*-spiro[isobenzofuran-1,9'-xanthen]-3-one (6)

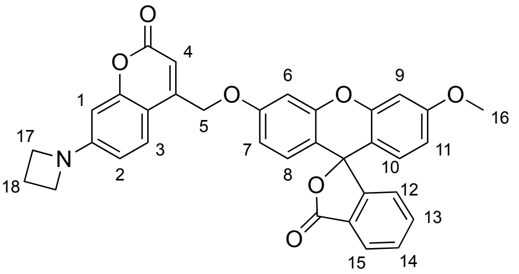

The reaction mixture was separated via preparative thin layer chromatography (SiO_2, CH_2Cl_2/MeOH, 95/5 (v/v)). Isolated amount: 28 mg, 37% yield. ^{1}H NMR (300 MHz, $CDCl_3$): δ =

2.49 (p, *J* = 7.4 Hz, 2H, H18), 3.94 (s, 3H, H16), 3.98–4.13 (m, 4H, H17), 4.79 (d, *J* = 12.7 Hz, 1H, H5a), 5.05 (d, *J* = 12.7 Hz, 1H, H5b), 5.48 (s, 1H, H4), 5.96 (dd, *J* = 8.7, 2.3 Hz, 1H, H2), 6.07 (d, *J* = 1.9 Hz, 1H, H6), 6.17 (d, *J* = 2.2 Hz, 1H, H1), 6.43 (dd, *J* = 9.7, 1.9 Hz, 1H, H7), 6.59–6.91 (m, 5H, H3, H8, H9, H10, H11), 7.07–7.32 (m, 2H, H12), 7.73 (pd, *J* = 7.5, 1.6 Hz, 2H, H13, H14), 8.34 (dd, *J* = 7.6, 1.7 Hz, 1H, H15). ^{13}C NMR (100 MHz, $CDCl_3$): δ = 16.43, 51.54, 56.09, 63.18, 96.56, 99.92, 105.85, 106.77, 107.91, 108.61, 114.03, 114.36, 117.25, 124.26, 128.40, 129.40, 129.82, 129.89, 129.96, 130.89, 131.84, 133.32, 134.34, 147.19, 149.42, 153.98, 154.05, 155.99, 158.31, 161.38, 164.31, 164.96, 185.21. HRMS (ESI) calcd for $[C_{34}H_{26}NO_7]^+$: 560.1704, found 560.1706

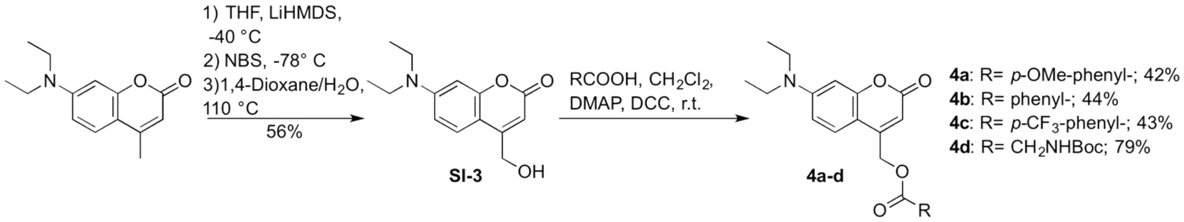

Scheme S2. Synthesis of compounds **4a-d**.

7-(Diethylamino)-4-(hydroxymethyl)-2*H*-chromen-2-one (SI-3)

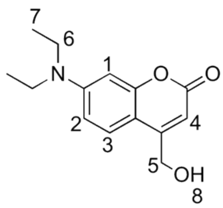

7-Diethylamino-4-methylcoumarin (500 mg, 2.16 mmol,) was dissolved in dry THF (24 mL) in a flame-dried flask. The solution was cooled to –40 °C in a MeCN/dry ice bath and LiHMDS (4.8 mmol, 1 M in THF, 4.8 mL) was added to the mixture. The solution was warmed to –30 °C, followed by cooling to –78 °C in a dry ice/acetone bath. A solution of *N*-bromosuccinimide (461 mg, 2.6 mmol) in dry THF (7 mL) was added, and the mixture was stirred at –78 °C for 1 h, before quenching with HCl (1 M).[11–16] The solution was repeatedly extracted with CH_2Cl_2. The combined organic phases were dried over $MgSO_4$ and the solvent was removed under reduced pressure. The crude was dissolved in 1,4-dioxane (30 mL) and H_2O (30 mL) and heated to reflux for 24 h. The resulting suspension was cooled to room temperature and extracted three times with EtOAc. The combined organic phases were dried over $MgSO_4$ and the solvent was removed under reduced pressure. The crude was purified by column chromatography (SiO_2, Pet. Et./EtOAc, 6/4 (v/v)) to yield compound **SI-3** as an orange powder (300 mg, 56% yield). ^1H NMR (400 MHz, $(CD_3)_2SO$): δ = 1.11 (t, *J* = 7.0 Hz, 6H, H7), 3.42 (q, *J* = 7.0 Hz, 4H, H6), 4.48–4.87

(m, 2H, H5), 5.48 (t, *J* = 4.0 Hz, 1H, H8), 6.06 (t, *J* = 1.2 Hz, 1H, H4), 6.52 (d, *J* = 2.6 Hz, 1H, H1), 6.66 (dd, *J* = 9.0, 2.6 Hz, 1H, H2), 7.43 (d, *J* = 9.0 Hz, 1H, H3). ^{13}C NMR (100 MHz, $(CD_3)_2SO$): δ = 12.3, 43.9, 59.0, 96.8, 103.9, 105.7, 108.5, 125.1, 150.2, 155.6, 156.8, 161.1. HRMS (ESI) calcd for $[C_{14}H_{18}N_1O_3]^+$: 248.1281, found 248.1282.

General procedure for the formation of 7-diethylamino-4-methyl ester derivatives (4a-d)[17]

Compound **SI-3** (70 mg, 0.28 mmol), the corresponding acid (0.34 mmol) and *N,N*-dimethylaminopyridine (34 mg, 0.28 mmol) were mixed in a flame-dried flask covered with aluminum foil, and evacuated and backfilled with nitrogen three times. Dry CH_2Cl_2 (1 mL) was added and cooled to 0 °C in an ice bath. *N,N'*-Dicyclohexylcarbodiimide (58 mg, 0.28 mmol) was dissolved in dry CH_2Cl_2 (0.75 mL) and added to the reaction flask. After 10 min, the ice bath was removed and the reaction was stirred for 12 h. The mixture was extracted with 1.2 M HCl and a saturated aqueous solution of $NaHCO_3$, dried over $MgSO_4$ and evaporated under reduced pressure. Details of the purification procedure followed are given for each compound.

(7-(Diethylamino)-2-oxo-2*H*-chromen-4-yl)methyl 4-methoxybenzoate (4a)

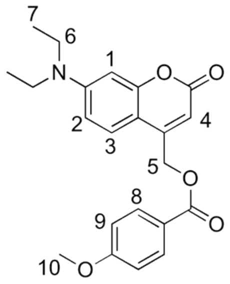

Purified by column chromatography (SiO_2, Pet. Et./EtOAc, 95/5 → 1/1 (v/v)), followed by preparative thin layer chromatography (SiO_2, CH_2Cl_2/EtOAc, 95/5 (v/v)). Isolated 45 mg (42% yield). ^{1}H NMR (400 MHz, $CDCl_3$): δ = 1.22 (t, *J* = 7.2 Hz, 6H, H7), 3.42 (q, *J* = 7.2 Hz, 4H, H6), 3.88 (s, 3H, H10), 5.44 (d, *J* = 1.3 Hz, 2H, H5), 6.25 (t, *J* = 1.3 Hz, 1H, H4), 6.55 (d, *J* = 2.5 Hz, 1H, H1), 6.62 (dd, *J* = 9.0, 2.6 Hz, 1H, H2), 6.92–6.98 (m, 2H, H9), 7.37 (d, *J* = 8.9 Hz, 1H, H3), 8.03–8.11 (m, 2H, H8). ^{13}C NMR (100 MHz, $CDCl_3$): δ = 12.6, 45.0, 55.7, 61.7, 98.2, 106.4, 106.7, 109.0, 114.0, 121.7, 124.6, 132.1, 149.9, 150.7, 156.4, 162.1, 164.0, 165.7. HRMS (ESI) calcd for $[C_{22}H_{24}N_1O_5]^+$: 382.1649, found 382.1647.

(7-(Diethylamino)-2-oxo-2*H*-chromen-4-yl)methyl benzoate (4b)

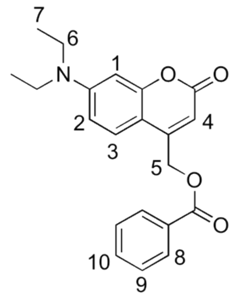

Purified by column chromatography (SiO_2, Pet. Et/EtOAc, 15/85 (v/v)), 43 mg (44% yield). ^1H NMR (400 MHz, $CDCl_3$): δ = 1.22 (t, *J* = 7.1 Hz, 6H, H7), 3.43 (q, *J* = 7.1 Hz, 4H, H6), 5.47 (d, *J* = 1.4 Hz, 2H, H5), 6.26 (t, *J* = 1.3 Hz, 1H, H4), 6.54 (d, *J* = 2.6 Hz, 1H, H1), 6.61 (dd, *J* = 9.0, 2.6 Hz, 1H, H2), 7.38 (d, *J* = 8.9 Hz, 1H, H3), 7.44–7.53 (m, 2H, H9), 7.59–7.64 (m, 1H, H10), 8.09–8.16 (m, 2H, H8). ^{13}C NMR (100 MHz, $CDCl_3$): δ = 12.6, 44.9, 62.0, 98.0, 106.2, 106.7, 108.9, 124.6, 128.8, 129.4, 130.0, 133.7, 149.7, 150.9, 156.5, 162.1, 166.0. HRMS (ESI) calcd for $[C_{21}H_{22}N_1O_4]^+$: 352.1543, found 352.1551.

(7-(Diethylamino)-2-oxo-2*H*-chromen-4-yl)methyl 4-(trifluoromethyl)benzoate (4c)

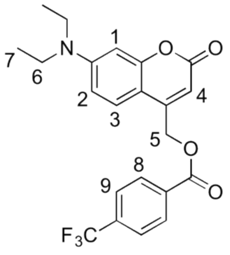

Purified by column chromatography (SiO_2, CH_2Cl_2/EtOAc, 99/1 (v/v)), followed by preparative thin layer chromatography (SiO_2, Pet. Et./EtOAc, 75/25 (v/v)). Isolated amount: 50 mg, 44%. ^1H NMR (400 MHz, $CDCl_3$): δ = 1.22 (t, *J* = 7.1 Hz, 6H, H7), 3.43 (t, *J* = 7.1 Hz, 4H, H6), 5.49 (d, *J* = 1.3 Hz, 2H, H5), 6.22 (t, *J* = 1.3 Hz, 1H, H4), 6.54 (d, *J* = 2.6 Hz, 1H, H1), 6.61, (dd, *J* = 9.0, 2.6 Hz, 1H, H2), 7.36 (d, *J* = 9.0 Hz, 1H, H3), 7.73–7.77 (m, 2H, H9), 8.15–8.27 (m, 2H, H8). ^{13}C NMR (100 MHz, $CDCl_3$): δ = 12.6, 44.9, 62.5, 98.1, 106.1, 106.8, 108.9, 123.6 (q, *J* = 272.8 Hz), 124.5, 125.8 (q, *J* = 3.7 Hz), 130.4, 132.6, 135.2 (q, *J* = 32.8 Hz), 149.1, 150.9, 156.5, 161.9, 164.8. HRMS (ESI) calcd for $[C_{22}H_{11}F_3N_1O_4]^+$: 420.1417, found 420.1424.

(7-(Diethylamino)-2-oxo-2*H*-chromen-4-yl)methyl pivaloylglycinate (4d)

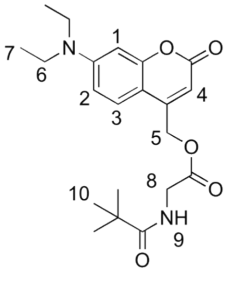

Purified by preparative thin layer chromatography (SiO_2, CH_2Cl_2/EtOAc, 8/2 (v/v)). Isolated amount: 90 mg, 72%. ^1H NMR (400 MHz, $CDCl_3$): δ = 1.21 (t, *J* = 7.1 Hz, 6H, H7), 1.46 (s, 9H, H10), 3.41 (q, *J* = 7.1 Hz, 4H, H6), 4.04 (d, *J* = 5.8 Hz, 2H, H8), 5.01 (tbr, *J* = 5.8 Hz, 1H, H9), 5.28 (d, *J* = 1.3 Hz, 2H, H5), 6.11 (t, *J* = 1.2 Hz, 1H, H4), 6.51 (d, *J* = 2.6 Hz, 1H, H1), 6.58 (dd, *J* = 9.0, 2.6 Hz, 1H, H2), 7.27 (d, *J* = 9.0 Hz, 1H, H3). ^{13}C NMR (100 MHz, $CDCl_3$): δ =12.6, 28.4, 42.6, 44.9,

62.3, 80.5, 98.0, 106.0, 107.0, 108.9, 124.6, 148.8, 150.9, 155.8, 156.5, 161.8, 170.0. HRMS (ESI) calcd for $[C_{21}H_{29}N_2O_6]^+$: 405.2020, found 405.2027.

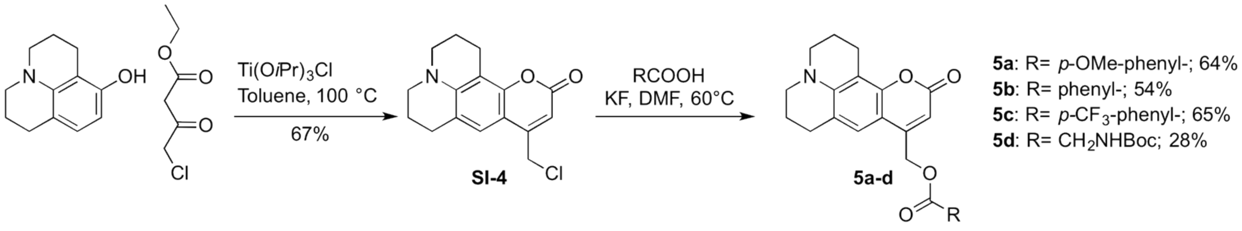

Scheme S3. Synthesis of compounds **5a-d**.

9-(Chloromethyl)-2,3,6,7-tetrahydro-1*H*,5*H*,11*H*-pyrano[2,3-*f*]pyrido[3,2,1-*ij*]quinolin-11-one (SI-4)[9]

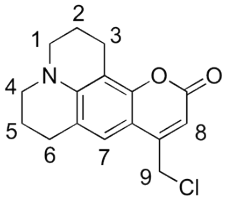

8-Hydroxyjulolidine (500 mg, 2.64 mmol) was added to a flame-dried flask and evacuated and backfilled with nitrogen three times. Dry toluene (40 mL), ethyl 4-chloroacetoacetate (434 mg, 2.64 mmol, 0.35 mL) and chlorotriisopropoxytitanium(IV) (5.28 mmol, 1 M in hexanes, 5.28 mL) were added to the flask. The reaction was warmed at 100 °C for 2 h. The solvent was removed under reduced pressure and the resulting oil was dissolved in CH_2Cl_2 and purified by column chromatography (SiO_2, Pet. Et./EtOAc, 85/15 → 3/7 (v/v), then changed to CH_2Cl_2/EtOAc, 1/9 → 2/8 (v/v)). Compound **SI-4** was isolated as an orange powder (510 mg, 67% yield). ^{1}H NMR (400 MHz, $CDCl_3$): δ = 1.77–2.12 (m, 4H, H2 and H5), 2.79 (t, J = 6.4 Hz, 2H, H6), 2.88 (t, J = 6.5 Hz, 2H, H3), 3.13–3.40 (m, 4H, H1 and H4), 4.54 (s, 2H, H9), 6.14 (s, 1H, H8), 7.03 (s, 1H, H7). ^{13}C NMR (100 MHz, $CDCl_3$): δ = 20.6, 20.7, 21.6, 27.9, 41.7, 49.7, 50.1, 106.2, 107.3, 108.5, 118.5, 121.3, 146.2, 150.1, 151.7, 162.2. HRMS (ESI) calcd for $[C_{16}H_{17}Cl_1N_1O_2]^+$: 290.0942, found 290.0946.

General procedure for the formation of julolidinyl-coumarin 4-methyl ester derivatives (5a-d)[9]

Potassium fluoride (3 eq.) was added to a flame-dried flask and flame-dried under high vacuum three times. Compound **SI-4** (1 eq.) and the corresponding acid (1.1 eq.) were added, and the system was evacuated and backfilled with nitrogen three times. The flask was wrapped in aluminum foil before adding dry DMF (0.06-0.07 M). The reaction was stirred at 60 °C for 2-3 h. The solution was diluted

with CH_2Cl_2 and the organic phase was washed twice with H_2O. The organic phase was dried over $MgSO_4$ and the solvent was removed under reduced pressure. Details of the purification procedure followed are given for each compound.

(11-Oxo-2,3,6,7-tetrahydro-1*H*,5*H*,11*H*-pyrano[2,3-*f*]pyrido[3,2,1-*ij*]quinolin-9-yl)methyl 4-methoxybenzoate (5a)

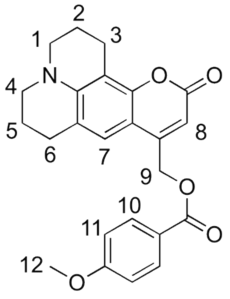

Amount of **SI-4** used: 100 mg. The reaction crude was separated via flash chromatography (SiO_2, CH_2Cl_2/EtOAc, 100/0 → 95/5 (v/v)). Isolated amount: 90 mg, 64% yield. ^1H NMR (400 MHz, $CDCl_3$): δ = 1.94–2.02 (m, 4H, H2 and H5), 2.78 (t, *J* = 6.1 Hz, 2H, H6), 2.90 (t, *J* = 6.5 Hz, 2H, H3), 3.15–3.36 (m, 4H, H1 and H4), 3.89 (s, 3H, H12), 5.42 (d, *J* = 1.4 Hz, 2H, H9), 6.23 (t, *J* = 1.4 Hz, 1H, H8), 6.79–7.03 (m, 2H, H11), 6.96 (s, 1H, H7), 7.91–8.17 (m, 2H, H10). ^{13}C NMR (100 MHz, $CDCl_3$): δ = 20.6, 20.8, 21.7, 27.9, 49.7, 50.1, 55.7, 61.7, 105.6, 106.0, 107.2, 114.0, 118.4, 120.6, 121.8, 132.1, 146.1, 150.1, 151.4, 162.5, 164.0, 165.7. HRMS (ESI) calcd for $[C_{24}H_{24}N_1O_5]^+$: 406.1649, found 406.1648.

(11-Oxo-2,3,6,7-tetrahydro-1*H*,5*H*,11*H*-pyrano[2,3-*f*]pyrido[3,2,1-*ij*]quinolin-9-yl)methyl benzoate (5b)

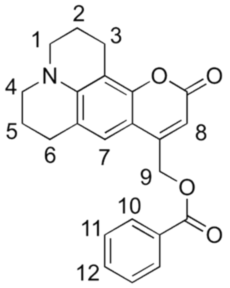

Amount of **SI-4** used: 100 mg. The reaction crude was separated via flash chromatography (SiO_2, CH_2Cl_2/EtOAc, 100/0 → 95/5 (v/v)). Isolated amount: 70 mg, 54% yield. ^1H NMR (400 MHz, $CDCl_3$): δ = 1.85–2.14 (m, 4H, H2 and H5), 2.78 (t, *J* = 6.3 Hz, 2H, H6), 2.90 (t, *J* = 6.5 Hz, 2H, H3), 3.20–3.34 (m, 4H, H1 and H4), 5.45 (d, *J* = 1.4 Hz, 2H, H9), 6.24 (t, *J* = 1.4 Hz, 1H, H8), 6.97 (s, 1H, H7), 7.38–7.53 (m, 2H, H11), 7.56–7.67 (m, 1H, H12), 8.07–8.15 (m, 2H, H10). ^{13}C NMR (100 MHz, $CDCl_3$): δ = 20.6, 20.7, 21.6, 27.9, 49.7, 50.1, 62.0, 105.6, 106.0, 107.2, 118.4, 120.6, 128.7, 129.4, 130.0, 133.7, 146.2, 149.8, 151.4, 162.5, 166.0. HRMS (ESI) calcd for $[C_{23}H_{22}N_1O_4]^+$: 376.1543, found 376.1539.

(11-Oxo-2,3,6,7-tetrahydro-1*H*,5*H*,11*H*-pyrano[2,3-*f*]pyrido[3,2,1-*ij*]quinolin-9-yl)methyl 4-(trifluoromethyl)benzoate (5c)

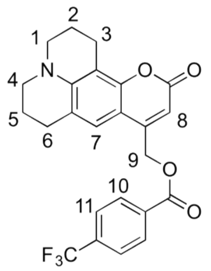

Amount of **SI-4** used: 50 mg. Purified by preparative thin layer chromatography (SiO_2, CH_2Cl_2/EtOAc, 96/4 (v/v)). Isolated amount: 50 mg, 65%. ^{1}H NMR (400 MHz, $CDCl_3$): δ = 1.94–2.02 (m, 4H, H2 and H5), 2.78 (t, *J* = 6.1 Hz, 2H, H6), 2.90 (t, *J* = 6.5 Hz, 2H, H3), 3.24–3.31 (m, 4H, H1 and H4), 5.47 (d, *J* = 1.3 Hz, 2H, H9), 6.20 (t, *J* = 1.3 Hz, 1H, H8), 6.95 (s, 1H, H7), 7.72–7.78 (m, 2H, H11), 8.21–8.25 (m, 2H, H10). ^{13}C NMR (100 MHz, $CDCl_3$): δ = 20.6, 20.7, 21.6, 27.9, 49.7, 50.1, 62.5, 105.8, 105.8, 107.2, 118.5, 120.5, 125.0 (q, *J* = 272 Hz), 125.8 (q, *J* = 3.8 Hz), 130.4, 132.6, 153.2 (q, *J* = 32.8 Hz), 146.3, 149.2, 151.4, 162.3, 164.8. HRMS (ESI) calcd for $[C_{24}H_{21}F_3N_1O_4]^+$: 444.1417, found 444.1420.

(11-Oxo-2,3,6,7-tetrahydro-1*H*,5*H*,11*H*-pyrano[2,3-*f*]pyrido[3,2,1-*ij*]quinolin-9-yl)methyl pivaloylglycinate (5d)

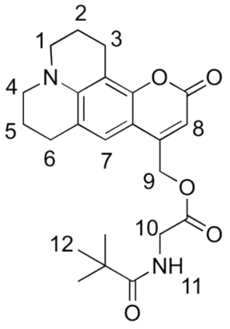

Amount of **SI-4** used: 80 mg. Purified by preparative thin layer chromatography (SiO_2, CH_2Cl_2/EtOAc, 8/2 (v/v)). Isolated amount: 40 mg, 28%. ^{1}H NMR (400 MHz, $CDCl_3$): δ = 1.46 (2, 9H, H12), 1.85–2.12 (m, 4H, H2 and H5), 2.76 (t, *J* = 6.1 Hz, 2H, H6), 2.88 (t, *J* = 6.5 Hz, 2H, H3), 3.26 (m, 4H, H1 and H4), 4.03 (d, *J* = 5.8 Hz, 2H, H10), 5.01 (t, *J* = 5.0 Hz, 1H, H11) 5.25 (d, *J* = 1.2 Hz, 2H, H9), 6.07 (t, *J* = 1.2 Hz, 1H, H8), 6.86 (s, 1H, H7). ^{13}C NMR (100 MHz, $CDCl_3$): δ =20.6, 20.7, 21.6, 27.9, 28.4, 42.6, 49.7, 50.1, 62.3, 80.4, 105.8, 106.1, 107.2, 118.4, 120.6, 146.2, 148.9, 151.4, 155.8, 162.2, 170.0. HRMS (ESI) calcd for $[C_{23}H_{29}N_2O_6]^+$: 429.2020, found 429.2027.

Photochemical Characterization of the Esters 3-5

General. The photochemistry was characterized in terms of molar extinction coefficient (ε) and quantum yield of photorelease (ϕ_{PA}). All measurements were conducted in 7/3 MeCN/phosphate buffered saline (PBS, pH 7.4) (v/v) mixtures at room temperature. Only for compounds **3-5a**, ε and ϕ_{PA} values were determined in 7/3 MeCN/EtOH or MeCN/pentanol (PeOH) (v/v) mixtures as well. Before every experiment, the purity of the sample was checked via LC/MS. The results for all the compounds measured are shown in Table S1.

Table S1. Photochemical parameters of compounds 3a-d, 4a-d, and 5a-d

Compound *[Co-solvent]*	λ_{max} (nm)	ε (mol^{-1} cm^{-1} L) x 10^{-4}	ϕ_{PA}
**3a** [PBS]	371	1.6(3)	0.014(5)
[EtOH]	367	1.86(9)	0.0070(8)
[PeOH]	367	1.91(9)	0.006(1)
**3b**	371	1.7(3)	0.016(5)
**3c**	372	1.6(1)	0.006(2)
**3d**	371	1.78(1)	0.0140(8)
**4a** [PBS]	383	2.18(6)	0.003(1)
[EtOH]	378	2.13(2)	0.0037(5)
[PeOH]	379	2.0(1)	0.0038(1)
**4b**	384	2.1(2)	0.0035(8)
**4c**	384	2.0(3)	0.0039(8)
**4d**	383	2.2(2)	0.0052(2)
**5a** [PBS]	400	1.8(1)	0.014(6)
[EtOH]	394	1.8(1)	0.011(1)
[PeOH]	394	1.86(8)	0.0097(4)
**5b**	400	1.9(2)	0.019(6)
**5c**	401	1.73(6)	0.006(1)
**5d**	399	1.8(1)	0.024(2)

Molar extinction coefficients. Extinction coefficients were determined by measuring the absorption spectra of three series of solutions differing by 10 μM steps in the 10–50 μM range. Two series were prepared by dilutions of two different stock solutions

of 0.2 mg mL^{-1} concentration in MeCN, whereas the third one was prepared from a stock solution of 0.1 mg mL^{-1}. All the stock solutions were freshly prepared and sonicated for 20 min before use. Supplementary Figures S1–3 show the acquired spectra for each compound, with each series of solutions plotted in a different color.

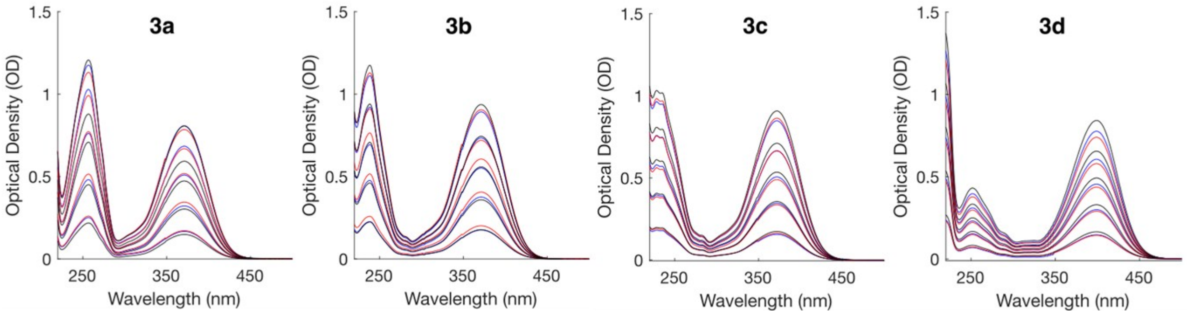

Figure S1. Absorption spectra for MeCN/PBS solutions of compounds **3a-d** in the 10–50 μM range, taken at 10 μM steps.

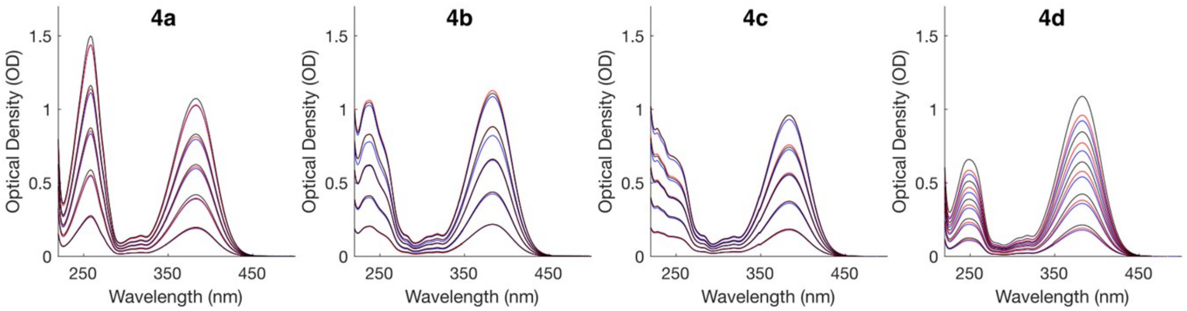

Figure S2. Absorption spectra for MeCN/PBS solutions of compounds **4a-d** in the 10–50 μM range, taken at 10 μM steps.

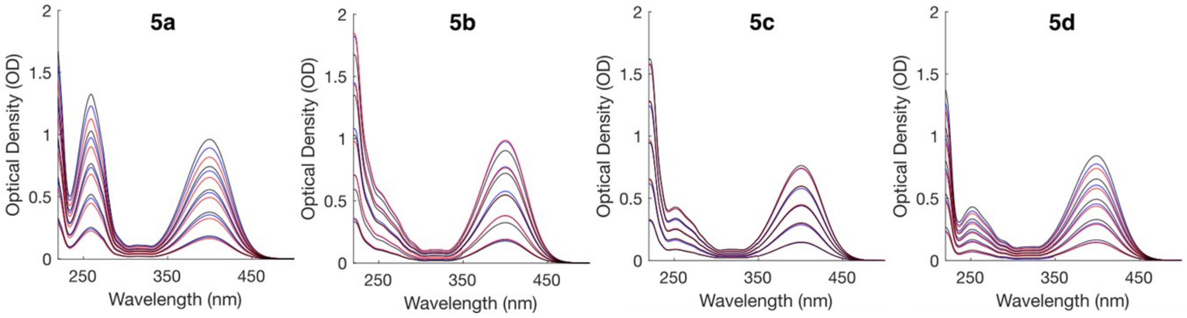

Figure S3. Absorption spectra for MeCN/PBS solutions of compounds **5a-d** in the 10-50 μM range, taken at 10 μM steps.

Quantum yield of photorelease. Quantum yields of photorelease were determined by following the disappearance of the starting material by analytical HPLC. HPLC and LC/MS analysis of the irradiation mixtures showed formation of the 4-methyl coumarin alcohols, the relative acid, and of the corresponding 4-methylchloride coumarins. The latter are likely formed from the trapping of photochemically generated coumarin 4-methyilium cations by chloride ions present in solution. According to the manufacturer's specifications,[1] the PBS used is 155 mM in NaCl. All the HPLC runs were performed with gradient 1 described in the general part, with the exception of compound **3d**, for which gradient 2 was used. This change was necessary because the 4-chloromethyl product co-eluted with the starting material when run with gradient 1.

The irradiations were performed on 3.5 mL of ~300 µM solutions of compounds in quartz cuvette under magnetic stirring. An LED emitting at 405 nm (Roithner Lasertechnik, LED405-06V) was employed as a light source, and placed in contact with the cuvette containing the solution. The incident light intensity (~8 mW) was measured before every measurement with a digital power meter (Thorlabs, PM100D) equipped with a Si photodiode detector (Thorlabs, S120VC). The power meter was used also to measure the light transmitted through the cuvette for the whole duration of the experiment, to monitor in real time the effective absorbed power. Reducing the incident light intensity by a factor of 10 by placing a neutral density (ND) linear filter (Thorlabs, NDL-25C-2) between the LED and the cuvette did not affect the measured quantum yield of photorelease. For HPLC analysis, 20 µL of the irradiated solution were diluted to up to 200 µL with MeCN/PBS 7/3 (v/v), of which 100 µL were injected for analysis. To evaluate the concentration of the samples, a calibration curve was built for the starting materials by injecting duplicate solutions of 6 different concentrations in the 5–50 µM range and evaluating the area under the corresponding peaks as a function of the nominal solution concentration. For each compound, three series of solutions were measured to build the calibration curves in triplicate. Two series were prepared by dilutions of two different stock solutions of 0.2 mg mL^{-1} in MeCN, whereas the third one was prepared from a stock solution of 0.1 mg mL^{-1}. All the stock solutions were freshly prepared and sonicated 20 min before use. Prior to taking the aliquots for HPLC analysis, the irradiated solution in

[1] The composition of the PBS buffer used can be found online at http://www.thermofisher.com/ch/en/home/technical-resources/media-formulation.161.html

the cuvette was manually stirred by pipetting. Multiple time points were taken during the irradiations in order to build a trend line for the disappearance of the starting material. Representative runs for every compound are shown in Figure S4. Every run was repeated at least three times.

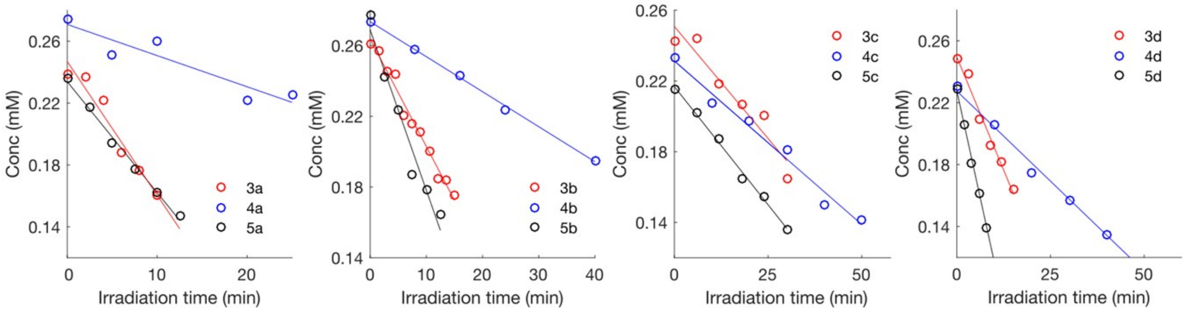

Figure S4. Representative data for the irradiations experiments of compounds **5a-d, 6a-d** and **7a-d**. Open circles and solid lines represent experimental points and relative fits, respectively.

The angular coefficients α of the fitting curves in Figure S4 correspond to the consumption rates of starting material in mmol L^{-1} s^{-1} and can easily be converted in molec s^{-1} according to equation E1,

$$\alpha(molec\, s^{-1}) = \alpha\,(mmol\, L^{-1} s^{-1})\, V(L)\, N_a 10^{-3} \tag{E1}$$

where V is the irradiated volume, N_a is Avogadro's number, and 10^{-3} is a conversion factor from mmol to mol. The angular coefficients and the corresponding 95% confidence interval were extracted using the MATLAB built-in curve fitting application. The experimental points were fit to a first order polynomial. The relative errors on the coefficient REα were evaluated according to equation E2

$$RE_\alpha = \frac{U_{CI} - \alpha}{\alpha} = \frac{\alpha - L_{CI}}{\alpha} \tag{E2}$$

where U_{CI} and L_{CI} are the upper and lower boundary of the 95% confidence interval of α, respectively. The relative errors on the measured quantum yields *RE*ϕ were taken to be equal to the relative errors of the corresponding angular coefficients *RE*α. The error bars for the individual ϕ_{PA} values plotted in Figure 3 of the main

manuscript are the absolute errors for each measurement of ϕ_{PA} obtained from these relative errors.

The rate of absorbed photons per second I_A can be evaluated from the measured absorbed power P_A and the energy of a photon of 405 nm wavelength, E_p according to equation E3

$$I_A = \frac{P_A}{E_p} \qquad \text{(E3)}$$

The energy of a photon E_p is calculated according to equation E4

$$E_p = \frac{hc}{\lambda} \qquad \text{(E4)}$$

where h is Planck's constant, c is the speed of light and λ is the wavelength of the photon. The quantum yield of photorelease ϕ_{PA} is then given by equation E5.

$$\varphi_{PA} = \frac{\alpha}{P_A} \qquad \text{(E5)}$$

Photophysical Characterization of the Fluorophores 1 and 2

The photophysics was characterized in terms of fluorescence quantum yield (ϕ_F), fluorescence lifetime (τ_F) and non-radiative rate constant (k_{NR}) in a series of solvents of different hydrogen-bonding ability (Table S2 and S3).

The k_{NR} values were obtained from the other two parameters according to equations E6 and E7.

$$\varphi_F = k_{rad}\tau_F \qquad \text{(E6)}$$

$$k_{NR} = k_F - k_{rad} \qquad \text{(E7)}$$

where k_{rad} is the radiative rate constant and $k_F = 1/\tau_F$.

Table S2. Photophysical parameters of 1 in various solvents.

Solvent	Δf^a	α^b	ϕ_F	τ_F (ns)	k_{NR} (ns^{-1})
MeCN	0.61	0.19	0.81	3.95	0.05
H_2O	0.64	1.19	0.92	5.0	0.015
D_2O	0.64	1.19	0.84	5.2	0.03
TFE	0.64	1.51	0.89	5.1	0.02
HFP	0.62	1.96	0.90	5.4	0.02
DMSO	0.53	0	-	3.3	-

a) Onsager polarity function: $\Delta f = 2(\varepsilon-1)/(2\varepsilon+1) - 2(n^2-1)/(2n^2+1)$[18]

b) Kamlet-Taft parameter[19]

Table S3. Photophysical parameters of 2 in various solvents.

Solvent	Δf^a	α^b	ϕ_F	τ_F (ns)	k_{NR} (ns^{-1})
MeCN	0.61	0.19	0.84	3.95	0.040
PeOH	0.50	0.84	0.82	3.8	0.047
BuOH	0.53	0.79	0.79	3.8	0.055
EtOH	0.58	0.86	0.66	3.4	0.10
MeOH	0.615	0.98	0.40	2.1	0.28
H_2O	0.64	1.19	0.06	0.40	2.35
D_2O	0.64	1.19	0.07	0.45	2.07
TFE	0.64	1.51	0.46	2.85	0.19
HFP	0.62	1.96	0.52	2.87	0.17
DMSO	0.53	0	-	2.7	-

a) Onsager polarity function: $\Delta f = 2(\varepsilon-1)/(2\varepsilon+1) - 2(n^2-1)/(2n^2+1)$[18]

b) Kamlet-Taft parameter[19]

Fluorescence up-conversion and TA

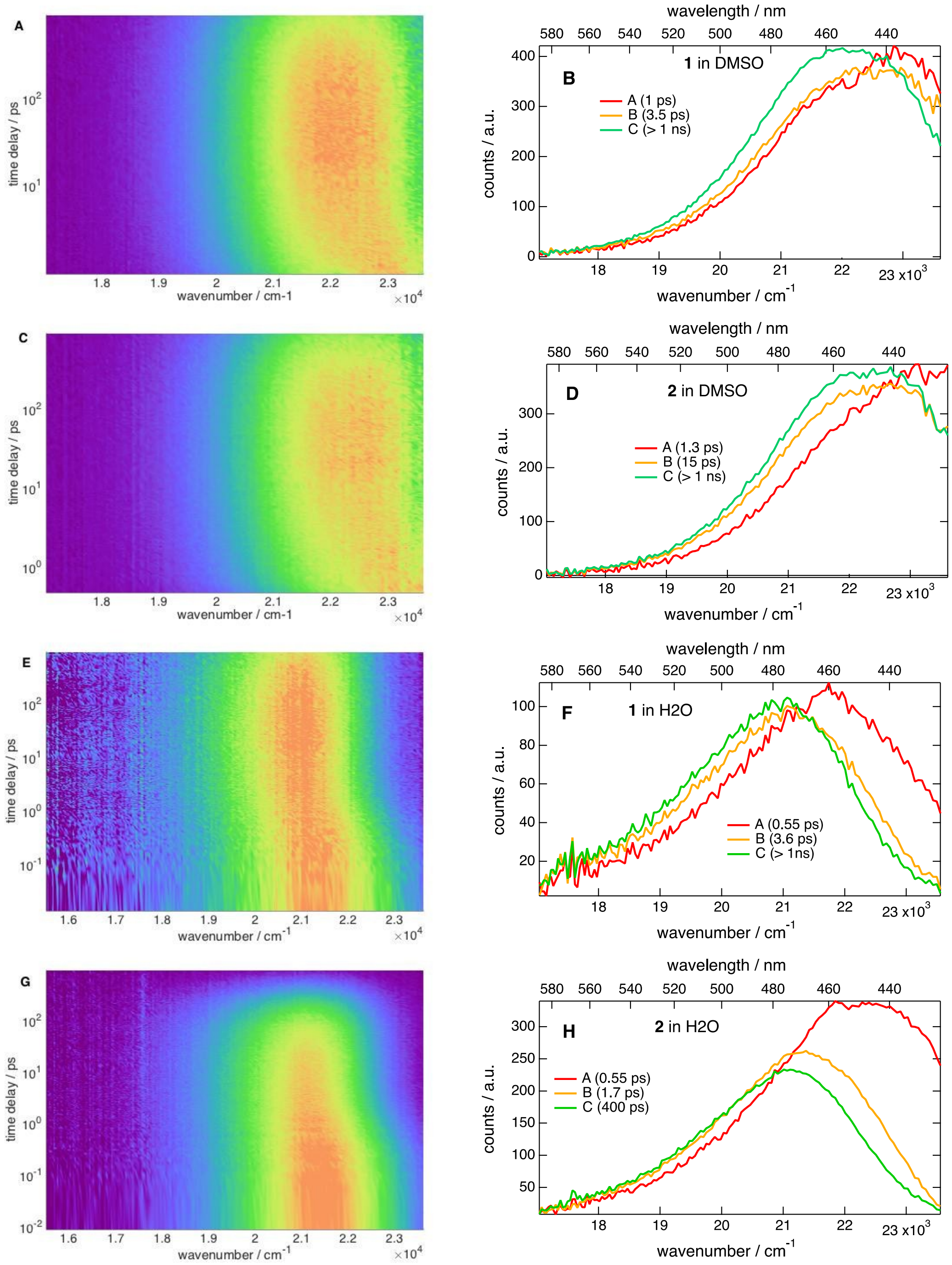

Figure S5. (A, C, E, G) Time-resolved fluorescence of **1** and **2** DMSO and water. (b) EAES obtained from global analysis assuming a A→B→C→ scheme (the associated time constants are listed in the legend).

In Figure S6 we compare the EADS obtained from the analysis of the fs TA data obtained with **1** and **2** in water. The TA spectra are dominated by a negative band

due to stimulated emission (SE). Positive bands can be observed below 400 nm and above 600 nm. They can be associated with excited-state absorption (ESA). Clearly ESA and SE overlap. In general, these spectra are almost the same for **1** and **2** and do not present strong spectral dynamics. The long-lived EADS measured with **2** (C in Figure S6D) decays in ~400 ps as found by fluorescence. After this process, a very small residual spectrum decaying on a much longer time scale can be observed. Slower TA measurements were performed to find more about this residual. It was found to be also present with **2** and in ACN. Its lifetime is of the order of 1 microsecond. This spectrum is most probably due to the triplet state of **1** and **2** populated upon ISC from the S_1 state. The triplet yield can be estimated to be less than 5% and, therefore, this process is not responsible for the quenching of the fluorescence of **2** in water.

In summary, these TA data do not point to the presence of an intermediate state populated upon quenching of **2** fluorescence in water. This quenching leads most probably to the population of the ground state. However, the presence of an intermediate with a much shorter lifetime than the S_1 state of **2** (i.e. $< \sim 50$ ps) cannot be totally excluded.

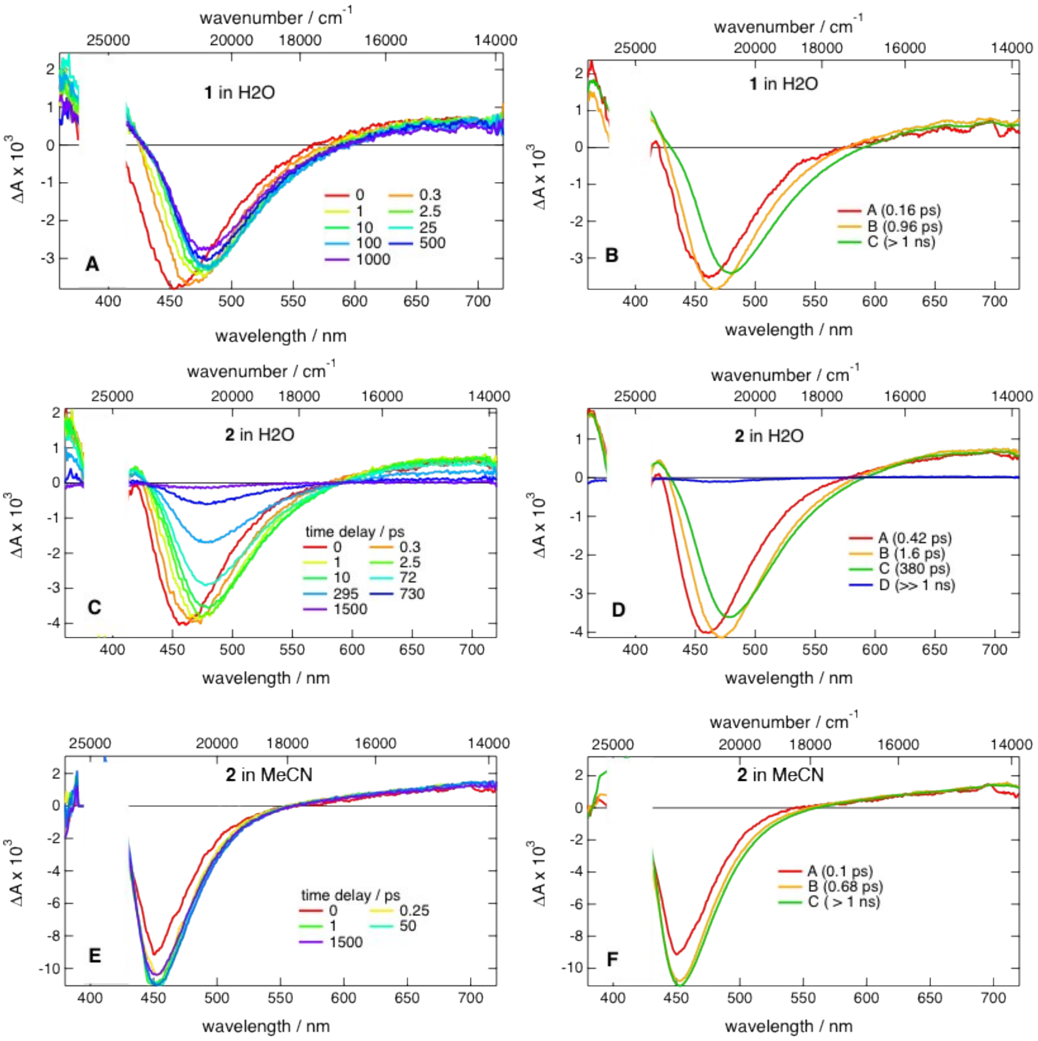

Figure S6. (A, C, E) TA spectra recorded at various time delays after excitation of **1** in water (A) and **2** in water (C) and in ACN (**E**). (B, D, F) Evolution associated difference absorption spectra (EADS) obtained from global analysis of the TA data assuming three or four successive exponential steps, A→B→C→(D→) (the associated time constants are listed in the legend.)

Dipole Moment Determination

The solvatochromic behavior of the absorption and emission bands has been investigated in a series of solvents and Figure S7).

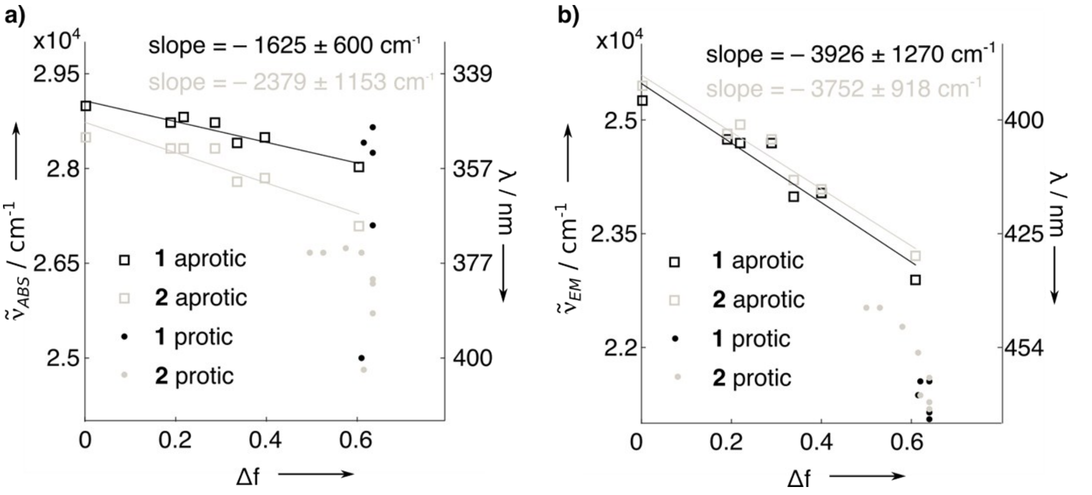

Figure S7. (a) Absorption and (b) fluorescence solvatochromism of **1** and **2** in non-protic and in protic solvents. The solids lines are linear fit to the non-protic solvents data. The errors shown for the slopes correspond to the 95% confidence intervals.

Table S4. Absorption and emission maxima of 1 and 2 in aprotic solvents.

Solvent	Δf^{a}	E_{abs}(**2**) (cm^{-1})	E_{fl}(**2**) (cm^{-1})	E_{abs}(**1**) (cm^{-1})	E_{fl}(**1**) (cm^{-1})
cyclohexane	0	28490	25445	28986	25253
dipentyl ether	0.22	28329	24938	28818	24691
dibutyl ether	0.19	28329	24814	28736	24752
diisopropyl ether	0.29	28330	24752	28736	24691
butyl acetate	0.34	27778	24213	28409	23981
ethyl acetate	0.40	27855	24096	28490	24038
MeCN	0.61	27100	23202	28011	22883

a) Onsager polarity function: $\Delta f = 2(\varepsilon-1)/(2\varepsilon+1) - 2(n^2-1)/(2n^2+1)$[18]

Table S5. Absorption and emission maxima of 1 and 2 in protic solvents.

Solvent	Δf^a	E_{abs}(**2**) (cm^{-1})	E_{fl}(**2**) (cm^{-1})	E_{abs}(**1**) (cm^{-1})	E_{fl}(**1**) (cm^{-1})
PeOH	0.50	26667	22523		
BuOH	0.53	26667	22523		
EtOH	0.58	26738	22272		
MeOH	0.615	26667	21930	25000	21370
H_2O	0.64	26247	21277	28249	21053
D_2O	0.64	26178	21186	28653	21142
trifluoroethanol	0.64	25707	21599	27100	21552
hexafluoroisopropanol	0.62	24814	21368	28409	21552

a) Onsager polarity function: $\Delta f = 2(\varepsilon-1)/(2\varepsilon+1) - 2(n^2-1)/(2n^2+1)$[18]

The solvent dependence of the absorption is more pronounced for **2** than for **1**. On the other hand, both dyes show the same polarity dependence of the fluorescence. Finally, the solvatochromism of the emission is markedly larger than that of the absorption.

The slope of these solvatochromic plots (after conversion to Joules) corresponds to:[18]

$$slope_{abs} = -\frac{\mu_g \Delta\mu}{4\pi\varepsilon_0 a^3} \qquad \text{(E8)}$$

$$slope_F = -\frac{\mu_e \Delta\mu}{4\pi\varepsilon_0 a^3} \qquad \text{(E9)}$$

where μ_g and μ_e are the permanent dipole moments in the ground and excited states, respectively, $\Delta\mu = \mu_e - \mu_g$, ε_0 is the vacuum permittivity, and *a* is the cavity radius. If one assumes that μ_g and μ_e are parallel and that *a* does not change significantly upon excitation, both μ_g and μ_e can be calculated from these solvatochromic slopes. The cavity radii of **1** and **2** obtained from DFT calculations (B3LYP functional and 6-31G basis set as implemented in Gaussian09 rev C.01[20] with the keyword ‘volume=tight’) were taken as 4.07 and 4.09 Å, respectively. The resulting dipole moments are listed in Table S6. The biggest source of error on these quantities is the cavity radius, which enters the equations at the third power. The

model assumes a spherical cavity and the molecules are far from spheres, therefore the only meaningful and reliable quantities are the solvatochromic slopes. On the other hand the DFT calculations show that the cavity radius of both molecules should be almost the same for **1** and **2**. We can conclude that the differences of solvatochromic slopes between **1** and **2** reflect different dipole moments and not differences in cavity radii.

Table S6. Ground- and excited-state electric dipole moments deduced from the solvatochromic slopes using eqs. E8 and E9.

Dye	μ_g (D)	μ_e (D)	$\Delta\mu$ (D)
**1**	3.9	9.5	5.6
**2**	7.5	11.8	4.3

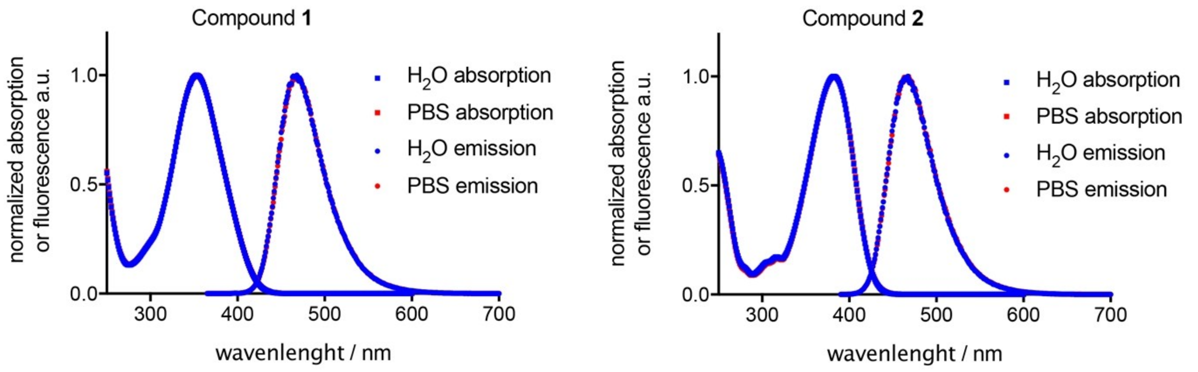

Figure S8. Absorption and emission spectra of compounds **1** and **2** in pure water and PBS. The identical steady-state spectra reveal that it is not essential to buffer the aqueous solution to perform time-resolved studies.

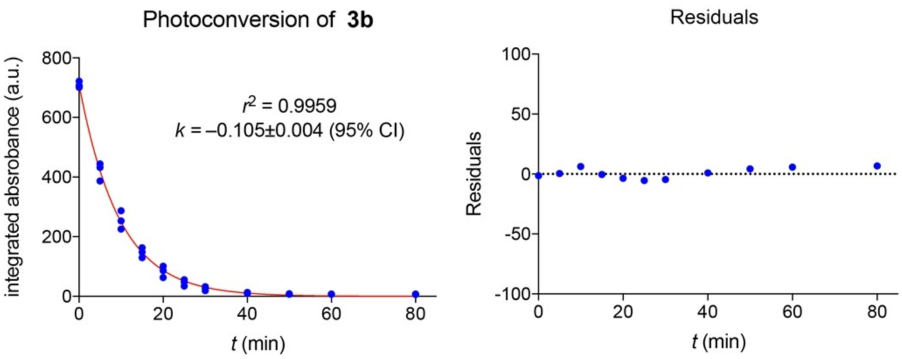

Figure S9. Consumption of compound **3b** (150 μM in PBS/CH_3CN, 3:7) upon irradiation at 405 nm followed by HPLC. Results from three independent experiments are shown. Experimental data (blue dots, left graph) was fit to a monoexponential function (red line, left graph).

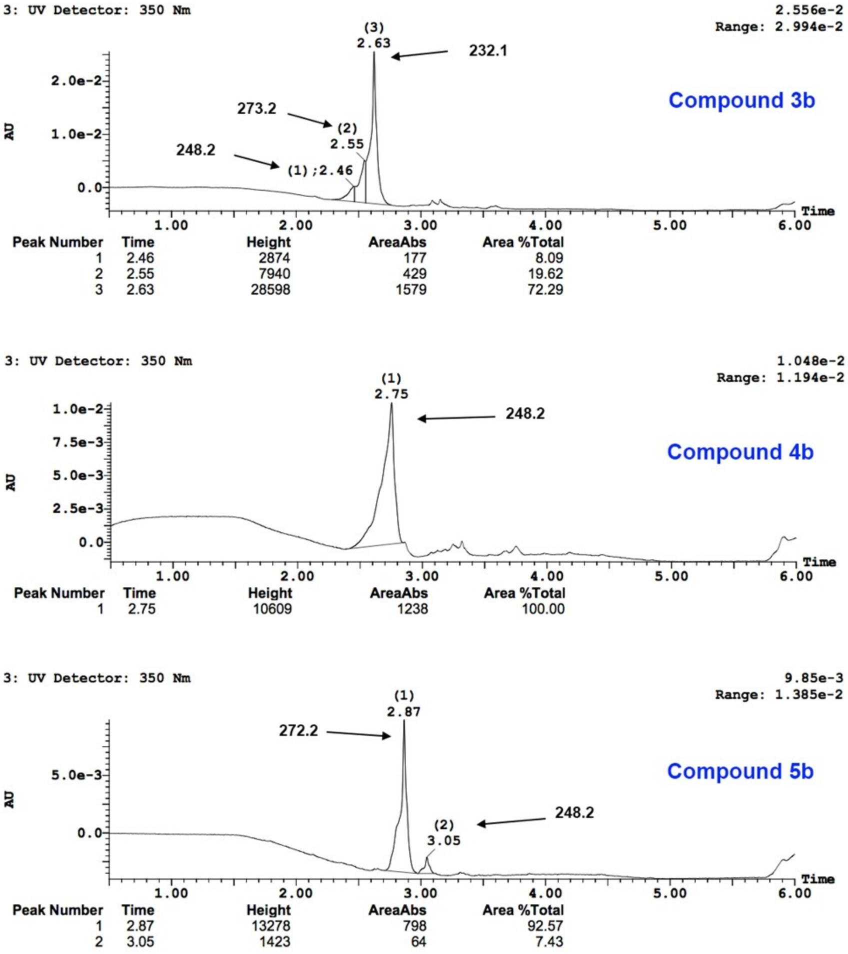

Figure S10. LC-MS traces after completion of the photochemical conversion of compounds **3b**, **4b** and **5b**. These chromatograms demonstrate that the photochemical reactions are largely clean, giving mostly the corresponding alcohols, and only traces of other unidentified products are formed.

References

1 A. M. Brouwer, *Pure Appl. Chem.*, 2011, **83**, 2213.

2 L. Zhao, L. J. Pérez Lustres, V. Farztdinov, N. Ernsting, *Phys. Chem. Chem. Phys.*, 2005, **7**, 1716.

3 N. Banerji, G. Duvanel, A. Perez-Velasco, S. Maity, N. Sakai, S. Matile, E. Vauthey, *J. Phys. Chem. A*, 2009, **113**, 8202.

4 B. Lang, S. Mosquera-Vázquez, D. Lovy, P. Sherin, V. Markovic, E. Vauthey, *Rev. Sci. Instrum.*, 2013, **84**, 073107.

5 I. H. M. van Stokkum, D. S. Larsen, R. van Grondelle, *Biochim. Biophys. Acta, Bioenerg.*, 2004, **1657**, 82.

6 P. Fita, E. Luzina, T. Dziembowska, C. Radzewicz, A. Grabowska, *J. Chem. Phys.*, 2006, **125**, 184508.

7 P.-A. Muller, C. Högemann, X. Allonas, P. Jacques, E. Vauthey, *Chem. Phys. Lett.*, 2000, **326**, 321.

8 J. B. Grimm, B. P. English, J. Chen, J. P. Slaughter, Z. Zhang, A. Revyakin, R. Patel, J. J. Macklin, D. Normanno, R. H. Singer, T. Lionnet, L. D. Lavis, *Nat. Methods*, 2015, **12**, 244.

9 A. M. Piloto, G. Hungerford, S. P. G. Costa, M. S. T. Gonçalves, *Eur. J. Org. Chem.* **2013**, 7715.

10 J. Zhang, Y.-Q. Sun, J. Liu, Y. Shi, W. Guo, *Chem. Commun.*, 2013, **49**, 11305.

11 J. Kozlowski, C. Coburn, W. Yu, L. Tong, B. Hu, B. Zhong, J. Hao, D. Wang, J. Tao, *Fused Tetracyclic Heterocyclic Compounds and Methods of Use Thereof for the Treatment of Viral Diseases*, 2015, WO2015/094998 A1.

12 X. Li, M. Reuman, R. K. Russell, R. Adams, R. Ma, S. Beish, S. Branum, S. Youells, J. Roberts, N. Jain, R. Kanojia, Z. Sui, *Org. Process Res. Dev.*, 2007, **11**, 414.

13 R. M. Kanojia, N. Jain, J. Xu, Z. Sui, *Tetrahedron Lett.*, 2004, **45**, 5837.

14 N. Chen, N. Jain, J. Xu, M. Reuman, X. Li, R. K. Russell, Z. Sui, *Tetrahedron Lett.*, 2006, **47**, 5909.

15 X. Zhang, Z. Sui, *Synthesis*, 2006, **15**, 2568.

16 D. Mazunin, *Formation and Functionalization of Hydrogels with the Potassium Acyltrifluoroborate (KAT) Ligation,* 2016, PhD Dissertation No. 23564*,* ETH

Zurich.

17 L. Fournier, I. Aujard, T. Le Saux, S. Maurin, S. Beaupierre, J.-B. Baudin, L. Jullien, *Chem. – Eur. J.*, 2013, **19**, 17494.

18 P. Suppan, *J. Photochem. Photobiol. A,* 1990, **50**, 293.

19 R. W. Taft, M. J. Kamlet, *J. Am. Chem. Soc.*, 1976, **98**, 2886.

20 M. J. Frisch, G. W. Trucks, H. B. Schlegel, G. E. Scuseria, M. A. Robb, J. R. Cheeseman, G. Scalmani, V. Barone, B. Mennucci, G. A. Petersson, H. Nakatsuji, M. Caricato, X. Li, H. P. Hratchian, A. F. Izmaylov, J. Bloino, G. Zheng, J. L. Sonnenberg, M. Hada, M. Ehara, K. Toyota, R. Fukuda, J. Hasegawa, M. Ishida, T. Nakajima, Y. Honda, O. Kitao, H. Nakai, T. Vreven, J. A. Montgomery, Jr., J. E. Peralta, F. Ogliaro, M. Bearpark, J. J. Heyd, E. Brothers, K. N. Kudin, V. N. Staroverov, R. Kobayashi, J. Normand, K. Raghavachari, A. Rendell, J. C. Burant, S. S. Iyengar, J. Tomasi, M. Cossi, N. Rega, J. M. Millam, M. Klene, J. E. Knox, J. B. Cross, V. Bakken, C. Adamo, J. Jaramillo, R. Gomperts, R. E. Stratmann, O. Yazyev, A. J. Austin, R. Cammi, C. Pomelli, J. W. Ochterski, R. L. Martin, K. Morokuma, V. G. Zakrzewski, G. A. Voth, P. Salvador, J. J. Dannenberg, S. Dapprich, A. D. Daniels, Ö. Farkas, J. B. Foresman, J. V. Ortiz, J. Cioslowski, and D. J. Fox, Gaussian 09, Rev. C.01 (Gaussian, Inc., Wallingford CT, 2009).

NMR Spectra

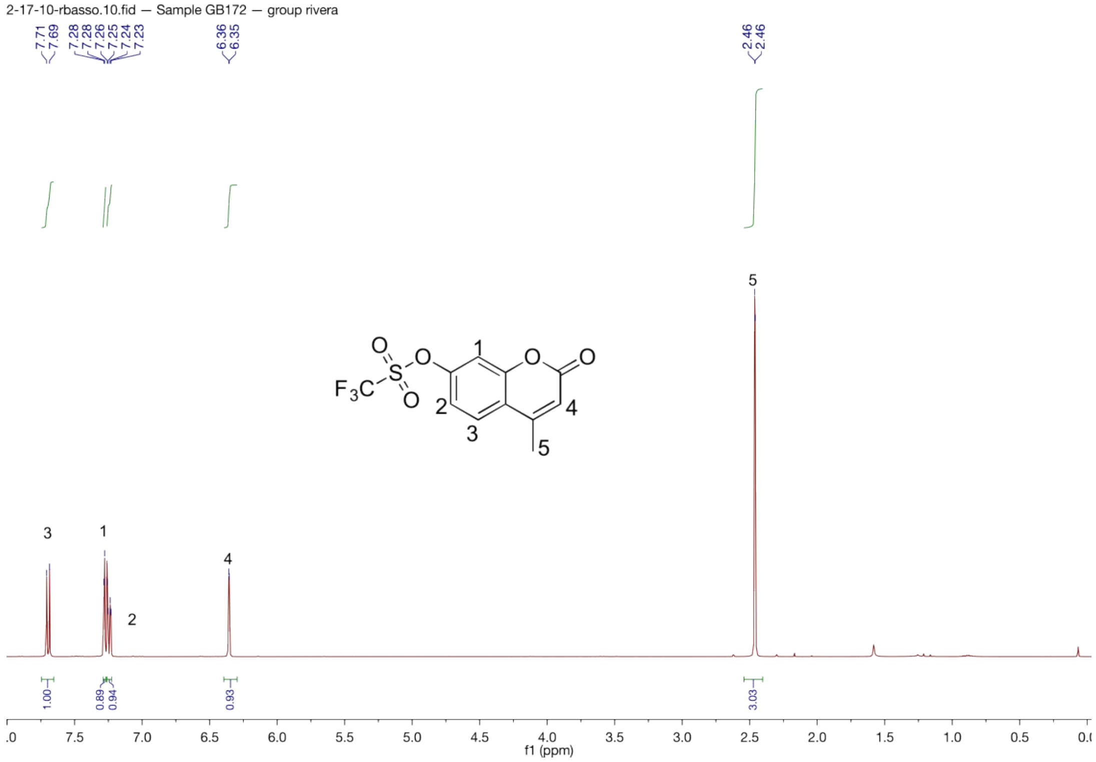

^{1}H NMR ($CDCl_3$, 400 MHz) spectrum of compound **SI-1**

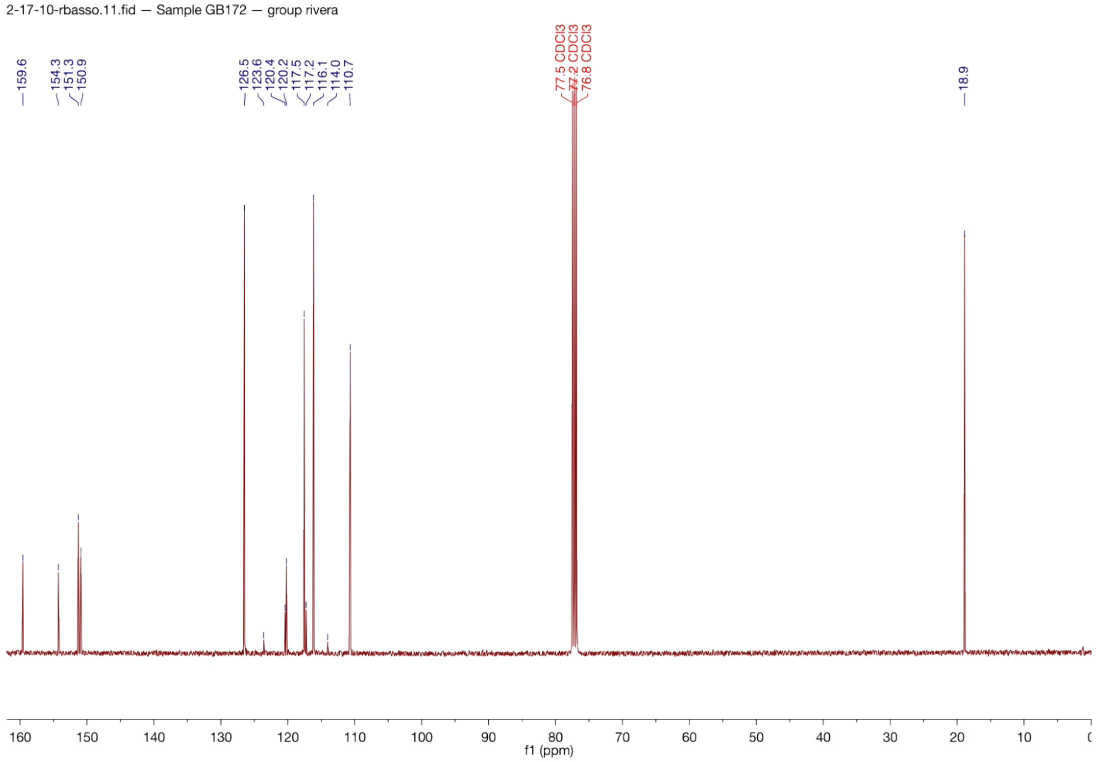

^{13}C NMR ($CDCl_3$, 100 MHz) spectrum of compound **SI-1**

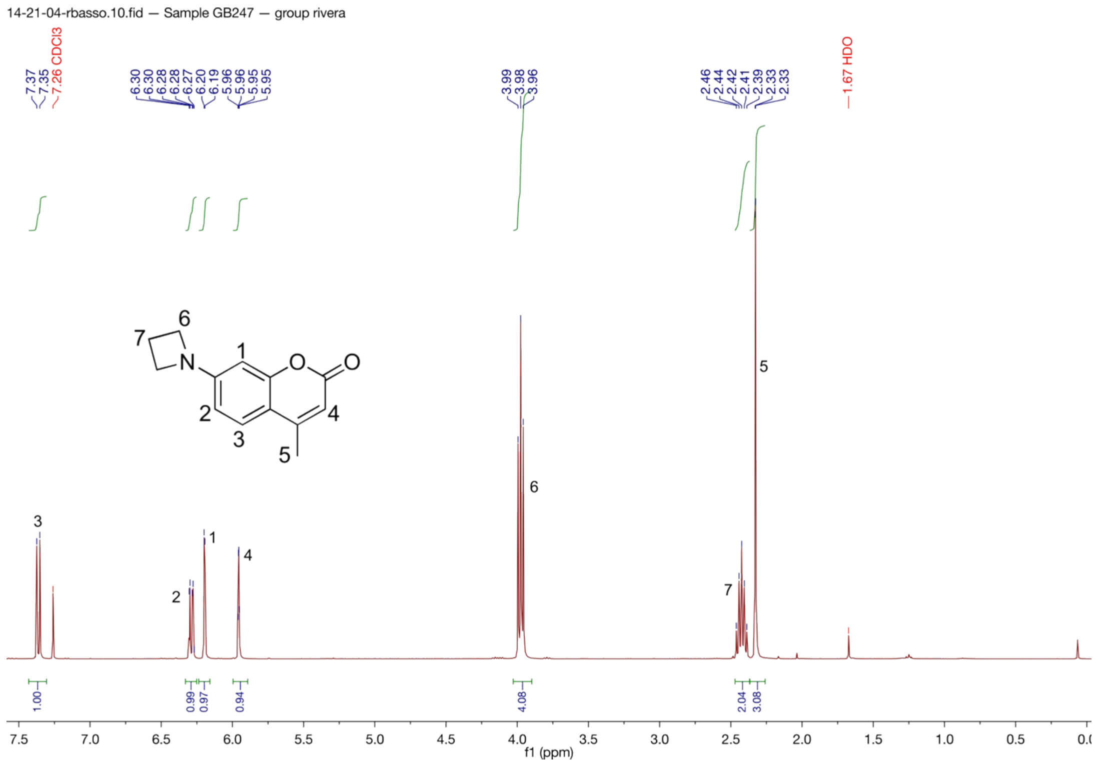

^{1}H NMR ($CDCl_3$, 400 MHz) spectrum of compound **1**

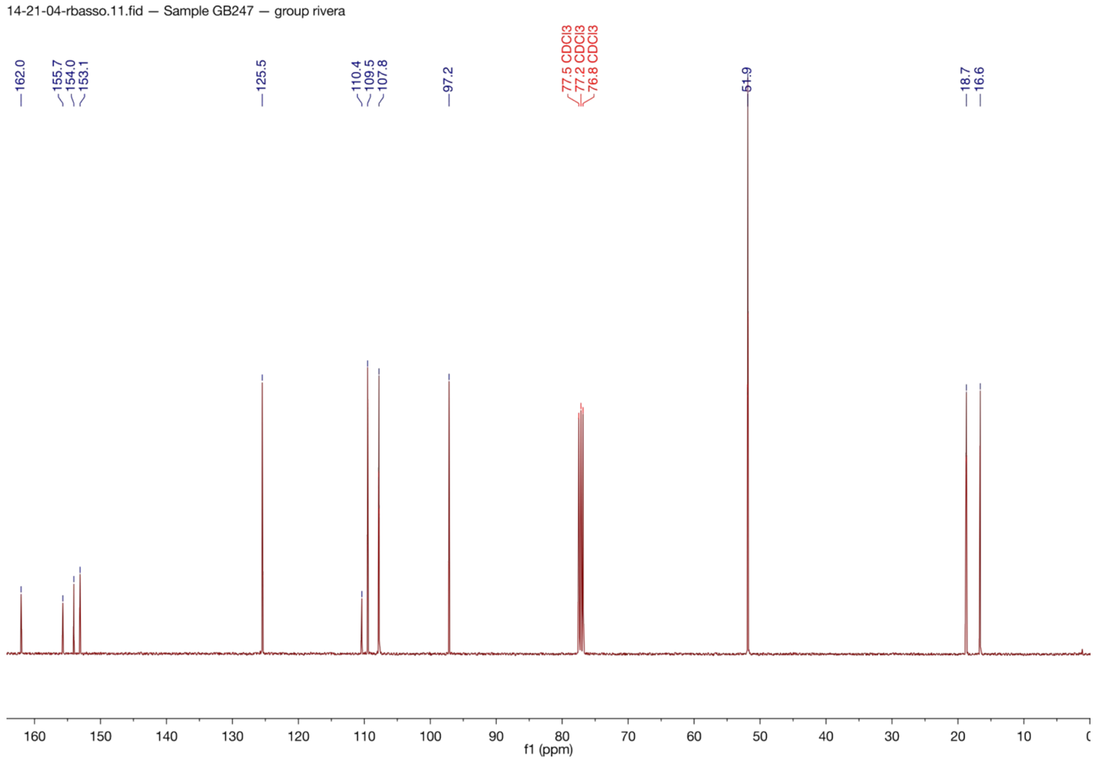

^{13}C NMR ($CDCl_3$, 100 MHz) spectrum of compound **1**

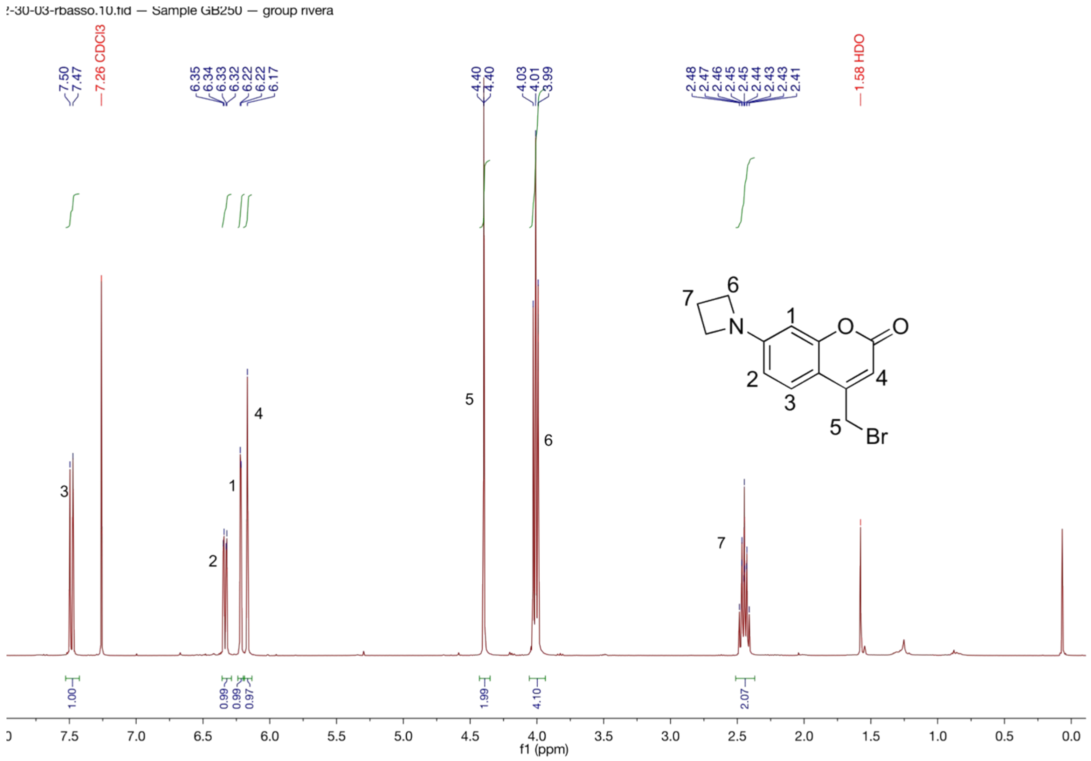

^{1}H NMR ($CDCl_3$, 400 MHz) spectrum of compound **SI-2**

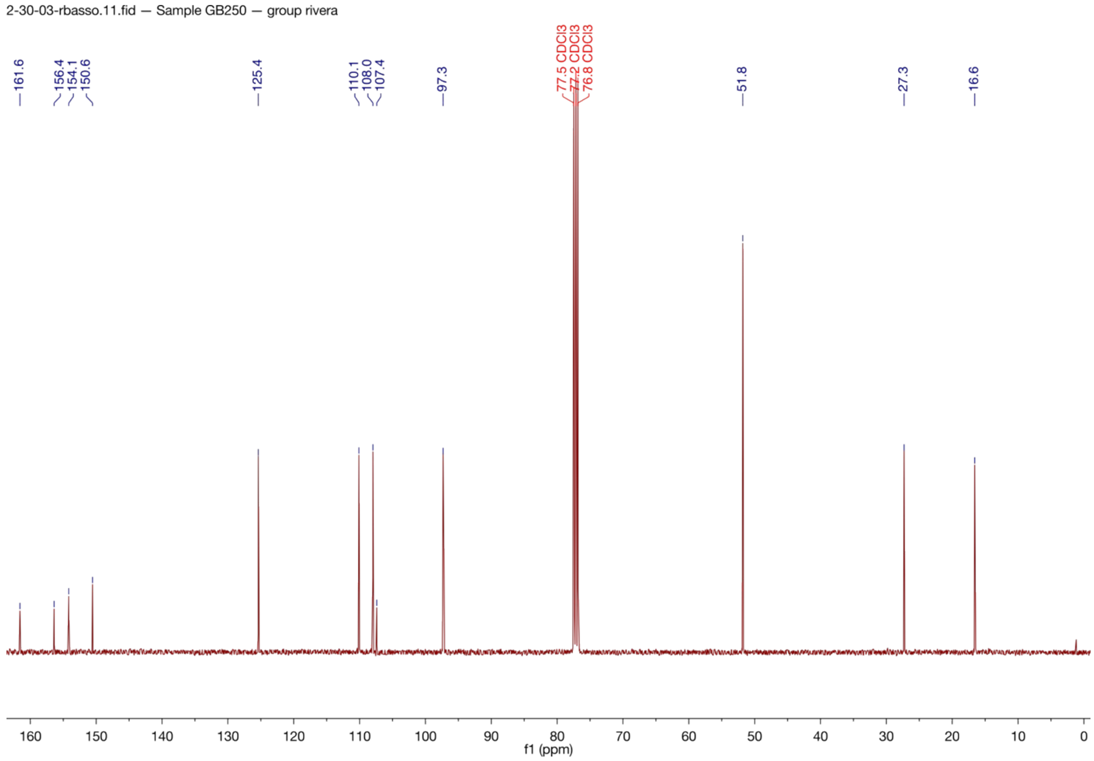

^{13}C NMR ($CDCl_3$, 100 MHz) spectrum of compound **SI-2**

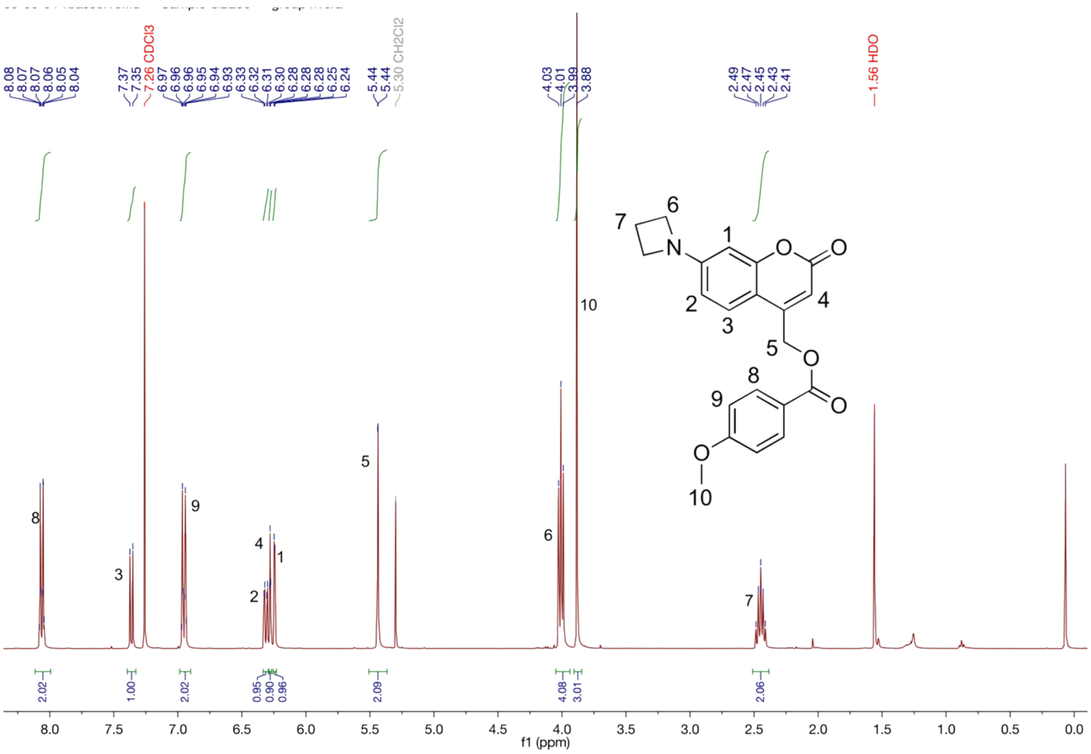

^{1}H NMR ($CDCl_3$, 400 MHz) spectrum of compound **3a**

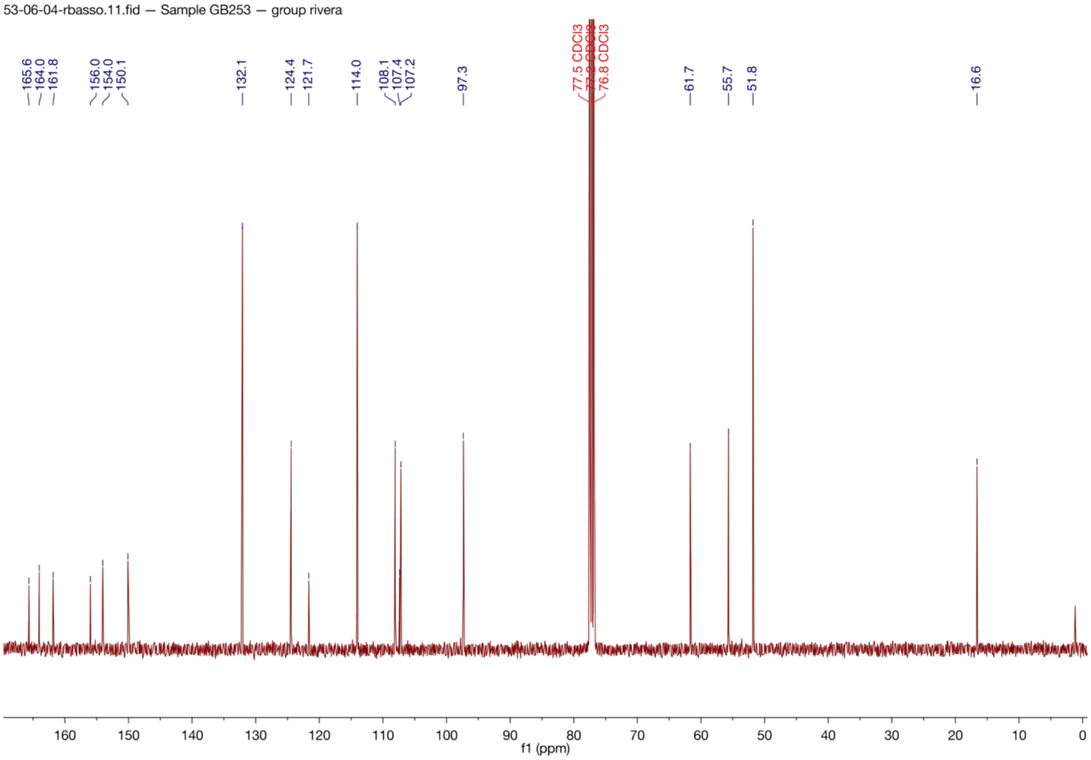

^{13}C NMR ($CDCl_3$, 100 MHz) spectrum of compound **3a**

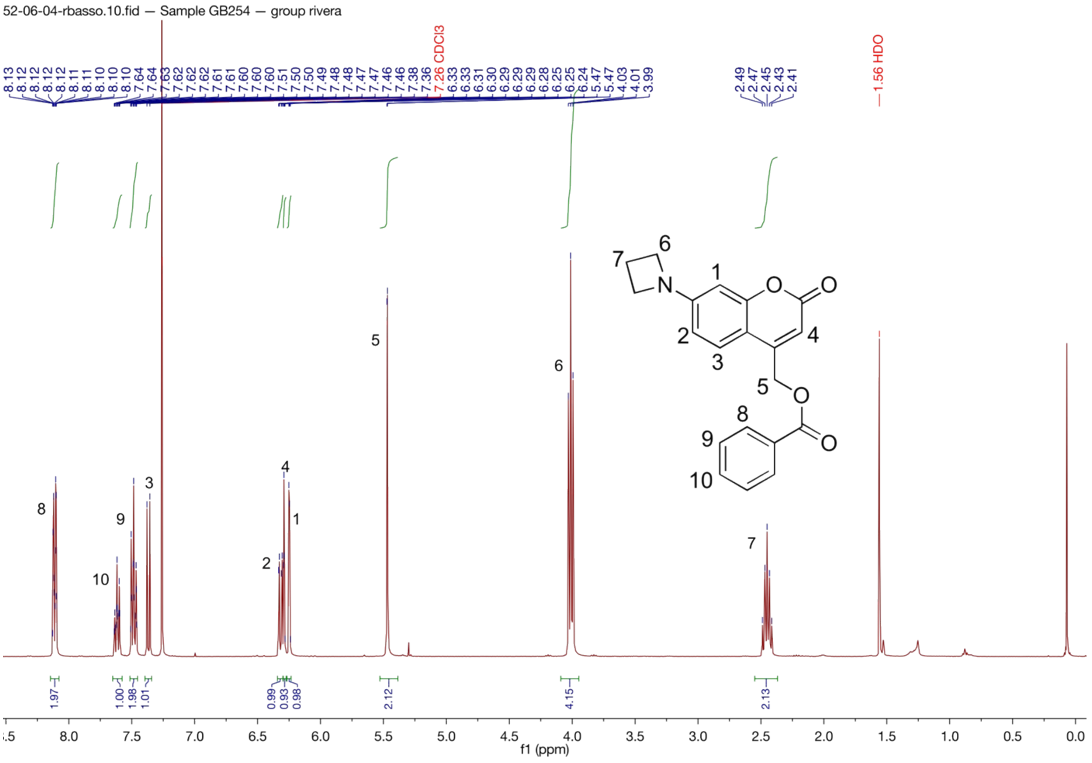

^{1}H NMR ($CDCl_3$, 400 MHz) spectrum of compound **3b**

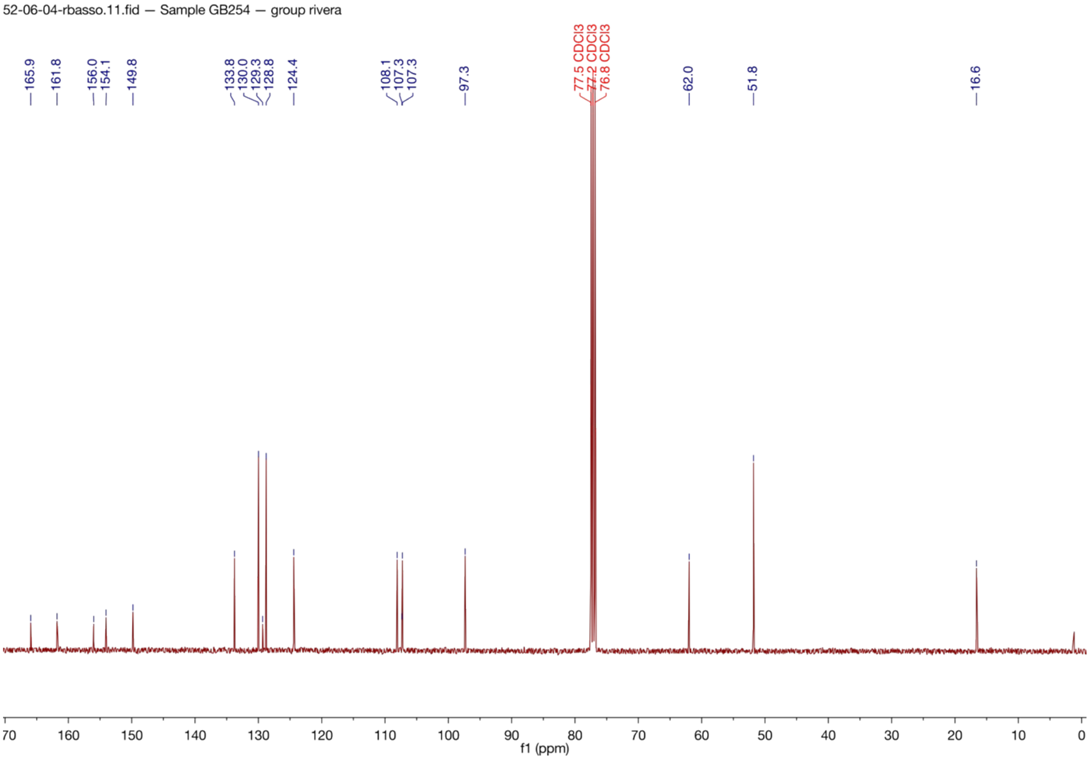

^{13}C NMR ($CDCl_3$, 100 MHz) spectrum of compound **3b**

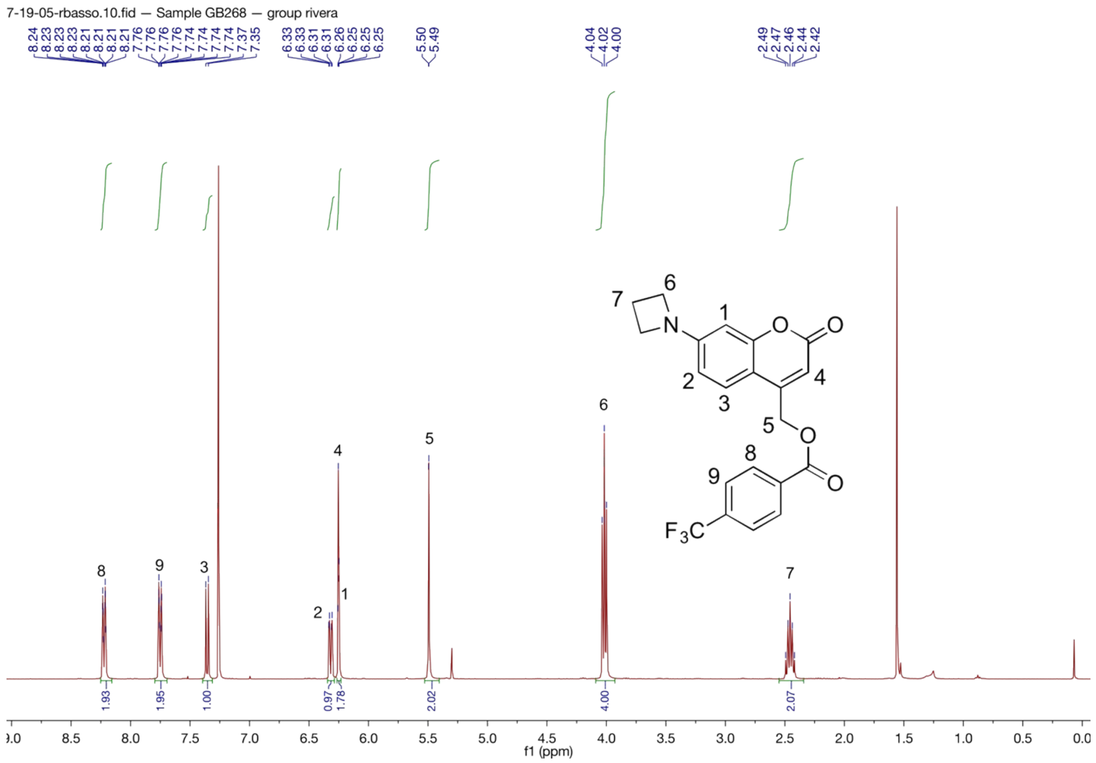

^{1}H NMR ($CDCl_3$, 400 MHz) spectrum of compound **3c**

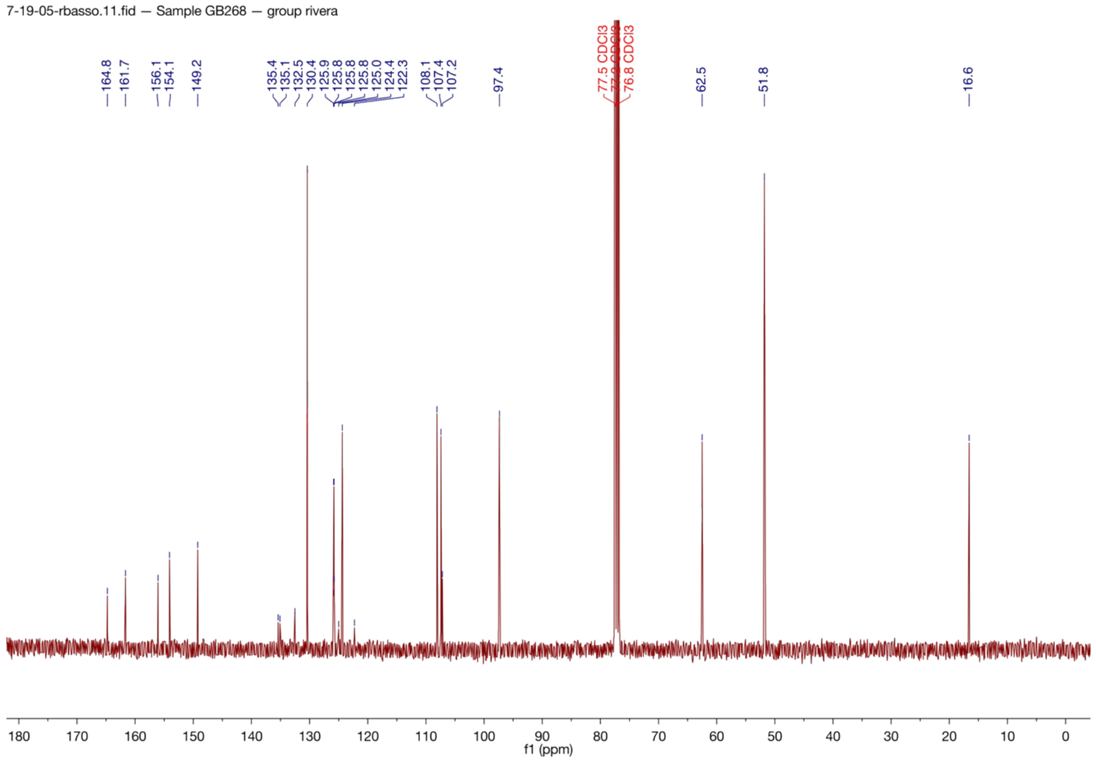

^{13}C NMR ($CDCl_3$, 100 MHz) spectrum of compound **3c**

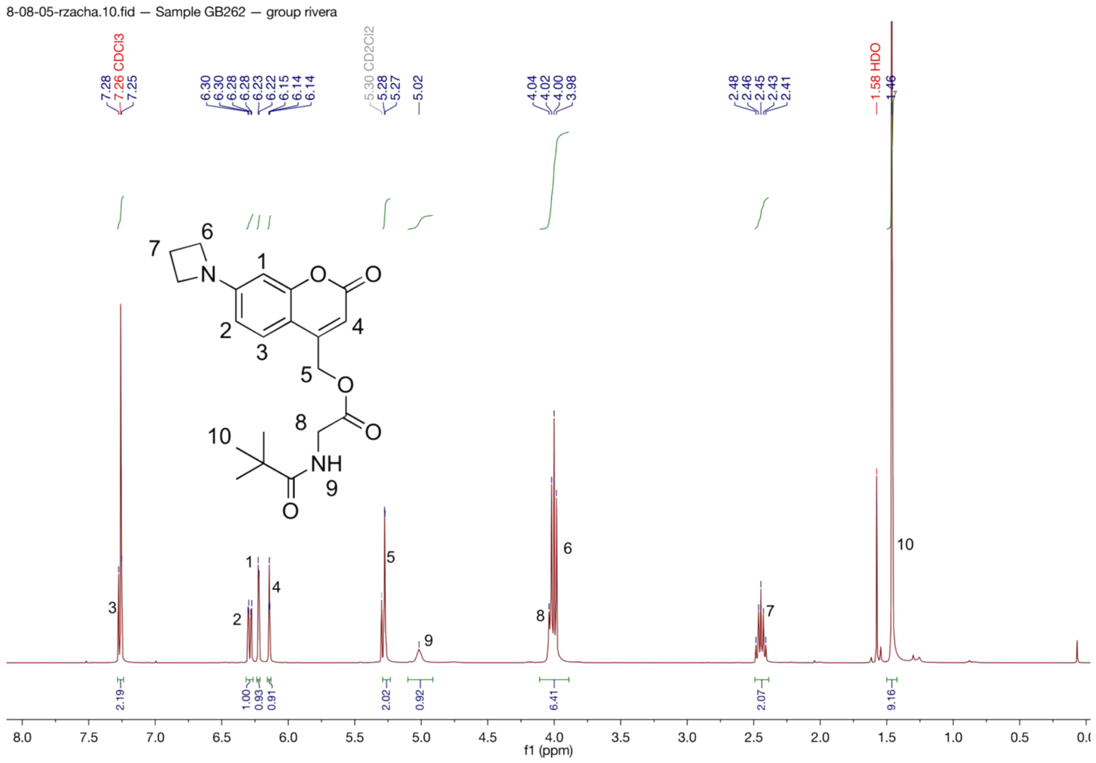

^1H NMR ($CDCl_3$, 400 MHz) spectrum of compound **3d**

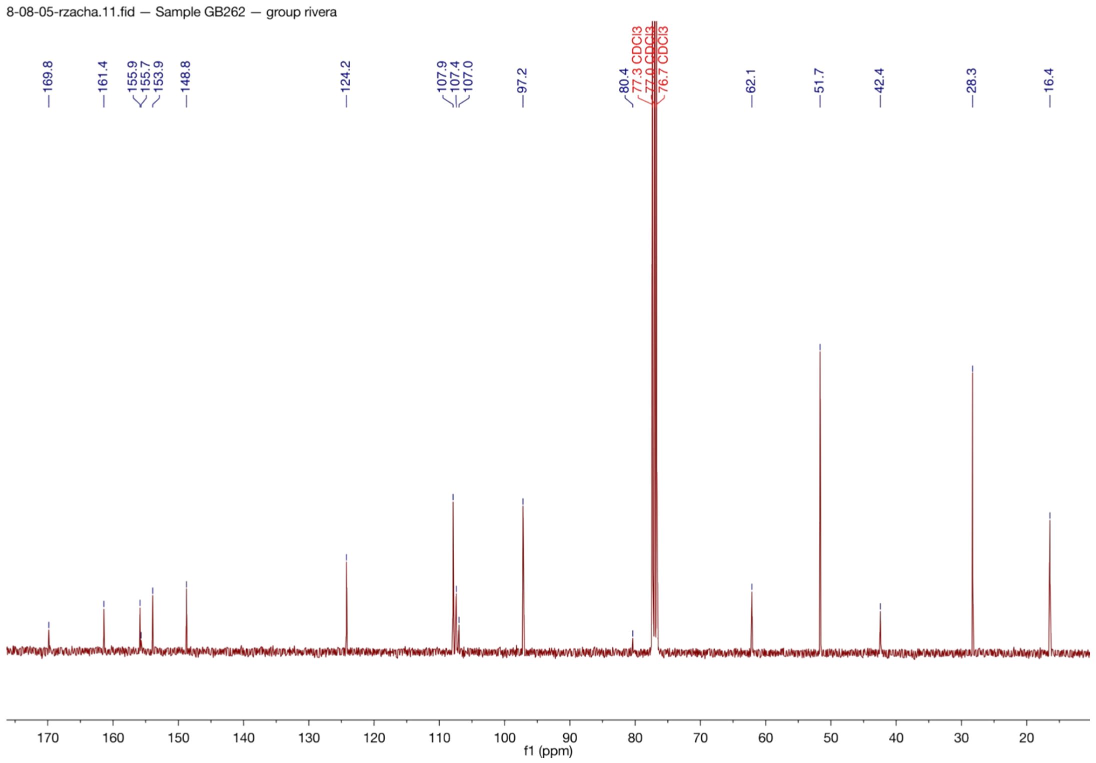

^{13}C NMR ($CDCl_3$, 100 MHz) spectrum of compound **3d**

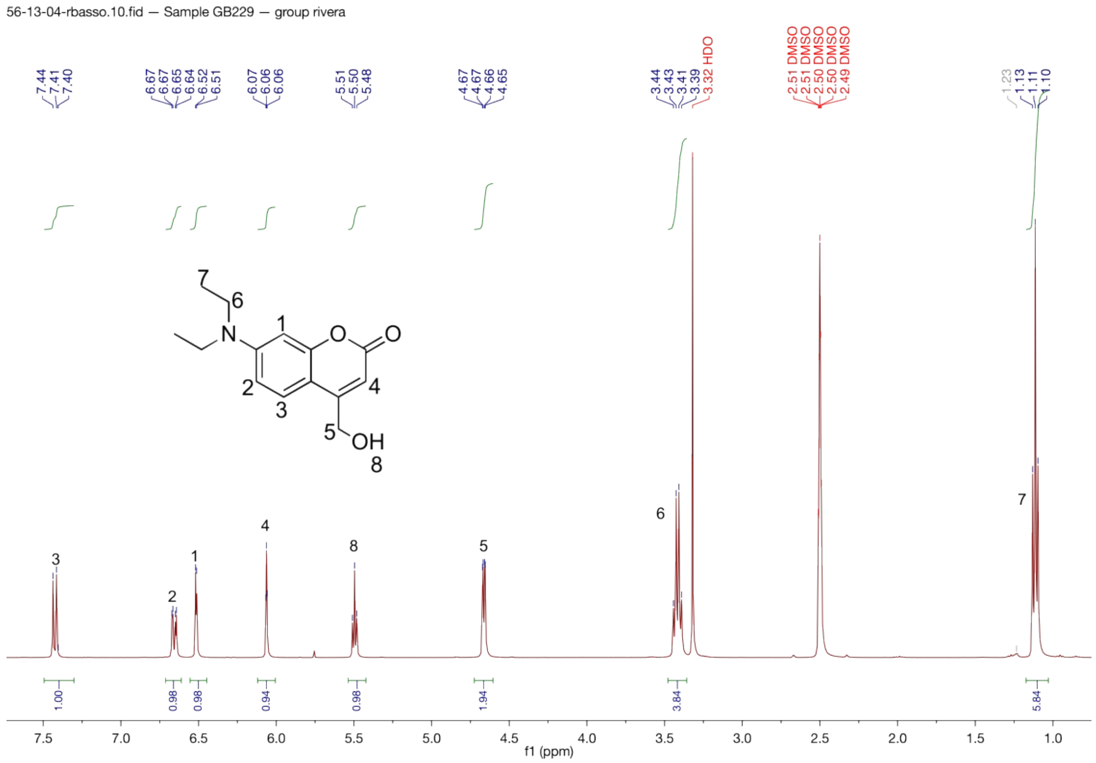

^{1}H NMR $((CD_3)_2SO$, 400 MHz) spectrum of compound **SI-3**

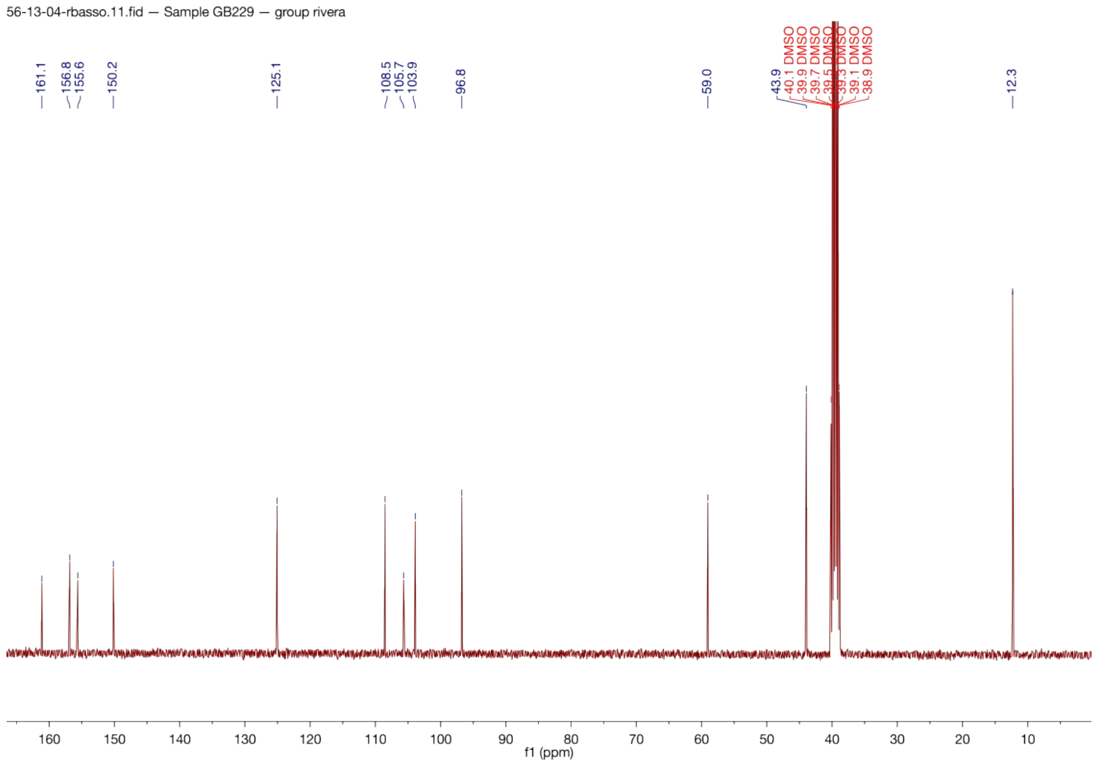

^{13}C NMR $((CD_3)_2SO$, 100 MHz) spectrum of compound **SI-3**

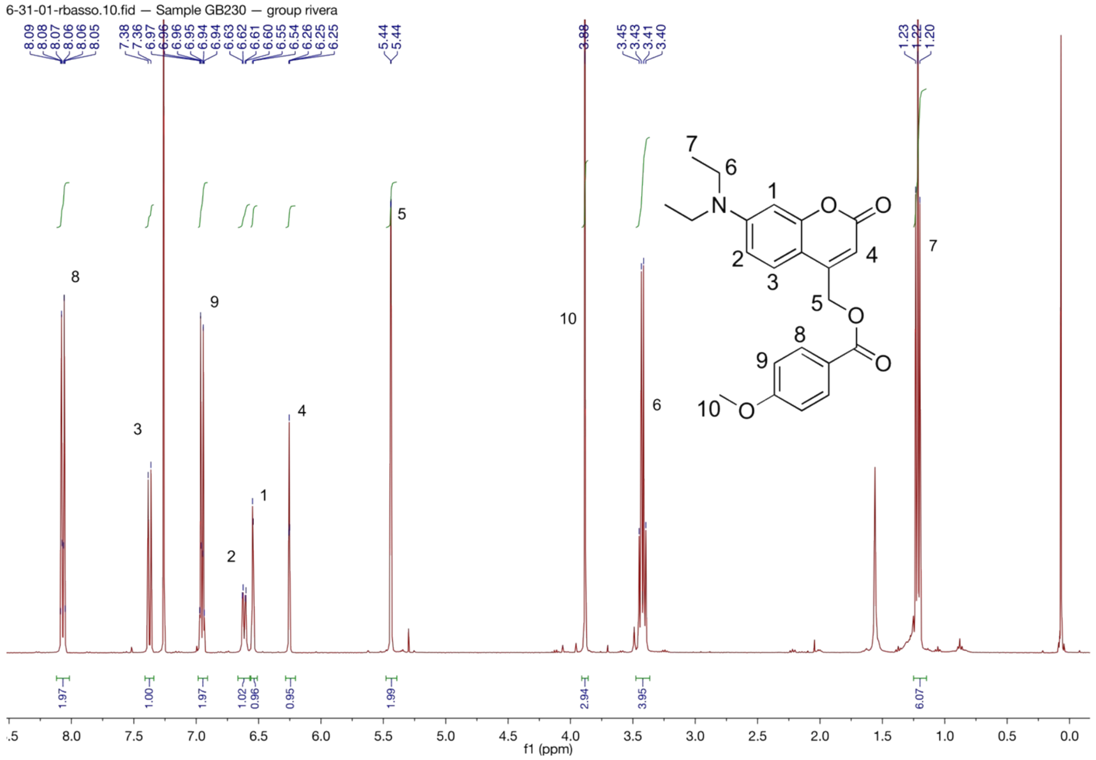

^{1}H NMR ($CDCl_3$, 400 MHz) spectrum of compound **4a**

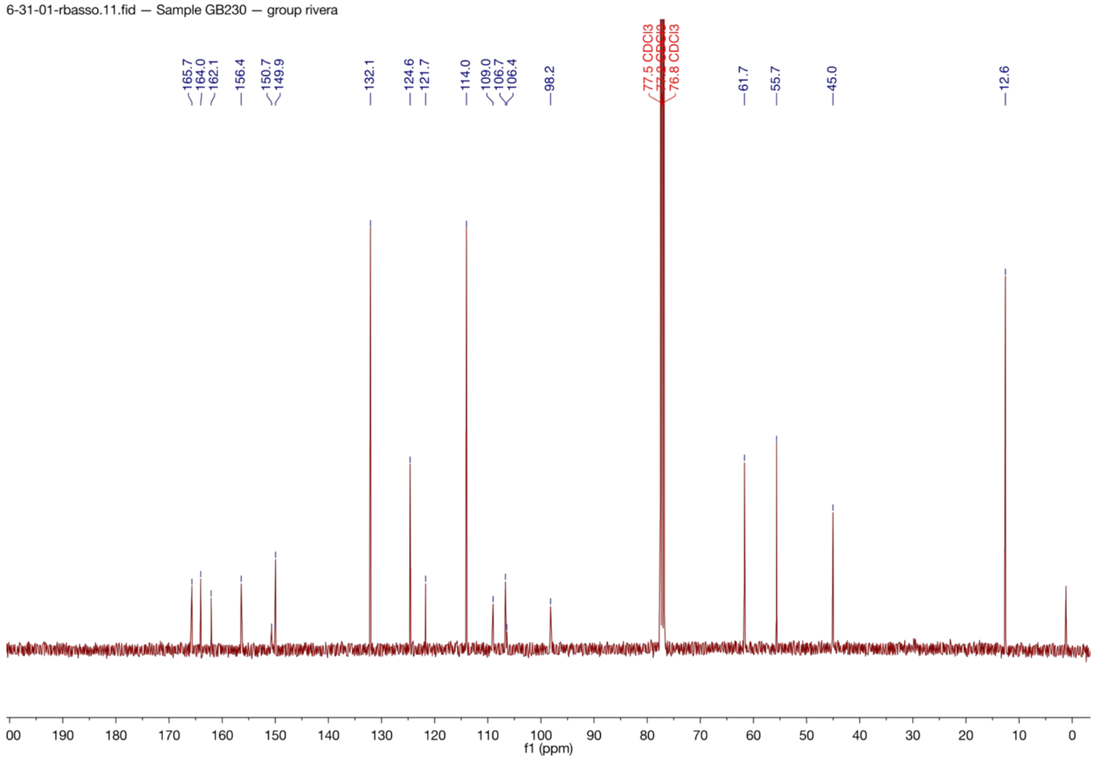

^{13}C NMR ($CDCl_3$, 100 MHz) spectrum of compound **4a**

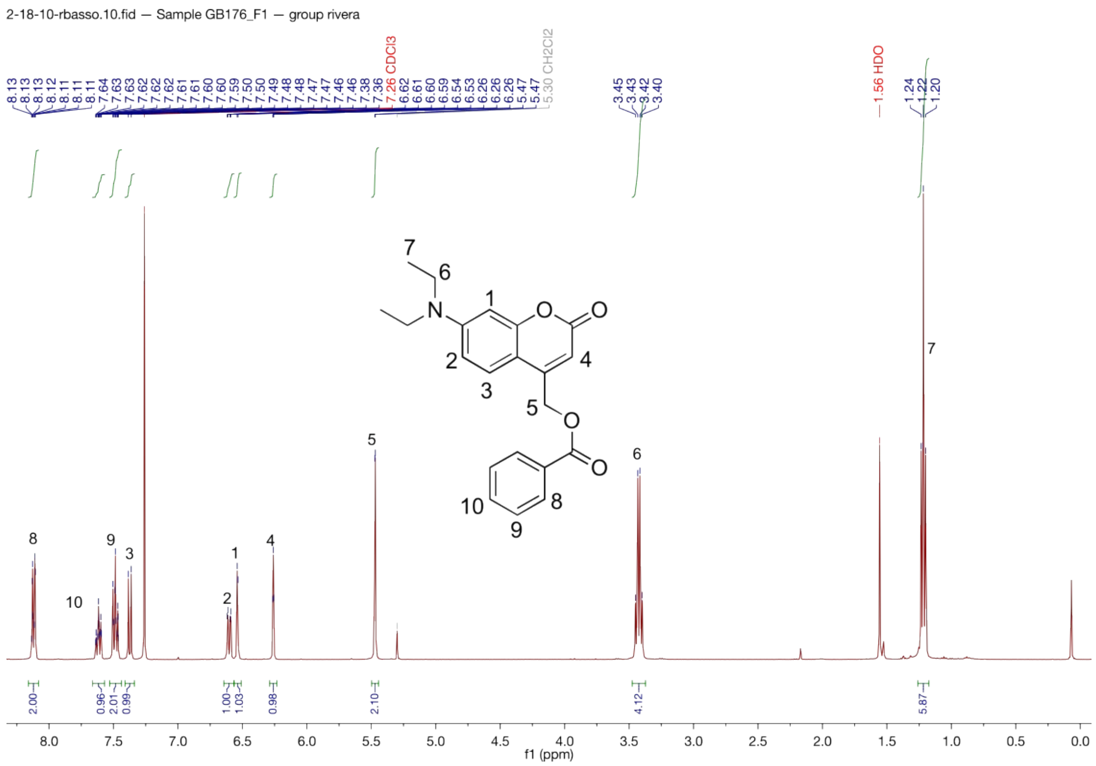

^1H NMR ($CDCl_3$, 400 MHz) spectrum of compound **4b**

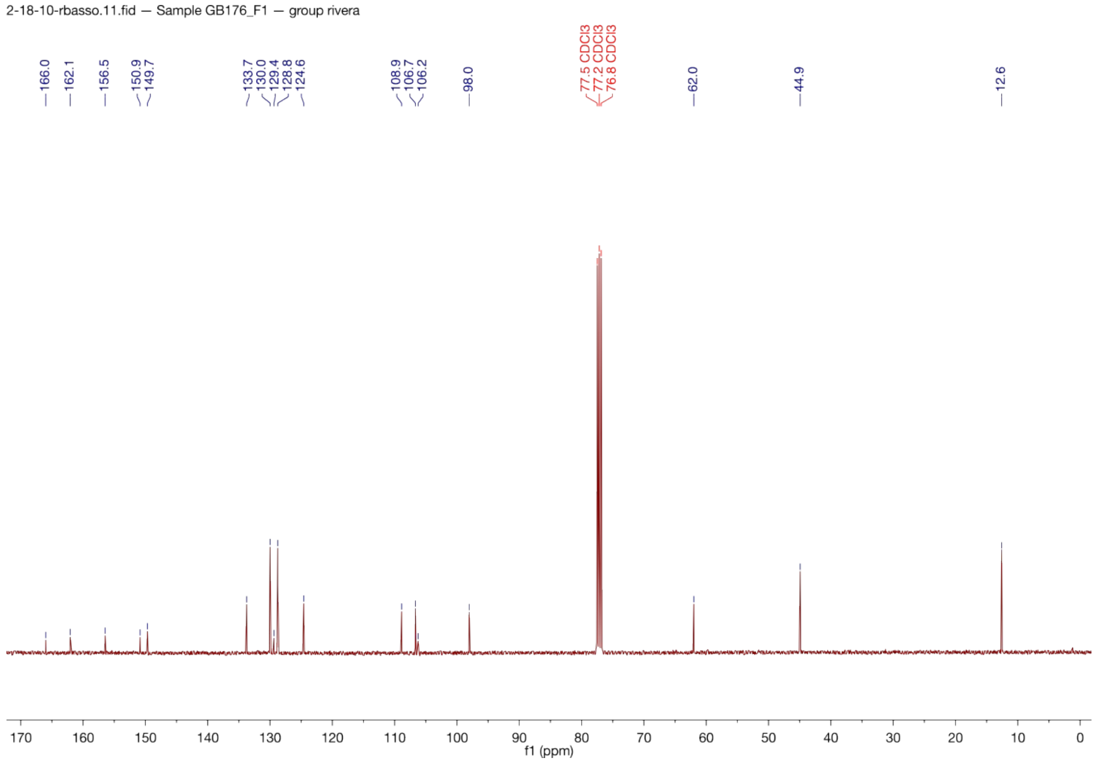

^{13}C NMR ($CDCl_3$, 100 MHz) spectrum of compound **4b**

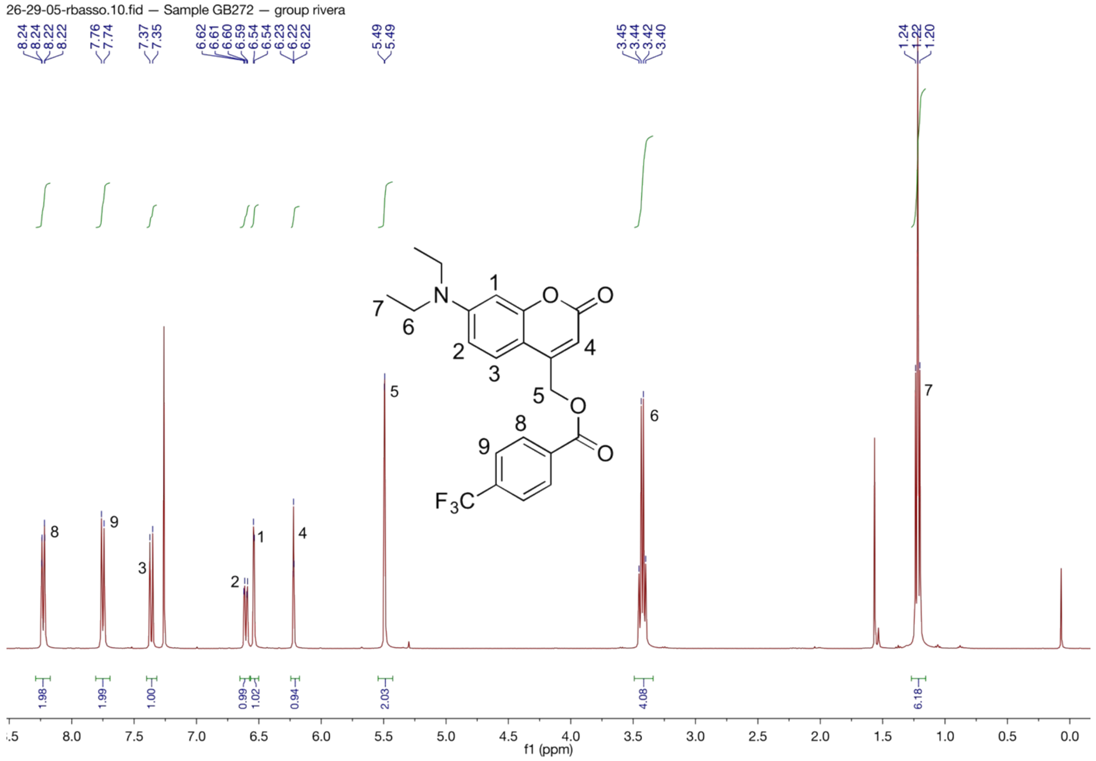

^1H NMR ($CDCl_3$, 400 MHz) spectrum of compound **4c**

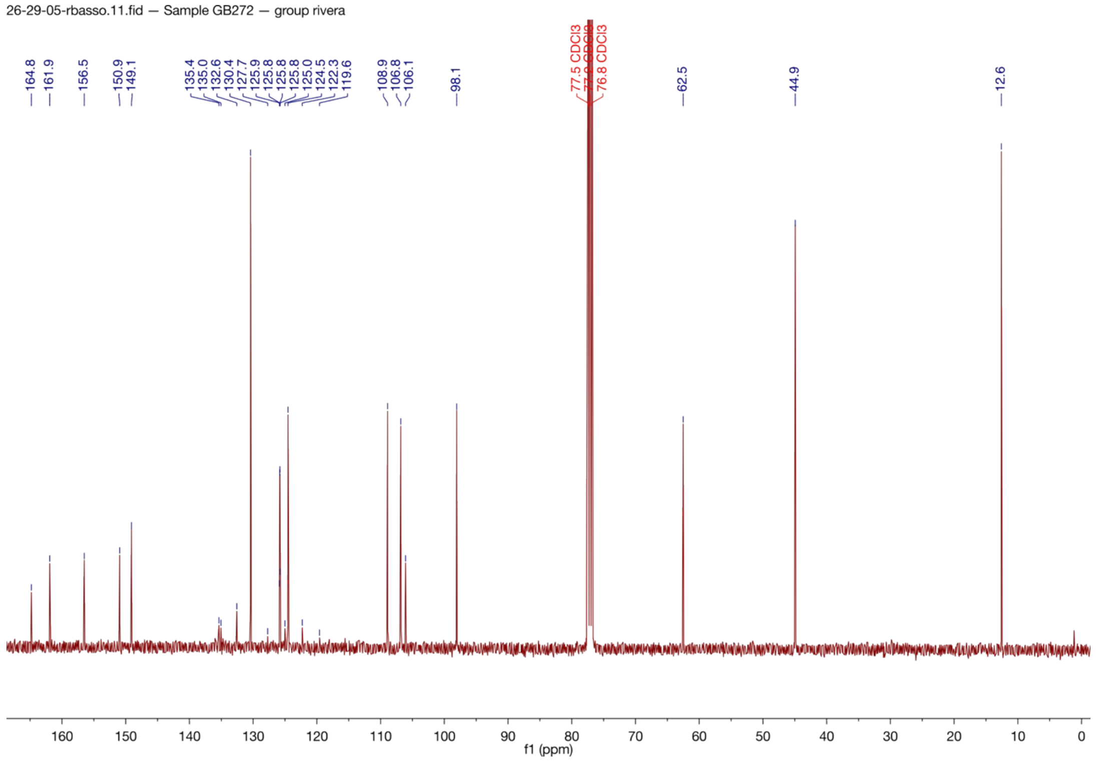

^{13}C NMR ($CDCl_3$, 100 MHz) spectrum of compound **4c**

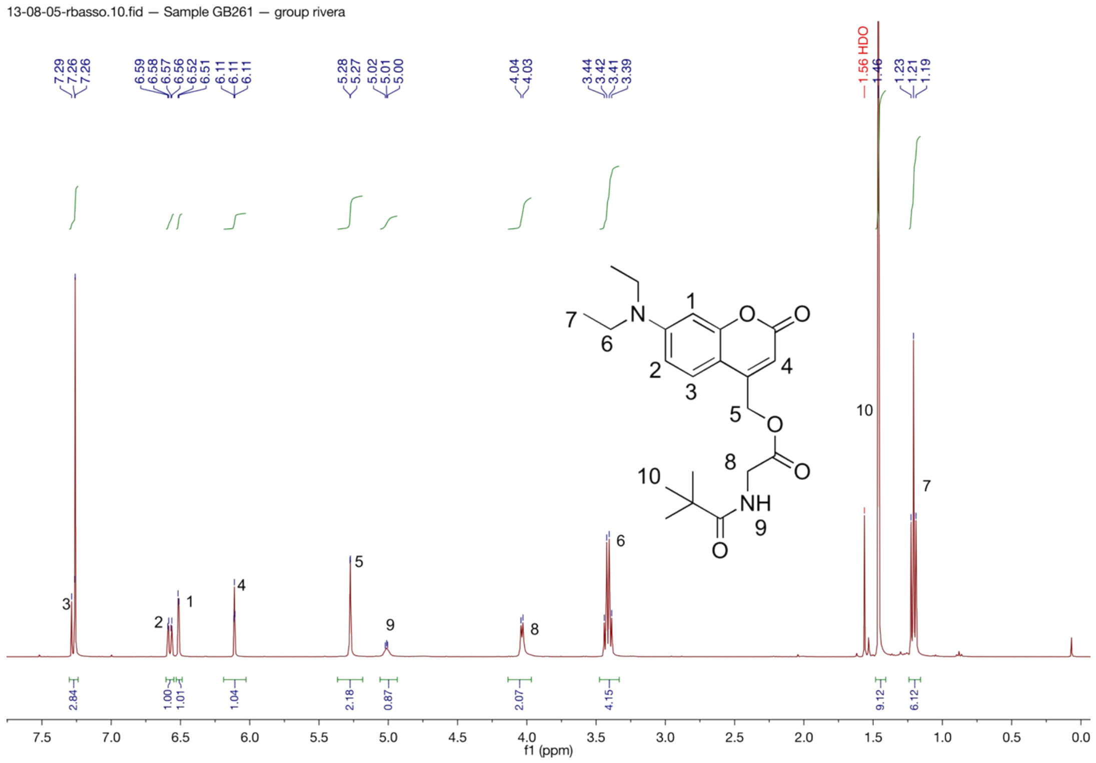

^1H NMR ($CDCl_3$, 400 MHz) spectrum of compound **4d**

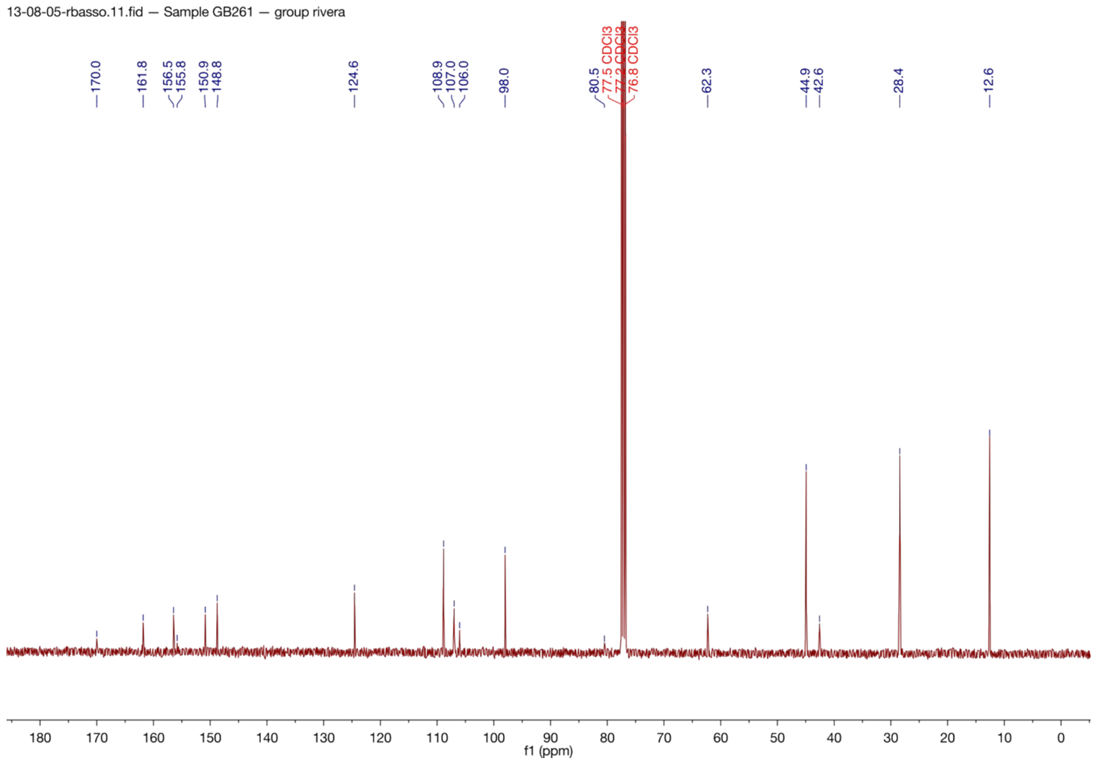

^{13}C NMR ($CDCl_3$, 100 MHz) spectrum of compound **4d**

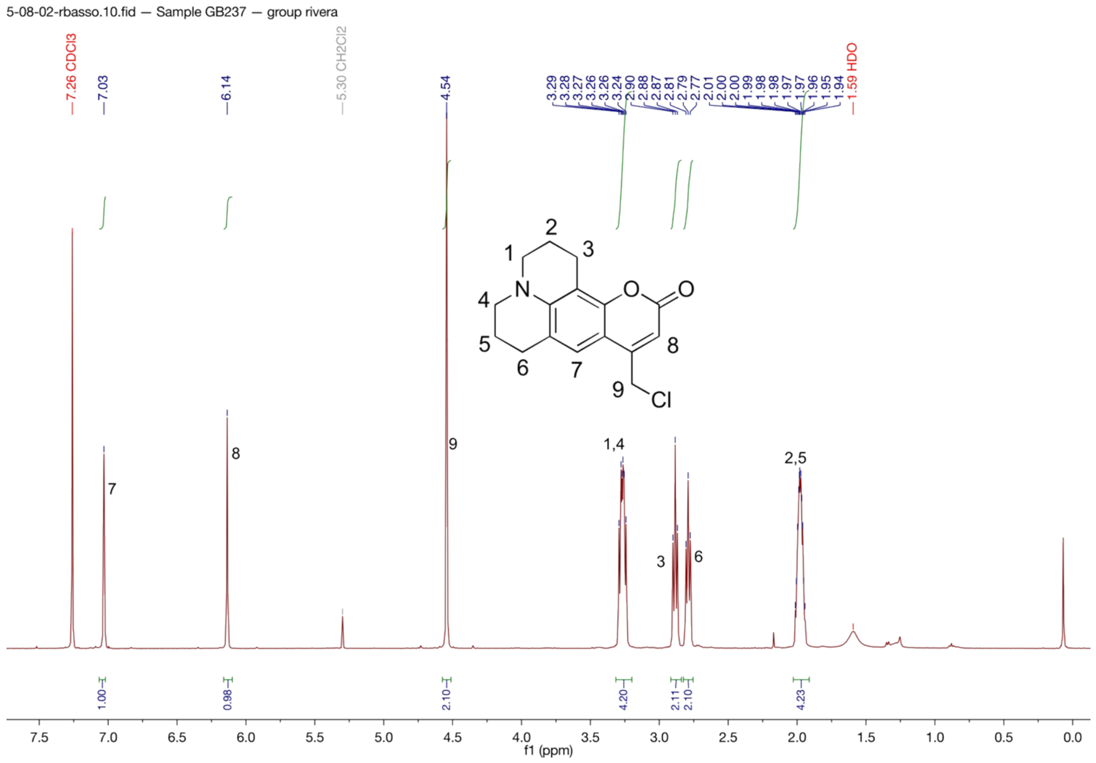

^{1}H NMR ($CDCl_3$, 400 MHz) spectrum of compound **SI-4**

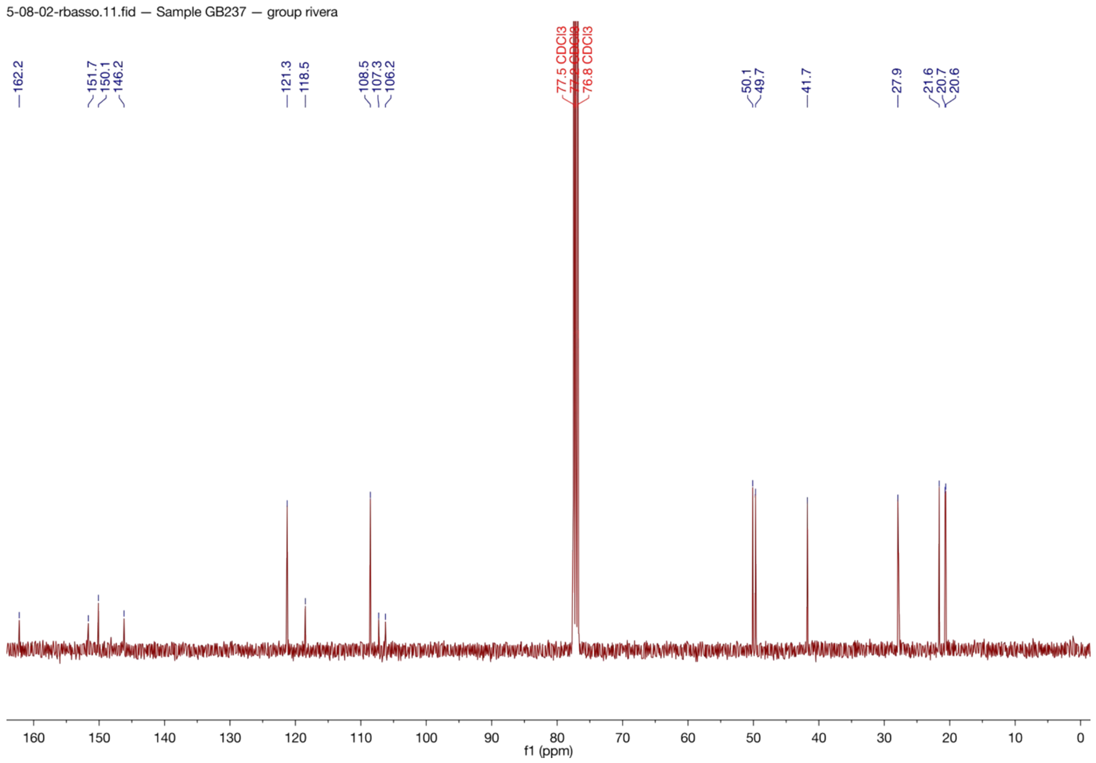

^{13}C NMR ($CDCl_3$, 100 MHz) spectrum of compound **SI-4**

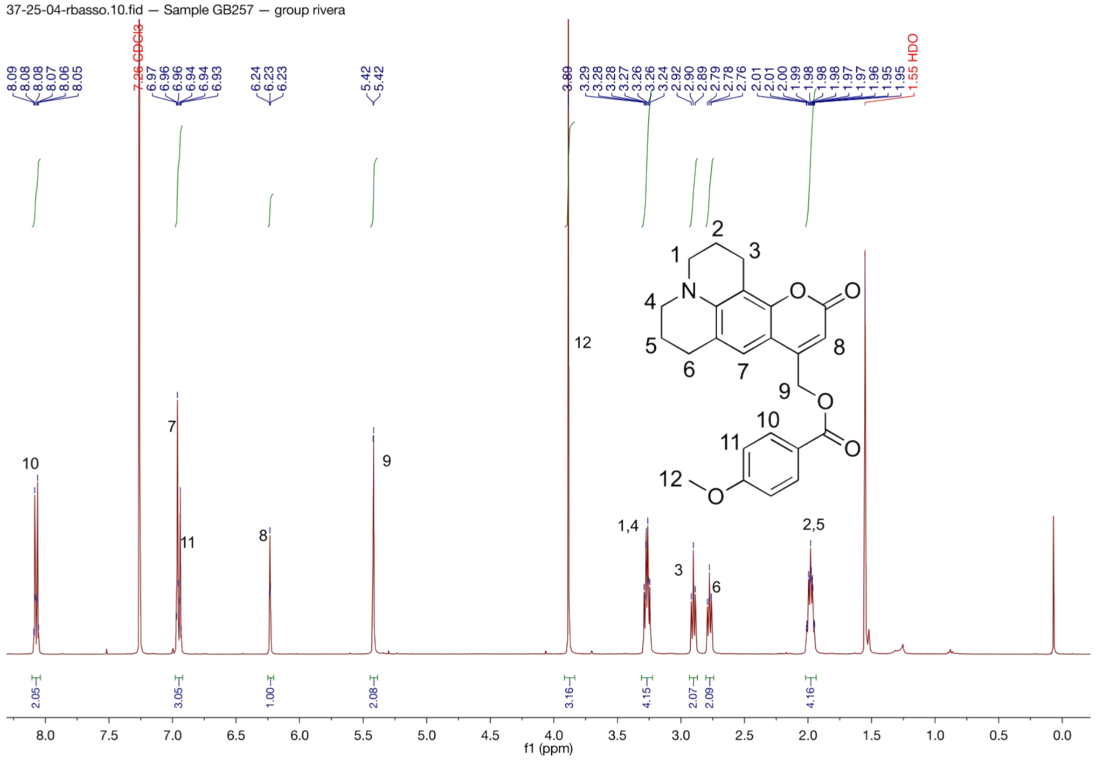

^{1}H NMR ($CDCl_3$, 400 MHz) spectrum of compound **5a**

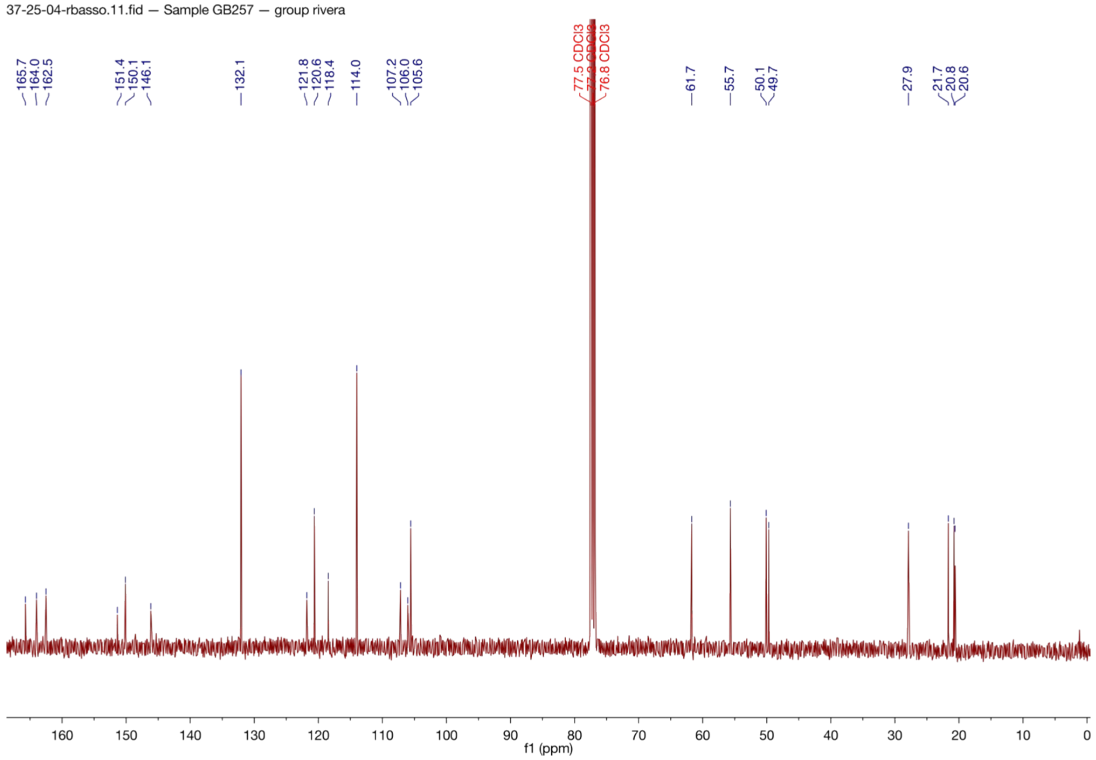

^{13}C NMR ($CDCl_3$, 100 MHz) spectrum of compound **5a**

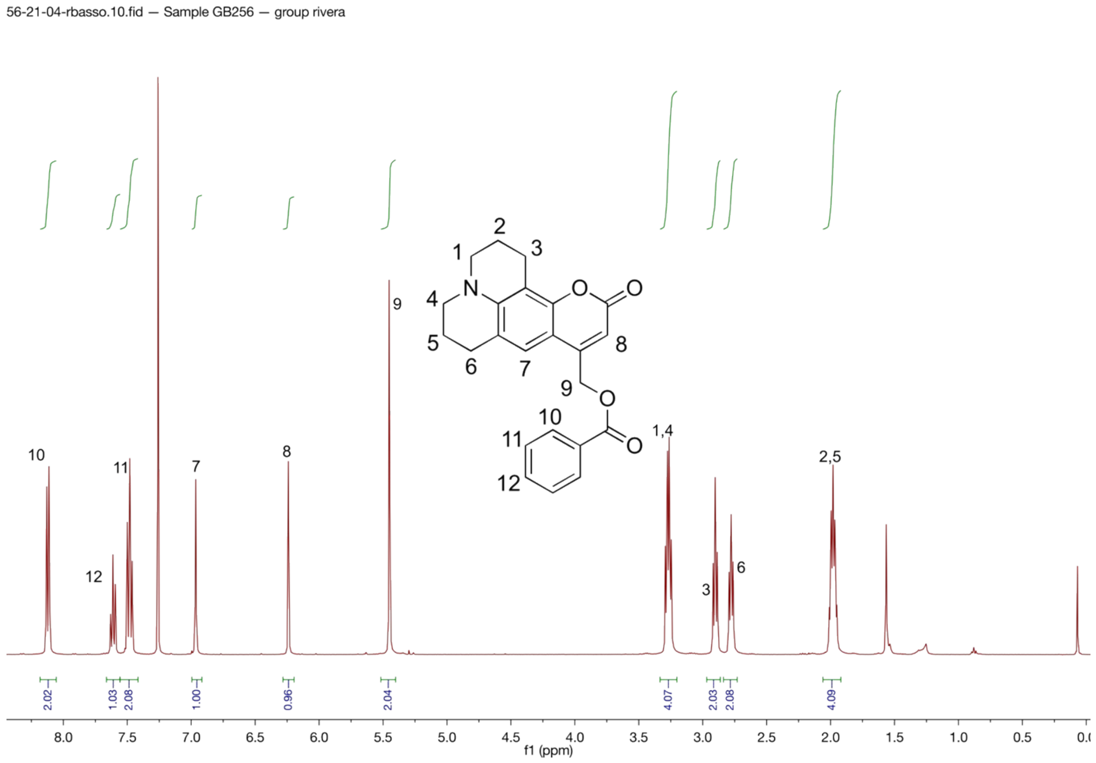

^{1}H NMR ($CDCl_3$, 400 MHz) spectrum of compound **5b**

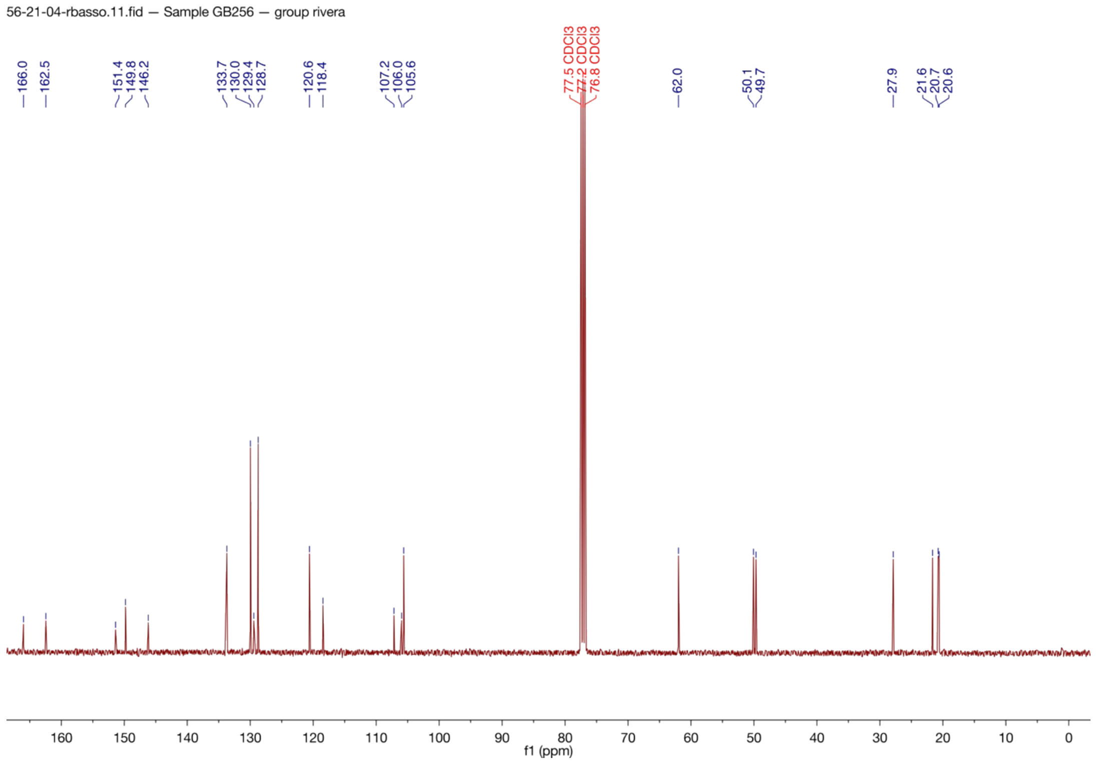

^{13}C NMR ($CDCl_3$, 100 MHz) spectrum of compound **5b**

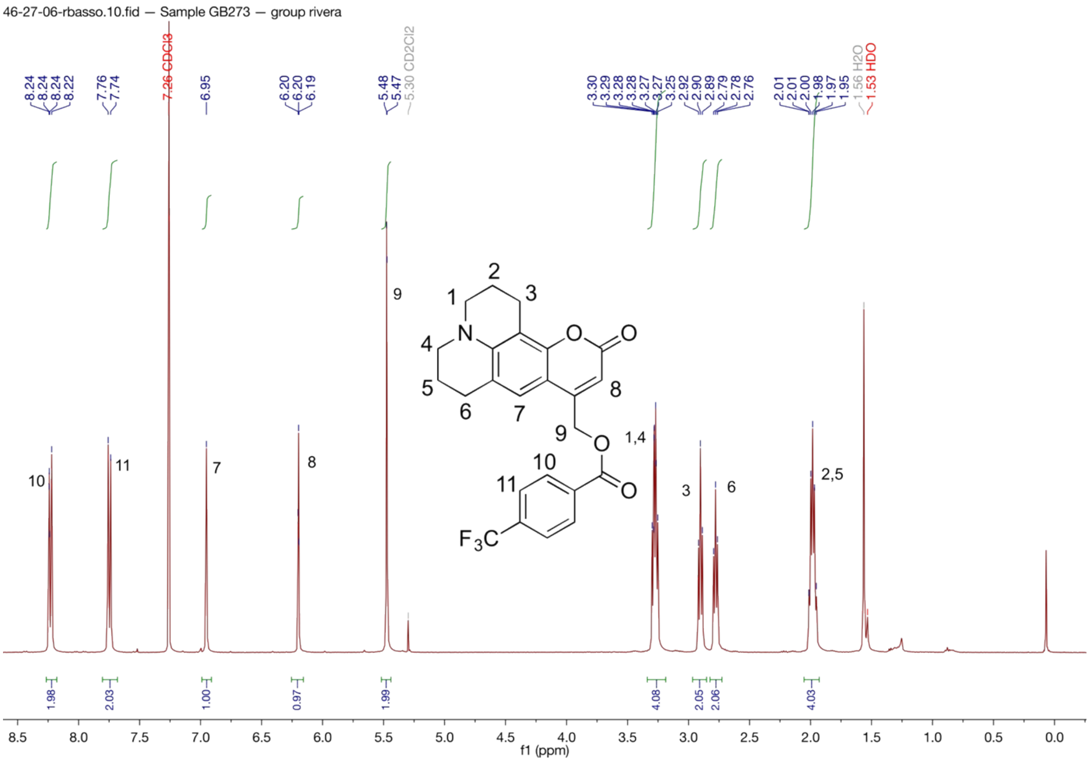

^{1}H NMR (CDCl$_3$, 400 MHz) spectrum of compound **5c**

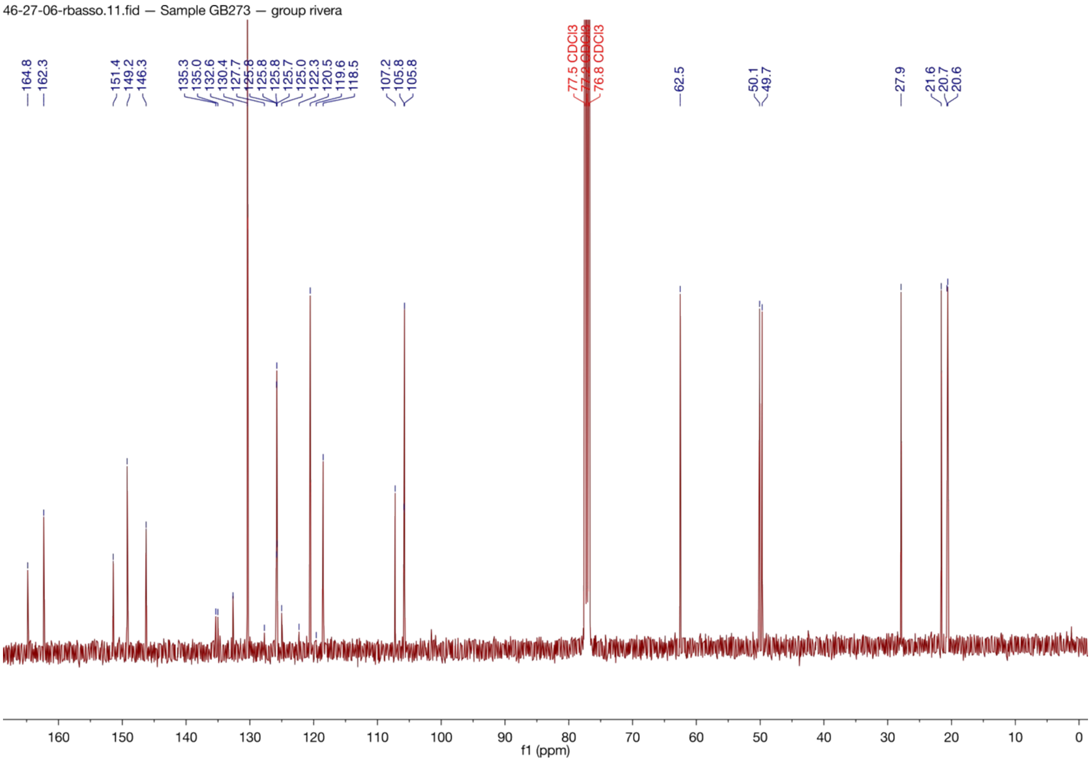

^{13}C NMR (CDCl$_3$, 100 MHz) spectrum of compound **5c**

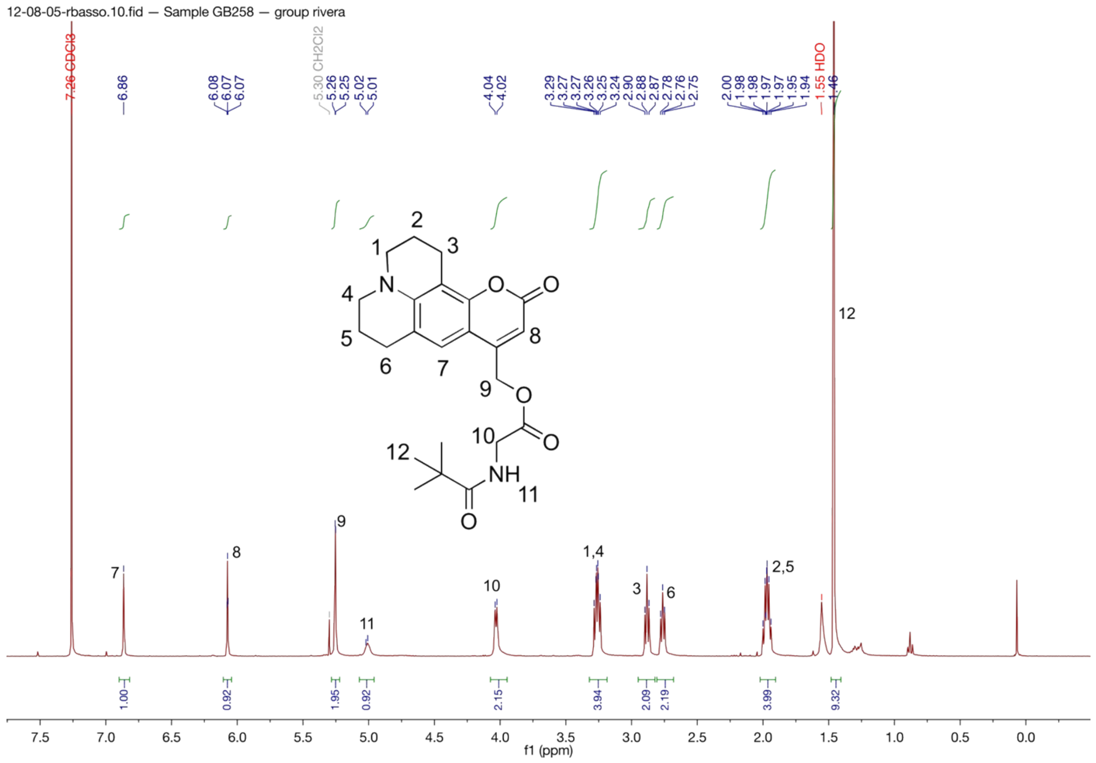

^{1}H NMR ($CDCl_3$, 400 MHz) spectrum of compound **5d**

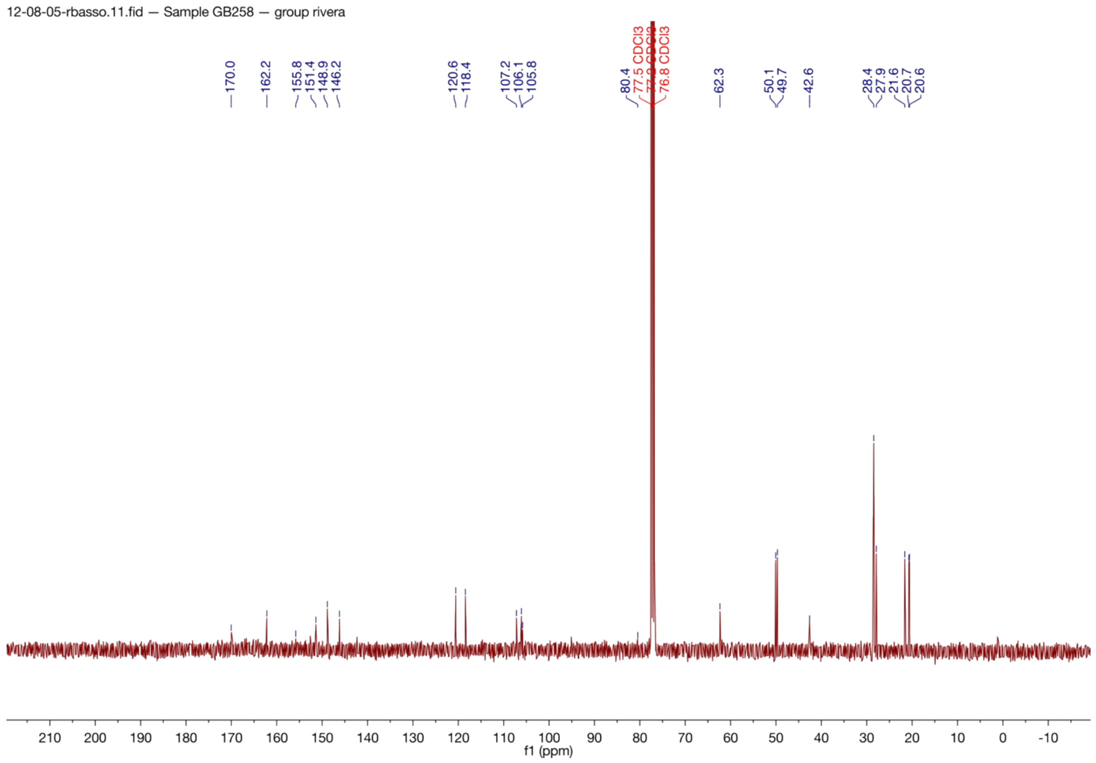

^{13}C NMR ($CDCl_3$, 100 MHz) spectrum of compound **5d**

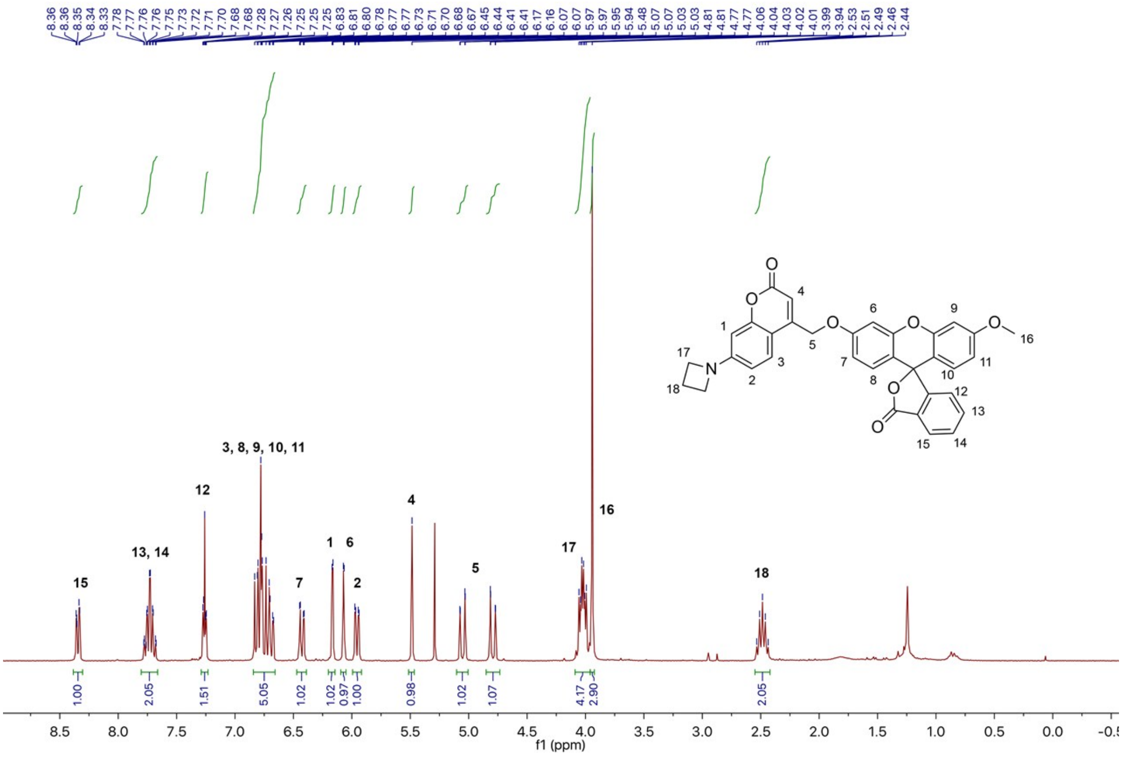

^{1}H NMR ($CDCl_3$, 300 MHz) spectrum of compound **6**

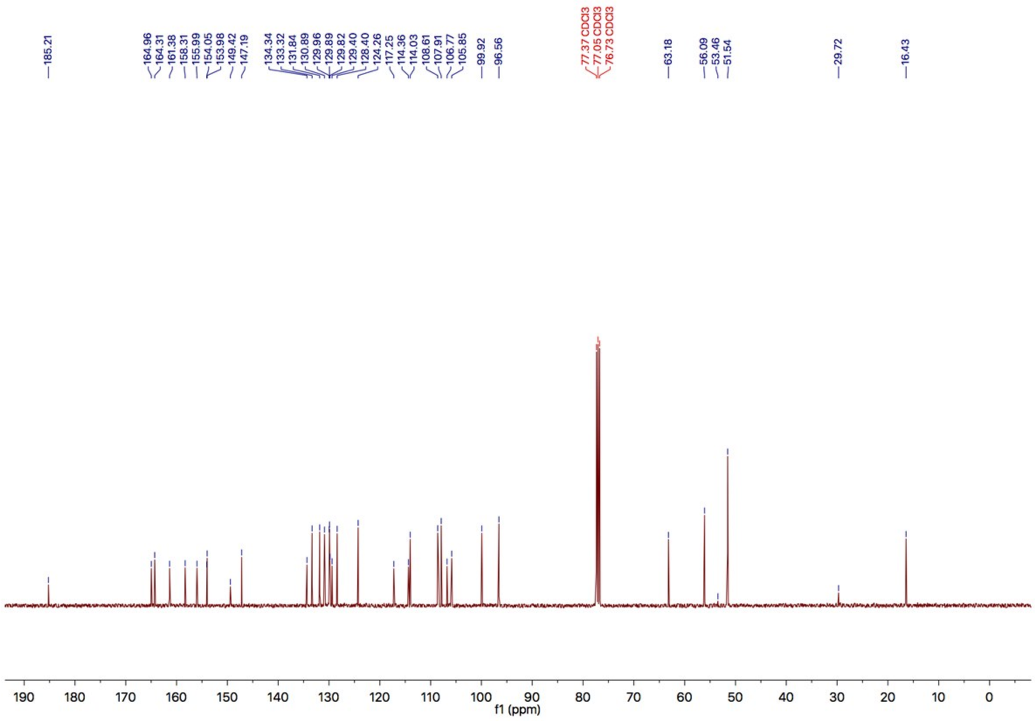

^{13}C NMR ($CDCl_3$, 100 MHz) spectrum of compound **6**
